# Organoselenium-based Schiff bases and amidic acid derivatives as promising anticancer agents targeting breast cancer by downregulating BCL-2: design, synthesis, and biological evaluation

**DOI:** 10.1039/d5ra09238h

**Published:** 2026-02-02

**Authors:** Saad Shaaban, Samia S. Hawas, Asma M. Elsharif, Marwa Sharaky, Hussein Ba-Ghazal, Mohamed Alaasar, Fatema S. Alatawi, Khadra B. Alomari, Mohamed E. Eissa, Arwa Omar Al Khatib, Radwan Alnajjar, Ahmed A. Al-Karmalawy

**Affiliations:** a Department of Chemistry, College of Science, King Faisal University Al-Ahsa 31982 Saudi Arabia sibrahim@kfu.edu.sa; b Department of Pharmaceutical Chemistry, Faculty of Pharmacy, Horus University-Egypt New Damietta 34518 Egypt; c Department of Chemistry, College of Science, Imam Abdulrahman Bin Faisal University Dammam 31441 Saudi Arabia; d Cancer Biology Department, Pharmacology Unit, National Cancer Institute (NCI), Cairo University Cairo Egypt; e Department of Chemistry, Faculty of Science, Cairo University Giza Egypt; f Department of Biochemistry, Faculty of Science, University of Tabuk Tabuk Saudi Arabia; g Jazan University, Department of Physical Sciences, Chemistry Division P.O. Box 114 Jazan 45142 Kingdom of Saudi Arabia; h Department of Chemistry, College of Science, Imam Mohammad Ibn Saud Islamic University (IMSIU) Riyadh 11623 Saudi Arabia; i Faculty of Pharmacy, Al-Ahliyya Amman University Amman Jordan; j Department of Chemistry, Faculty of Science, University of Benghazi Benghazi Libya; k CADD Unit, PharmD, Faculty of Pharmacy, Libyan International University Benghazi Libya; l Department of Pharmaceutical Chemistry, College of Pharmacy, The University of Mashreq Baghdad 10023 Iraq akarmalawy@horus.edu.eg

## Abstract

This study reports the biological evaluation of novel Schiff base-tethered organoselenium (OSe) compounds as potential anticancer agents. New derivatives (HB178, HB179, HB181, HB183, HB208, HB209, and HB210) were synthesized and screened for cytotoxicity against eight cancer cell lines (including HN9, FaDu, MCF7, A375, HEPG2, HuH7, A549, and HCT_116_) and two normal cell lines (OEC and HSF). Among them, HB183, HB209, and HB210 exhibited the most potent growth inhibition (GI) activity, with average values of 78.25%, 76.34%, and 79.14%, respectively—surpassing the reference drug doxorubicin (61.89%). HB183 demonstrated the strongest cytotoxic effects, with IC_50_ values of 9.72 µM (MCF7), 13.28 µM (HCT_116_), 13.50 µM (A549), and 31.28 µM (HEPG2), significantly outperforming doxorubicin across multiple cell lines. Importantly, HB183 showed selective cytotoxicity with lower GI% values against normal OEC (53.90%) and HSF (42.27%) cells. Mechanistic investigations revealed that HB183 upregulated key pro-apoptotic proteins—BAX (1.39-fold), caspase-3 (1.18-fold), caspase-7 (1.20-fold), and caspase-9 (1.45-fold)—while downregulating anti-apoptotic markers such as BCL-2 (1.22-fold), MMP2 (1.15-fold), and MMP9 (1.30-fold). Furthermore, flow cytometry analysis indicated that HB183 induced cell cycle arrest at the pre-G1 phase in MCF7 cells, increasing the population from 94.32% to 98.84%. Molecular docking, molecular dynamics simulation (for 500 ns), and MM-GBSA calculations for the lead analogue (HB183) towards the BCL-2 target, as a crucial one in the pathway of apoptosis induction, were performed to support the mechanistic investigation. These findings suggest that HB183 is a promising lead for further development as a selective and potent anticancer agent, particularly in the treatment of breast cancer.

## Abstract 2

Organoselenium-based Schiff bases and amidic acid derivatives targeting breast cancer by down regulating BCL-2.

## Graphical abstract

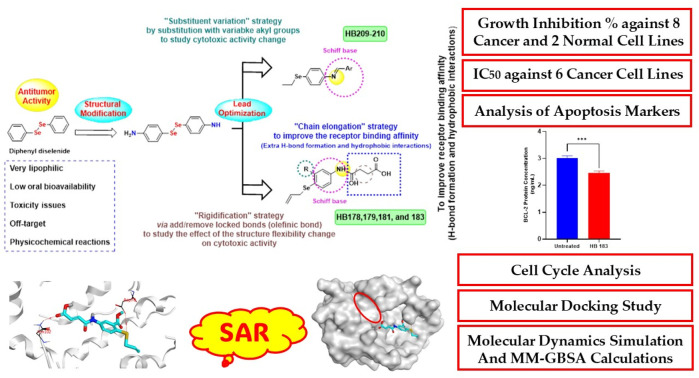

## 1. Introduction

Breast cancer is a leading cause of cancer-related mortality in women, and one of the most commonly diagnosed cancers worldwide.^1–4^ Despite significant advances in therapeutic strategies, current treatment regimens often encounter challenges such as drug resistance, adverse side effects, and limited selectivity toward malignant cells.^5–9^ These ongoing challenges underscore the importance of discovering new therapeutic agents that can selectively target cancer pathways, minimizing systemic toxicity.^10–12^

Over the last decade, organoselenium (OSe) compounds have gained attention as chemotherapeutic and chemopreventive agents, particularly for their redox-modulating, antioxidant, and apoptosis-inducing properties.^13–18^ Selenium plays a vital biological role as an essential trace element, functioning within key antioxidant enzymes to protect cells from oxidative stress. Supplementation with selenium and the development of selenium-containing compounds have shown promise in preventing or treating various cancers, including prostate, lung, liver, and breast tumors.^18–23^

**Fig. 1:**
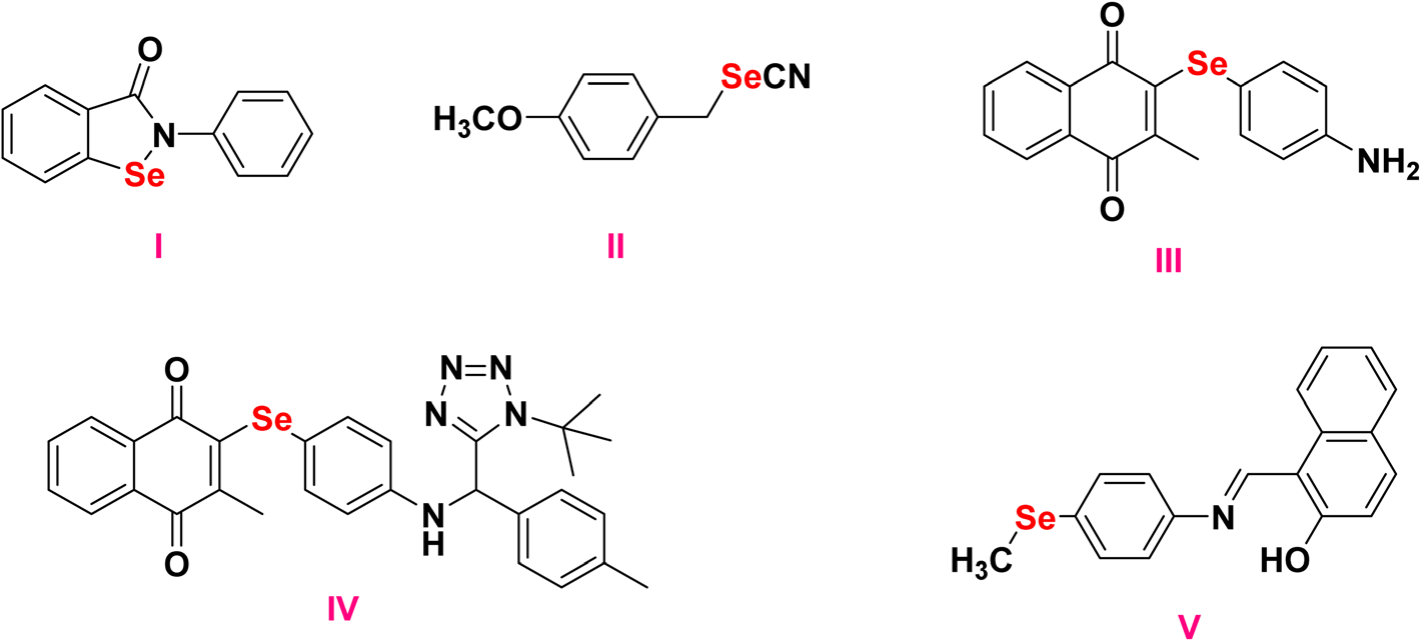
Organoselenium compounds that show promise in combating cancer.

Among the promising examples are compound I (ebselen), present in Fig. 1, a heterocyclic OSe compound currently undergoing clinical trials, and compound II (BSeC), present in Fig. 1, a representative organic selenocyanate, both of which have demonstrated effective chemopreventive activities in oxidative stress-related diseases.^24,25^ Additionally, selenoquinones III and IV, present in Fig. 1, have been reported to upregulate interleukin 2/6, caspase-8, and CD40, while suppressing BCL-2 and Ki-67, thereby enhancing apoptosis and reducing proliferation in liver cancer models.^26,27^

Parallel to OSe developments, azomethines (Schiff bases, imines) have long been used for their diverse biological applications, including antimicrobial, anti-inflammatory, anticonvulsant, and antitumor effects.^28–31^ Their biological activity is attributed to the ability of the imine nitrogen (–CH=N–) to form hydrogen bonds with intracellular biomolecules, particularly proteins and enzymes, making them valuable tools in modulating cellular function and designing hybrid molecules.^32,33^ Newly synthesized organoselenide-based Schiff base V, present in Fig. 1, was proven to be effective against colorectal cancer through the induction of apoptosis of cancer cells by upregulation of BAX, P53, and caspases, and downregulation of BCL-2, MMP2, and MMP9.^33,34^

**Fig. 2:**
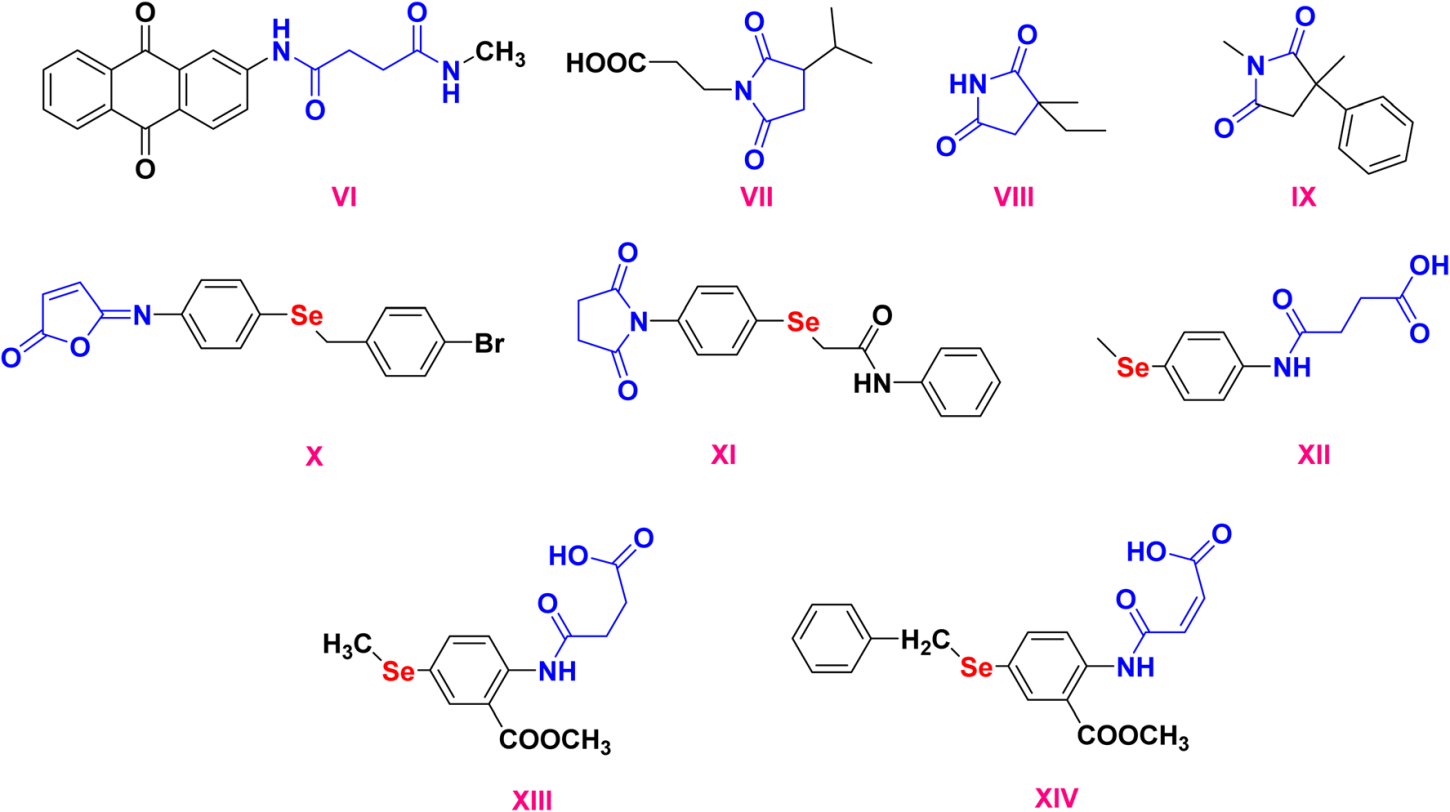
Cyclic imides, amidic acid, and dicarboxamide are biologically significant compounds.

Recent efforts have explored dicarboxamide-containing scaffolds, such as compound VI (1,3-dicarboxamide), known for their antioxidant activity *via* cytochrome P450 upregulation and their use as ligands in coordination chemistry and heavy metal sensing.^35^ The relevance of maleic and succinic acid derivatives in drug design is well demonstrated by compound VII (farinomalein), a natural maleimide pesticide, compound VIII (zarontin), an approved succinimide-based anticonvulsant, and compound IV (oxaleimide A), an active pharmaceutical compound.^36–38^ These structures highlight the biomedical importance of amidic acids as versatile functional groups,^39^Fig. 2.

Building on this, our group and others have focused on hybrid compounds that merge selenium-based cores with amide, Schiff base, or maleimide/succinimide groups. For example, the isomaleimide X, synthesized in our lab, displayed antioxidant, cytoprotective, and anti-apoptotic properties in neuronal cells.^40–42^ Likewise, compound XI, an *N*-succinimide selenium derivative, showed promising anticancer activity in HepG2 liver carcinoma cells, while compound XII, bearing an *N*-maleanilic acid moiety, demonstrated both antioxidant and antimicrobial activity, and cytotoxicity against MCF-7, HCT_116_, and HepG2 cells, Fig. 2.^14,43–45^

Dysregulation of the intrinsic apoptotic pathway, particularly *via* overexpression of the anti-apoptotic protein BCL-2, has been widely implicated in breast cancer progression and resistance to therapy.^46,47^ Inhibitors that shift the balance toward pro-apoptotic signaling (*e.g.*, increasing BAX/BCL-2 ratio and activating caspases) are recognized as promising anticancer strategies, as exemplified by clinically approved BCL-2 inhibitors (*e.g.*, venetoclax).^48,49^ However, the development of small molecules targeting BCL-2 must carefully balance efficacy with potential selenium-related toxicity, as selenium's redox activity can induce both therapeutic and off-target effects at higher doses.^50^ Organoselenium compounds, such as ebselen and related analogues, have demonstrated anticancer activity at low-to-mid micromolar concentrations with manageable toxicity profiles, supporting their continued investigation as apoptosis inducers.^51,52^ In this context, integrating organoselenium cores with imine and amidic motifs provides a rationale for designing hybrid molecules that may engage mitochondrial apoptotic pathways, modulate BCL-2 family proteins, and promote selective cancer cell death.^53^

Taken together, these findings underscore the value of integrating OSe, Schiff base, and amidic acid motifs within a single molecular framework. Such hybridization offers the potential for synergistic biological effects, improved pharmacological profiles, and targeted action on cancer-relevant pathways.^54,55^ Accordingly, the present study focuses on the design and synthesis of a new batch of selenium-containing molecules related to compounds X, XI, and XII, incorporating Schiff base and amidic acid functionalities. These hybrids were assessed for their anticancer activity, particularly against breast cancer models with an emphasis on apoptosis induction, molecular docking with apoptosis-related targets, molecular dynamics simulation, and MM-GBSA calculations to support their candidacy for further drug development.^14,43–45^*N*-amidic acid derivatives XIII and XIV were recently synthesized and found to be apoptosis inducers in melanoma cancer *via* BCL-2, BAX, P53, caspases, MMP2, and MMP9 modulations, Fig. 2.^56^

### 1.1. Rationale of the work design

Despite the promising biological effects of diphenyl diselenide, its therapeutic application has been limited due to unfavorable physicochemical properties, including high lipophilicity, low aqueous solubility, and poor oral bioavailability, which negatively impact systemic administration and pharmacokinetics.^22,57^ To overcome these limitations and enhance antitumor efficacy, a rational drug design approach was implemented. The parent compound was structurally simplified to 4-aminophenyl diselenide, providing a synthetically accessible and versatile scaffold.^58,59^

From this new lead, several optimization strategies were pursued to improve drug-like properties and biological performance. One approach involved substituent variation at the –SeH functionality by introducing different alkyl or aryl groups, which allowed modulation of steric and electronic environments, influencing lipophilicity, metabolic stability, and binding interactions, while Schiff base derivatives enabled integration of diverse aromatic moieties to fine-tune receptor interactions and explore structure-activity relationships.^60^

Another strategy focused on chain elongation, incorporating a butanoic acid-derived amide linkage at the para position of the phenyl ring to enhance hydrogen bonding and hydrophobic interactions within target receptors, while also introducing potential sites for ionic interactions or prodrug derivatization.^61^

Additionally, molecular rigidification was applied through olefinic (C=C) bonds in the extended aliphatic chain, reducing rotational freedom to favor bioactive conformations and increase binding specificity.^61^ Aromatic rings added through Schiff base formation further contributed to lipophilicity and π–π stacking interactions, and the imine nitrogen was positioned to act as a hydrogen bond acceptor.

**Fig. 3:**
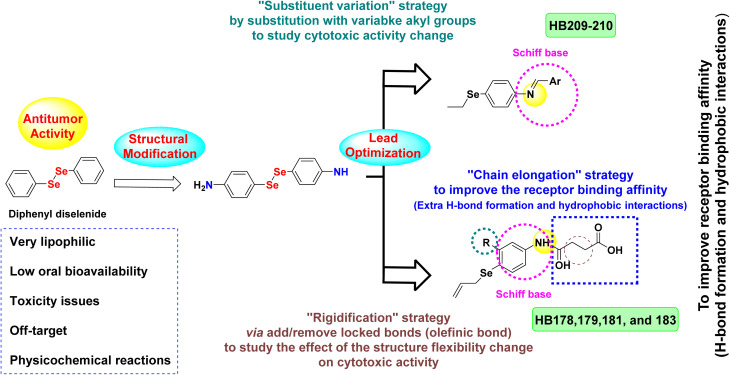
Design rationale for the newly evaluated organoselenium compounds.

Collectively, these structure-guided modifications were aimed at producing more potent and selective anticancer agents, generating structurally diverse analogues capable of multitarget interaction, and supporting the hypothesis that Schiff base-tethered organoselenium hybrids could offer synergistic cytotoxic mechanisms, enhanced selectivity, and reduced side effects relative to the original diphenyl diselenide (Fig. 3).

## 2. Results and discussion

### 2.1. Chemistry

Although the preparation of OSe candidates has attracted much interest in the last decade, owing to their numerous pharmacological activities and broad applications,^62–65^ their synthesis often encounters challenges, as many reported protocols require the use of expensive and hazardous reagents such as Na_2_Se_2_, Na_2_SeSO_3_, KSeCN, and Cu_2_Se.^66,67^ Accordingly, there is a persistent need for mild yet straightforward methods that employ stable OSe precursors compatible with a wide range of functional groups under diverse reaction conditions. Among these, diaryl diselenides are considered as versatile precursors for the construction of several multifunctional OSe libraries.^14,68,69^ For example, they can be readily converted to aryl selenide halides (ArSeX), which participate in different selenocyclization reactions of olefins and acetylenes to furnish structurally diverse selenaheterocycles. Moreover, they are versatile intermediates that can undergo conversion into various reactive Se species (*e.g.*, RSe^−^, RSe˙, and RSe^+^) and higher oxidation-state derivatives, including selenenic, seleninic, and selenonic acids.^65,70^ Additionally, their significant ease of handling and stability make diaryl diselenides ideal OSe reagents for the optimization of novel organic reactions. On the other hand, Schiff bases and *N*-amidic carboxylic acid derivatives (*e.g.*, maleanilic and succinanilic acids) are well recognized for their potent pharmacological properties, including anticancer and anti-inflammatory activities.^43,71–75^

Within this context, our research group has recently developed several libraries of OSe-based Schiff bases and *N*-amidic acids, which exhibited promising anticancer and anti-inflammatory properties.^76–79^ Building on these findings, we aimed to further expand our chemical library of OSe compounds by synthesizing novel Schiff bases and *N*-amidic acid derivatives. From a molecular hybridization perspective, such OSe-tethered scaffolds represent an attractive strategy to integrate multiple pharmacophores into a single framework, thereby improving the pharmacological significance and broadening the therapeutic relevance of these candidates.

Unlike classical ebselen derivatives or previously reported OSe–Schiff bases, the present compounds are designed as O–Se–tethered Schiff base–amide acid hybrids, in which a monoatomic selenium linker connects a redox-active aromatic Schiff base to a polar *N*-amidic carboxylic acid moiety within a single molecular framework. This hybrid scaffold represents a new structural class that integrates selenium-mediated redox modulation with hydrogen-bonding and ionizable functionalities, thereby distinguishing it from earlier OSe pharmacophores.

**Scheme 1:**
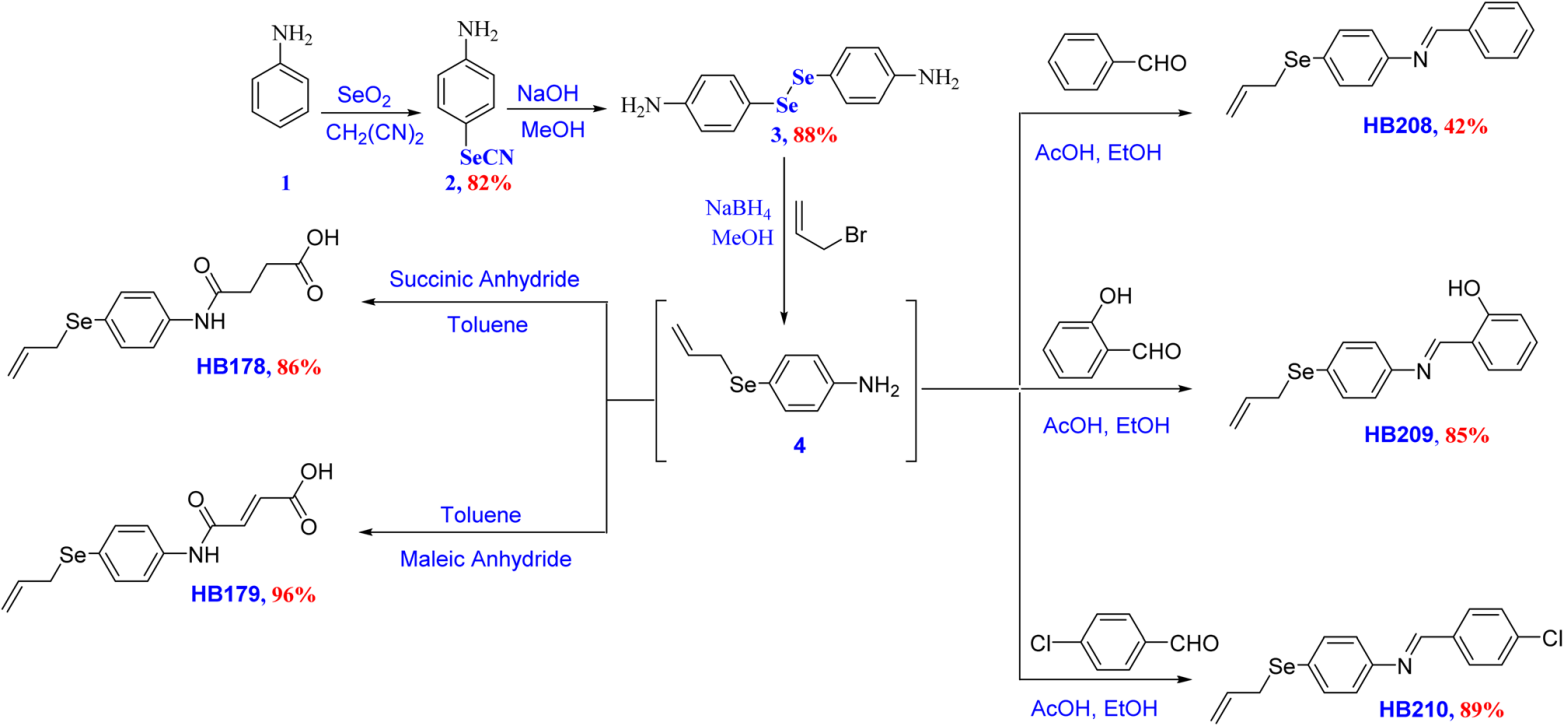
Synthesis of organoselenium derivatives (HB178, HB179, HB208, HB209, and HB210).

**Scheme 2:**
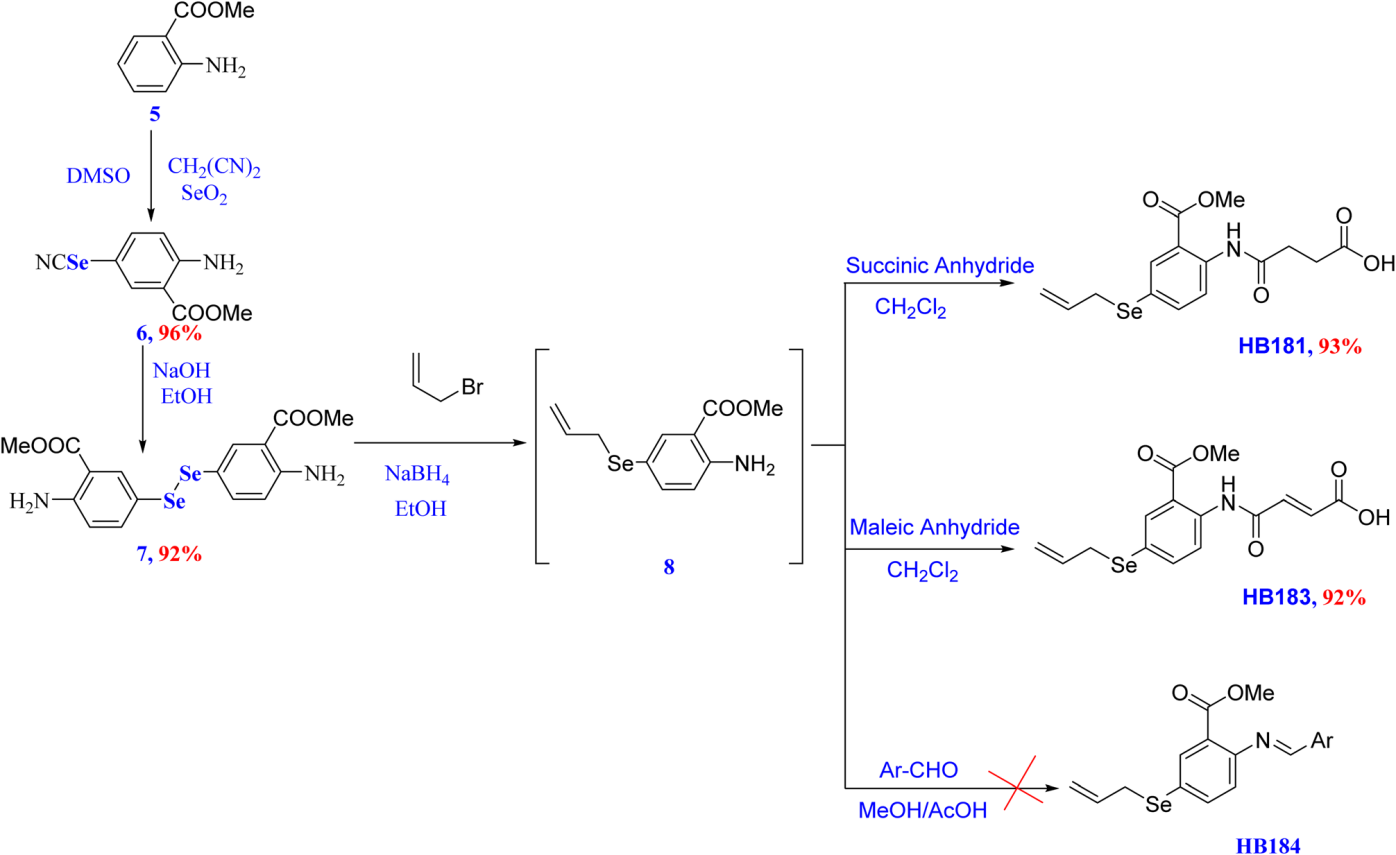
Synthesis of amidic acid derivatives (HB181 and HB183).

The key diaryl diselenide precursors 4,4′-diselanediyldianiline (3) and dimethyl 5,5′-diselanediylbis(2-aminobenzoate) (11) were obtained from the selenocyanation of aniline (1) and methyl 2-aminobenzoate (9) using malononitrile and SeO_2_ in DMSO to give the corresponding 4-selenocyanatoaniline (2) and methyl 2-amino-5-selenocyanatobenzoate (10) in 82% and 96% yields, respectively.^80–82^ Hydrolysis of selenocyates 2 and 10 using NaOH in methanol afforded the diaryl diselenide precursors 3 and 11 in 83% and 96% yields, respectively, as shown in Schemes 1 and 2. This route shows features that are promising for future manufacturing or process development.

The diaryl diselenide precursors 3 and 7 were converted into the corresponding 4-(allylselanyl)aniline (4) and methyl 5-(allylselanyl)-2-aminobenzoate (8) intermediates *via* reduction using a mixture of NaOH and NaBH_4_ in methanol, followed by nucleophilic substitution reaction at the allylic carbon of allyl bromide. From a mechanistic viewpoint, the transformation of diaryl diselenides 3 and 7 into the corresponding allylselanyl intermediates 4 and 8 proceeded *via* base-mediated reductive cleavage of the diselenide (Se–Se) bond using a mixture of NaBH_4_ and NaOH in ethanol. The latter strategy was proven to enhance nucleophilic substitution even under air and generated the corresponding arylsodium selenolate species (ArSeNa) *in situ*, which then underwent nucleophilic substitution at the activated allylic carbon of allyl bromide to furnish the allyselanyl intermediates 4 and 8. The resulting intermediates 4 and 8 were found to be air-sensitive, and therefore were directly engaged in the subsequent condensation and amidation steps without isolation to minimize decomposition and side reactions. Therefore, the reaction was carried out directly to the next step without prior isolation or purification in order to avoid undesirable side reactions.

The treatments of OSe allyl intermediate 4 with different aldehydes afforded the corresponding Schiff bases HB208, HB209, and HB210 in 42%, 85%, and 89% yields, respectively. On the other hand, our attempts to prepare the corresponding Schiff bases HB184 from the OSe allyl intermediate 8 were unsuccessful. The failure of our trials to obtain the anticipated Schiff base HB184 from the ortho-ester-substituted intermediate 8 is plausibly attributed to steric and electronic crowding around the amino (NH_2_) group imposed by the adjacent ester (COOMe) group, which is expected to disfavor efficient imine formation under the employed condensation conditions.

The FT-IR spectra of the OSe Schiff bases HB208, HB209, and HB210 showed characteristic bands around 1618–1575 cm^−1^, indicating the imine (C=N) formation (Table 1). Furthermore, the ^1^H NMR spectra displayed the diagnostic imine proton (CH=N) as a singlet at *δ* 8.5–8.9 ppm, together with consistent allyl signals (vinyl protons at *δ* 5.7–6.0 ppm and Se–CH_2_ at *δ* ∼4.9 ppm). In the ^13^C NMR spectra, deshielded carbons for the imine appeared at (*δ* 159–163 ppm) and allylic carbons at *δ* ∼30 ppm. OSe Schiff base 5 showed the phenolic OH group as a strongly deshielded singlet at *δ* 12.95 ppm. For the OSe Schiff base 6, the aromatic rings exhibited the well-resolved AA'BB′ splitting patterns consistent with para-substituted phenyl rings.

**Table 1:** Spectroscopic data for OSe derivatives (HB178, HB179, HB208, HB209, and HB210)

Compound no.	FT-IR (cm^−1^)	^1^H-NMR (*δ*, ppm)	^13^C-NMR (*δ*, ppm)
HB178	3275 (OH), 3174 (NH), 2932 (C–H, aromatic), 1690 (C=O), 1650 (C=O), 1527 (C=C), 1187, 913, 815	12.07 (s, 1H, OH), 9.94 (s, 1H, NH), 7.47 (d, *J* = 8.6 Hz, 2H, Ar–H), 7.35 (d, *J* = 8.6 Hz, 2H, Ar–H), 5.83 (ddt, *J* = 17.3, 9.9, 7.5 Hz, 1H, =CH_allyl_), 4.98–4.80 (m, 2H, =CH_2_), 3.53–3.43 (m, 2H, CH_2_), 2.55–2.43 (d, 4H, 2CH_2_)	174.18, 170.54, 138.95, 135.10, 134.00, 122.70, 119.93, 117.16, 31.47, 30.52, 29.17
HB179	3275 (OH), 3186 (NH), 3089 (C–H, aromatic), 1696 (C=O), 1626 (C=O), 1526 (C=C), 1391, 1177, 819	13.00 (s, 1H, OH), 10.36 (s, 1H, NH), 7.52 (d, *J* = 8.6 Hz, 2H, Ar–H), 7.39 (d, *J* = 8.6 Hz, 2H, Ar–H), 6.42 (d, *J* = 12.1 Hz, 1H, CH=), 6.28–6.23 (m, 1H, =CH), 5.84 (ddt, *J* = 17.3, 9.9, 7.5 Hz, 1H, =CH_allyl_), 4.90 (ddd, *J* = 13.4, 11.2, 1.3 Hz, 2H, =CH_2_), 3.61–3.44 (m, 2H, CH_2_)	167.21, 163.65, 138.11, 135.05, 133.76, 132.12, 130.71, 124.08, 120.51, 117.29, 30.38
HB208	2976 (C–H, aromatic), 1575 (C=C), 1449, 914, 811	8.58 (s, 1H, =CH), 7.87 (t, *J* = 6.5 Hz, 2H, Ar–H), 7.50–7.43 (m, 4H, Ar–H), 7.15 (t, *J* = 9.0 Hz, 2H, Ar–H), 7.10 (d, *J* = 8.2 Hz, 1H, Ar–H), 6.44 (d, *J* = 8.2 Hz, 1H, =CH), 5.96–5.73 (m, 2H, =CH_2_), 3.58 (d, *J* = 7.4 Hz, 2H, CH_2_)	161.09, 150.54, 135.01, 133.59, 129.57, 129.24, 129.10, 122.25, 117.45, 116.53, 114.97, 30.15
HB209	2976 (C–H, aromatic), 1575 (C=C), 1449, 914, 811	12.95 (s, 1H, OH), 8.92 (s, 1H=CH), 7.60 (d, *J* = 7.6 Hz, 1H, Ar–H), 7.50 (d, *J* = 8.2 Hz, 2H, Ar–H), 7.37 (t, *J* = 7.5 Hz, 1H, Ar–H), 7.31 (d, *J* = 8.2 Hz, 1H, Ar–H), 6.97–6.88 (m, 2H, Ar–H), 5.88 (dq, *J* = 9.6, 7.5 Hz, 1H, =CH), 4.97 (dd, *J* = 46.9, 13.4 Hz, 2H, =CH_2_), 3.62 (d, *J* = 7.4 Hz, 2H, CH_2_)	163.67, 160.67, 147.16, 134.95, 133.73, 133.46, 132.94, 128.71, 122.52, 119.74, 119.58, 117.56, 117.00, 30.01
HB210	2979 (C–H, aromatic), 1618 (C=C), 1487, 1402, 825	8.61 (s, 1H, =CH), 7.91 (d, *J* = 8.5 Hz, 2H, Ar–H), 7.55 (d, *J* = 8.4 Hz, 2H, Ar–H), 7.47 (d, *J* = 8.4 Hz, 2H, Ar–H), 7.18 (d, *J* = 8.3 Hz, 2H, Ar–H), 5.87 (ddt, *J* = 17.3, 9.9, 7.5 Hz, 1H, =CH), 4.95 (dd, *J* = 41.7, 13.5 Hz, 2H, =CH_2_), 3.64–3.53 (m, 2H, CH_2_)	159.84, 150.19, 136.49, 135.26, 135.02, 133.52, 130.70, 129.39, 127.68, 122.31, 117.46, 30.11

The OSe and *N*-amidic acids HB178, HB179, HB181, and HB183 were prepared in 86%, 96%, 93%, and 92% from diselenide precursors 4 and 8 *via* the reaction with maleic or succinic acids in toluene or CH_2_Cl_2,_ as shown in Schemes 1 and 2, respectively.

The FT-IR spectra of the OSe and *N*-amidic acids HB178, HB179, HB181, and HB183 showed both broad OH and NH stretches (∼3275–3160 cm^−1^) and two distinct C=O (1685, 1579 cm^−1^) bands, supporting the amidic acid patterns (Table 2). In the ^1^H NMR, the strongly deshielded OH and NH protons were observed around *δ* ∼12–13 ppm and *δ* ∼9.94–10.71 ppm, respectively. Also, the allylselanyl fragments were observed at *δ* ∼5.8–5.9 ppm (=CH), *δ* ∼4.9–5.0 ppm (=CH_2_), and *δ* ∼3.5–3.6 ppm (Se–CH_2_). The OSe *N*-mealanilic acids HB179 and HB183 (α,β-unsaturated derivatives) displayed additional olefinic protons at *δ* 6.2–6.5 and a lower-frequency conjugated carbonyls (*δ* ∼167.21–163.65 ppm) in the ^13^CNMR. Additionally, the OSe and *N*-amidic acids HB181 and HB183 also featured a methyl (COOMe) singlet signal at *δ* ∼3.8 ppm, supporting the presence of the ester substituent.

**Table 2:** Spectroscopic data for OSe amidic acid derivatives (HB181 and HB183)

Compound no.	FT-IR (cm^−1^)	^1^H-NMR (*δ*, ppm)	^13^C-NMR (*δ*, ppm)
HB181	3259 (OH), 3156 (NH), 2952 (C–H, aromatic), 1685 (C=O), 1579 (C=O), 1512 (C=C), 1250, 909, 789	12.13 (s, 1H, OH), 10.51 (d, *J* = 8.8 Hz, 1H, NH), 8.12 (d, *J* = 8.8 Hz, 1H, Ar–H), 7.91 (t, *J* = 2.7 Hz, 1H, Ar–H), 7.65 (dd, *J* = 8.6, 1.6 Hz, 1H, Ar–H), 5.88–5.78 (m, 1H, =CH_allyl_), 4.94–4.84 (ddd, *J* = 13.4, 11.2, 1.3 Hz, 2H, =CH_2_), 3.81 (s, 3H, CH_3_), 3.56 (d, *J* = 7.5 Hz, 2H, CH_2_), 2.57 (d, *J* = 6.5 Hz, 2H, CH_2_), 2.49 (t, *J* = 5.3 Hz, 2H, CH_2_)	173.95, 170.72, 167.41, 139.03, 138.78, 135.08, 1349.89, 134.82, 123.22, 122.08, 118.73, 117.56, 32.21, 30.61, 29.13, 14.37
HB183	3360 (OH), 3165 (NH), 3006 (C–H, aromatic), 1710 (C=O), 1683 (C=O), 1591 (C=C), 1235, 833, 787	12.93 (s, 1H, OH), 10.71 (s, 1H, NH), 8.12 (d, *J* = 8.6 Hz, 1H, Ar–H), 7.92 (d, *J* = 2.1 Hz, 1H, 1H, Ar–H), 7.69 (dd, *J* = 8.6, 2.1 Hz, 1H, 1H, Ar–H), 6.52 (d, *J* = 12.0 Hz, 1H, =CH), 6.32–6.26 (d, *J* = 12.0 Hz, 1H, CH=), 5.84 (ddt, *J* = 17.4, 9.9, 7.5 Hz, 1H, =CH_allyl_), 5.02–4.83 (ddd, *J* = 13.4, 11.2, 1.3 Hz, 2H, =CH_2_), 3.80 (s, 3H, CH_3_), 3.63–3.53 (m, 2H, CH_2_)	167.22, 167.01, 163.89, 138.50, 138.34, 138.17, 134.83, 134.80, 132.73, 130.44, 124.37, 119.42, 117.67, 30.50, 14.33

### 2.2. Biological assays

#### 2.2.1. Percentage of cellular growth inhibition (GI) against a variety of cancerous and normal cell lines

Schiff bases-tethered OSe analogues were tested against eight cancer cells to determine their growth inhibition (GI) efficacy which include: the undifferentiated carcinoma of the parotid gland (HN9), hepatocellular carcinoma (HuH7, HEPG2), squamous cell carcinoma (FaDu), human breast cancer (MCF7), human adenocarcinoma (A549), colorectal carcinomas (HCT_116_), human melanoma (A375). Additionally, the GI% of novel Schiff bases-tethered OSe analogues was assessed using human skin fibroblasts (HSF) and the olfactory ensheathing cell line (OEC) normal cell lines. Table 3 displays the results of the GI activity for the evaluated analogues in comparison to doxorubicin (DOX), the reference anticancer drug.

**Table 3:** Growth inhibition (GI) percentage^a^^b^ of novel OSe analogues against eight cancer cell lines and two normal ones

Cell line/Comp	HB178	HB179	HB181	HB183	HB208	HB209	HB210	DOX	Average
HN9	86.39 ± 0.95	84.73 ± 5.32	64.97 ± 2.34	83.00 ± 3.27	65.52 ± 4.83	85.64 ± 2.80	83.00 ± 5.03	51.73 ± 2.57	75.62
HuH7	66.96 ± 2.47	73.01 ± 5.08	81.14 ± 6.00	72.06 ± 6.85	80.30 ± 5.37	83.94 ± 1.33	88.03 ± 2.46	68.00 ± 1.82	76.68
FaDu	72.57 ± 2.39	72.89 ± 3.44	71.64 ± 7.23	77.18 ± 3.80	62.81 ± 6.22	69.14 ± 7.27	74.20 ± 9.34	79.34 ± 4.65	72.47
MCF7	75.01 ± 3.55	76.89 ± 1.58	60.33 ± 1.27	80.01 ± 1.86	62.00 ± 6.55	69.12 ± 5.76	76.33 ± 0.95	58.65 ± 3.54	69.80
HEPG2	80.20 ± 5.82	77.85 ± 8.12	74.19 ± 8.49	80.69 ± 3.40	76.87 ± 3.43	71.54 ± 4.78	78.77 ± 4.15	55.62 ± 3.13	74.47
A549	75.87 ± 6.93	48.79 ± 1.28	72.66 ± 8.73	71.55 ± 2.49	68.04 ± 8.08	69.43 ± 9.42	75.24 ± 6.98	39.14 ± 6.05	65.09
HCT_116_	64.92 ± 3.47	79.87 ± 5.27	48.31 ± 6.27	79.51 ± 4.34	69.19 ± 5.18	80.33 ± 5.88	73.88 ± 7.73	64.36 ± 4.20	70.05
A375	80.30 ± 5.17	87.90 ± 1.88	77.70 ± 7.63	82.01 ± 2.31	88.46 ± 3.74	81.55 ± 5.87	83.68 ± 2.37	78.26 ± 5.72	82.48
Average	75.28	75.24	68.87	78.25	71.65	76.34	79.14	61.89	
OEC	62.09 ± 6.72	67.43 ± 3.60	49.91 ± 8.15	53.90 ± 13.46	48.25 ± 16.40	58.46 ± 7.03	51.36 ± 18.61	31.96 ± 12.09	45.74
HSF	45.07 ± 14.99	36.92 ± 6.13	32.18 ± 7.38	42.27 ± 18.48	39.85 ± 7.99	50.68 ± 16.69	45.08 ± 16.91	29.57 ± 7.74	40.20

a Data are presented as mean ± SD (*n* = 3). The results are performed using GraphPad InStat, version 8.02.

b Statistical significance between cancer and normal cells is indicated (*p < 0.05, *p < 0.01).

#### 2.2.2. Structure–activity relationships (SAR)

From the previous Table 3, it was noticed that HB183, HB210, and HB209 have promising average GI percentages of (78.25%, 79.14%, and 76.34%), respectively, in comparison with 61.89% for the reference DOX as a growth inhibitor. They are Schiff bases that are OSe-tethered and have a monoatomic selenium linker.

A closer examination of how these compounds affect the various cell lines shows that HB183 has a stronger GI effect in comparison with DOX towards HEPG2, MCF7, HCT_116_, FaDu, and A549 cell lines (80.69%, 80.01%, 79.51%, 77.18%, and 71.55%, respectively). HB210 has inhibited the growth of cancer cells including HuH7, HEPG2, MCF7, A549, and HCT_116_ with values of (88.03%, 78.77%, 76.33%, 75.24%, and 73.88%), respectively. The GI% values for HB209 are (83.94, 80.33, 71.54, 69.43, and 69.12), against HuH7, HCT_116,_ HEPG2, A549, and MCF7 cell lines, respectively. The GI percentages of HB183, HB210, and HB2019 against the mentioned cell lines were found to be better than DOX itself.

**Fig. 4:**
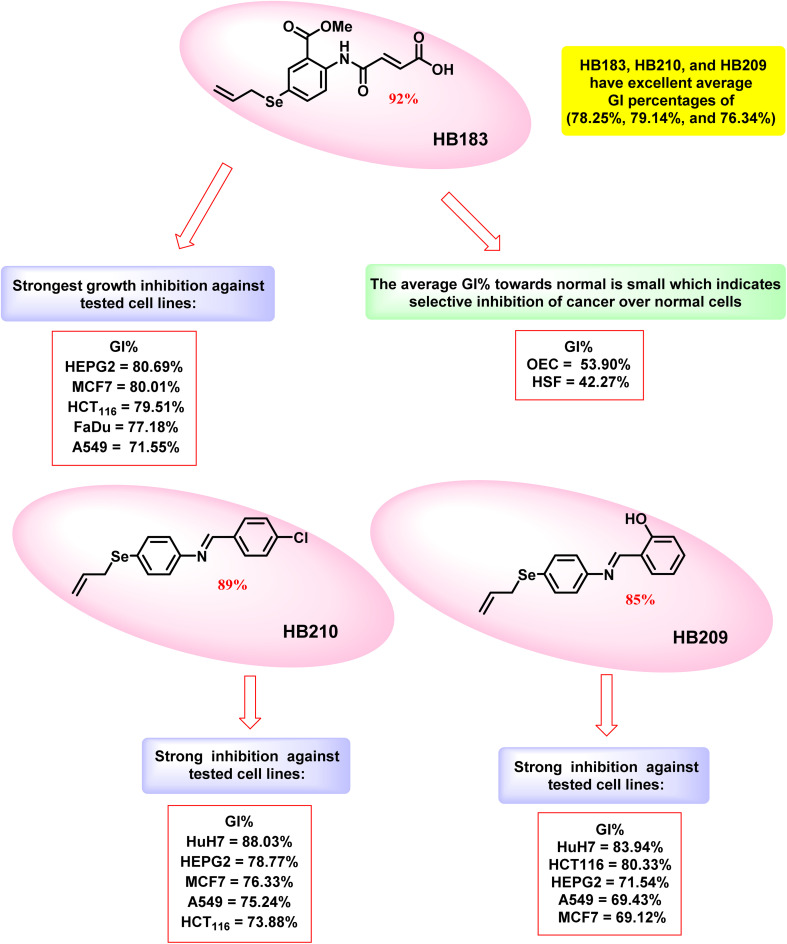
Structure–activity relationship of compounds HB183, HB210, and HB209.

Remarkably, the normal cells OEC and HSF were the least vulnerable to the GI effect of the newly evaluated compounds. The average GI% towards those cells was 45.74% and 40.20%, respectively. When it comes to normal cells, HB183 exhibited a small degree of GI, which is 53.90% and 42.27%, towards OEC and HSF, respectively. Although these results suggest some degree of selectivity for cancer cells over healthy cells, the relatively high GI% in normal cells indicates that this selectivity is moderate rather than absolute. These findings highlight the importance of careful evaluation of potential cytotoxicity in normal cells and indicate that further optimization may be necessary to enhance the therapeutic window of these compounds (Fig. 4).

Statistical analysis confirmed that GI% in cancer cells was significantly higher than in normal OEC and HSF cells (*p* < 0.05). However, GI% in normal cells remained substantial (∼40–55%), indicating that selectivity is moderate rather than absolute.

#### 2.2.3. Assessment of cytotoxic inhibitory concentration 50 (IC_50_) against HEPG2, A549, HCT_116_, HuH7, FaDu, and MCF7 cell lines

**Fig. 5:**
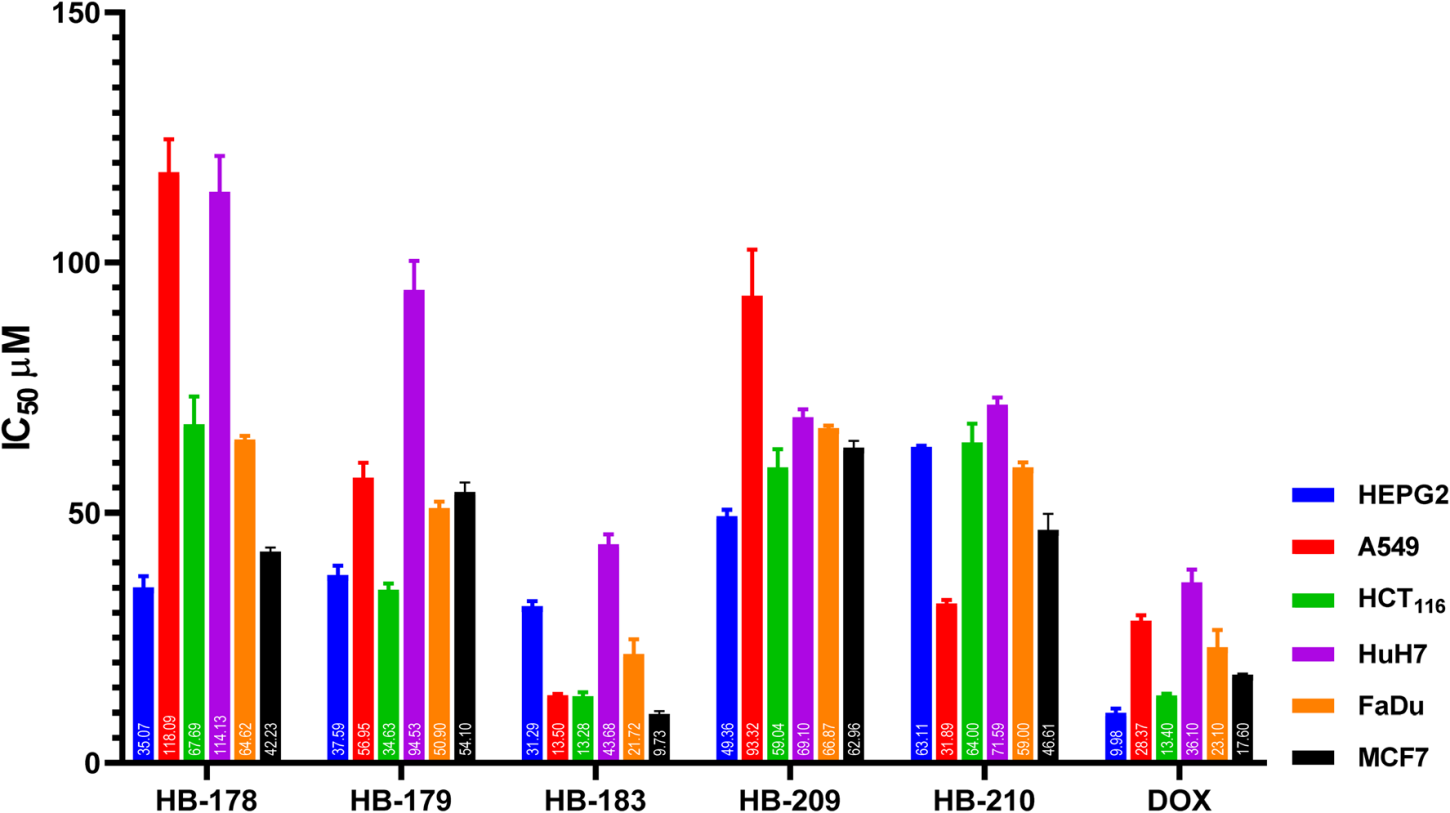
Evaluation of the investigated compounds HB178, HB179, HB183, HB209, and HB210 cytotoxic inhibitory concentration 50 (IC_50_) against the cancer cell lines; HEPG2, A549, HCT_116_, HuH7, FaDu, and MCF7 after 48 h.Graphs were performed using GraphPad InStat, version 8.02. The results are expressed as the mean ± SD of 3 separate experiments performed in 3 replicates.

HB178, HB179, HB183, HB209, and HB210 were tested against the cancer cells with the highest GI% to determine their IC_50_. The IC_50_ was determined against HEPG2, A549, HCT_116_, HuH7, FaDu, and MCF7 by the SRB assay^83^ (Fig. 5). Various concentrations of the investigated compounds (12.5, 25, 50, and 100 µM equivalents) were examined using the chosen cancer cells.

HB183 exhibited the lowest IC_50_ values of 31.28, 13.50, 13.28, 43.69, 21.70, and 9.72 µM compared to those of DOX, which were 9.97, 28.38, 13.40, 36.11, 23.09, and 17.59 µM against HEPG2, A549, HCT116, HuH7, FaDu, and MCF7 cancer cell lines, respectively. Notably, HB183 demonstrated marked potency towards the MCF7 cell line, supporting its potential anticancer effect.

When compared with representative OSe compounds reported in the literature, HB183 exhibits cytotoxicity in a similar micromolar range in several cancer cell lines. Preliminary chemical stability studies under assay conditions indicated that HB183 and related compounds remained largely intact over the incubation period, suggesting that the observed cytotoxicity primarily reflects the parent compound rather than degradation products. For example, ebselen has been reported with an IC_50_ of approximately ∼12.5 µM in A549 lung carcinoma cells, and ethaselen, an ebselen analogue targeting thioredoxin reductase, shows IC_50_ values of ∼15–18 µM in prostate cancer cells.^84,85^ More optimized OSe derivatives, such as NSAID–ebselen hybrids, have demonstrated lower IC_50_ values (∼1.5–3 µM) against MCF-7 cells, indicating that scaffold modifications can enhance potency.^86^ These comparisons suggest that HB183's IC_50_ values, particularly against MCF-7 (∼9.7 µM), are promising, albeit further modifications could improve both anticancer efficacy and therapeutic margin. In addition to BCL-2 inhibition, organoselenium compounds, including HB183, may exert cytotoxic effects *via* alternative redox-mediated mechanisms, such as reactive oxygen species (ROS) generation, thiol oxidation, and modulation of redox-sensitive signaling pathways, which likely contribute to the observed antiproliferative activity.^87^

Furthermore, OSe compounds, including HB183, may exert cytotoxic effects *via* alternative redox-mediated mechanisms, such as ROS generation, thiol oxidation, and modulation of redox-sensitive signaling pathways, in addition to BCL-2 inhibition. These mechanisms may contribute to the observed antiproliferative activity, particularly in cell lines showing high sensitivity.^88,89^

Notably, a detailed representation of the IC_50_ of each tested cell line with the evaluated compound is represented in the SI.

#### 2.2.4. Protein expression of genes associated with apoptosis

Using a protein expression study for genes linked to apoptosis, the apoptotic ability of HB183 analogue on the most susceptible MCF7 cancer cells was investigated. The goal was to find any changes in protein expression and learn more about the molecular processes that underlie the apoptotic effects brought on by the examined candidate.

**Fig. 6:**
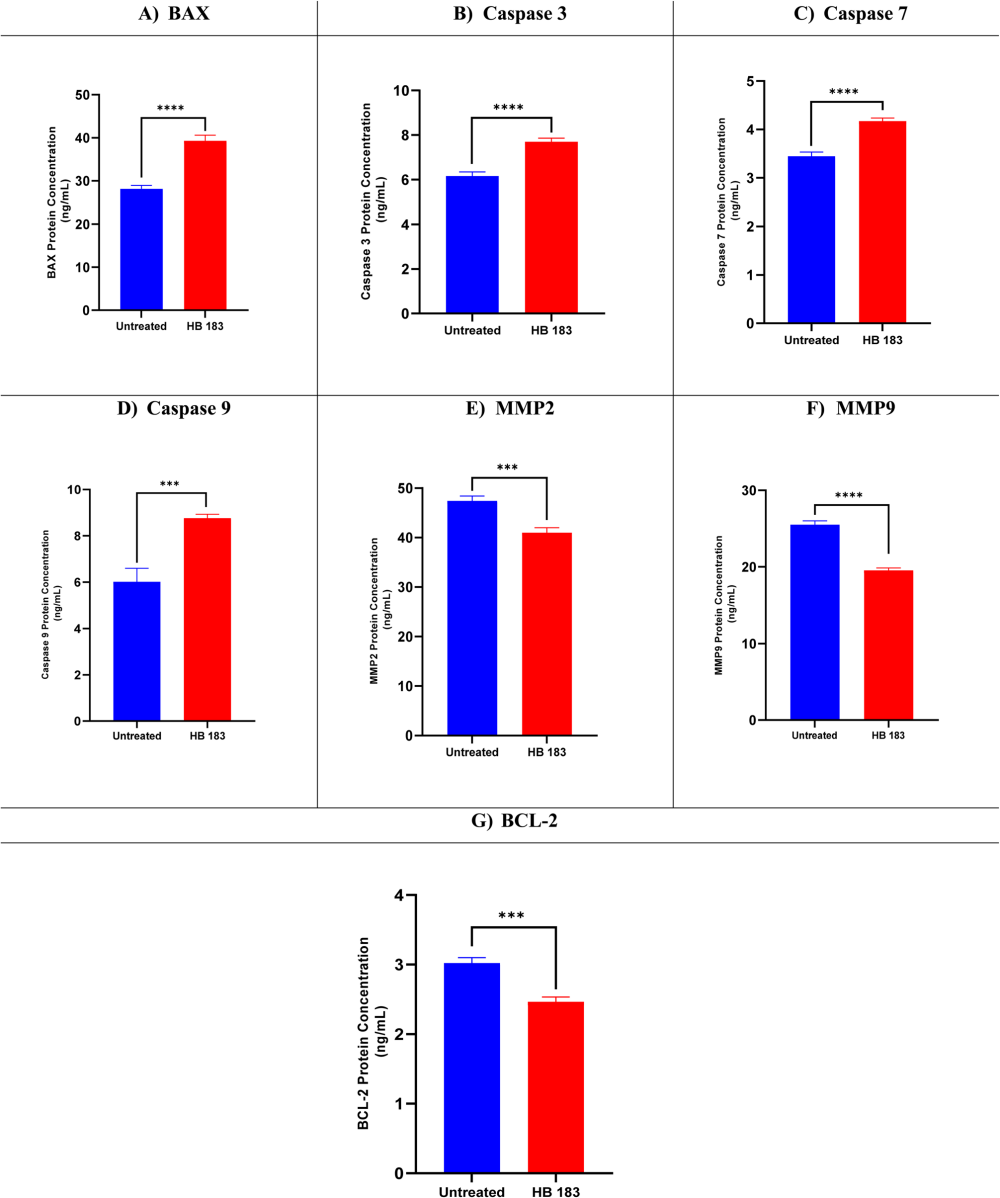
BAX (A), caspases 3 (B), 7 (C), and 9 (D), MMP2 (E), MMP9 (F), and BCL-2 (G) protein expression levels for compound HB183 in the treated and untreated MCF7 cancer cell line.

Protein expressions for BAX, caspases 3, 7, and 9, MMP2, MMP9, and BCL-2 were evaluated in both compound HB183-treated cells and the untreated negative control cells. It is interesting to note that compound HB183 treatment increased apoptotic protein expression. Therefore, BAX, caspases 3, 7, and 9 were upregulated by (1.39, 1.18, 1.20, 1.45)-fold change, respectively. Besides, the anti-apoptotic proteins (MMP2, MMP9, and BCL-2) were downregulated by (1.15, 1.30, and 1.22)-fold-change, respectively, indicating the apoptotic potential as seen in Fig. 6.

Collectively, HB183 induced a coordinated modulation of multiple apoptosis-related proteins, including upregulation of BAX and caspases and downregulation of BCL-2 and mitochondrial membrane proteins, indicating activation of the intrinsic mitochondrial apoptotic pathway. This mode of action is consistent with previous reports showing that organoselenium compounds downregulate anti-apoptotic BCL-2 while upregulating pro-apoptotic Bax and activating caspases in cancer cells.^90^ Importantly, while BCL-2 levels were reduced, this effect likely reflects a downstream component of mitochondrial apoptosis rather than exclusive target engagement, consistent with the well-established pleiotropic redox-modulating nature of OSe compounds, which can influence glutathione and ROS pathways in addition to apoptosis signaling.^91^ Additionally, organoselenium agents such as ebselen are widely used to probe cellular redox biology through interactions with thiol redox systems, supporting the interpretation that multiple redox pathways contribute to the observed phenotype.^92^ Similar multi-targeted modulation of apoptotic proteins, including BAX, BCL-2, caspases, and metalloproteinases, has been reported for other OSe analogues.^72^

#### 2.2.5. Assessment of cell cycle arrest for HB183 in the MCF7 breast cancer cell line

**Fig. 7:**
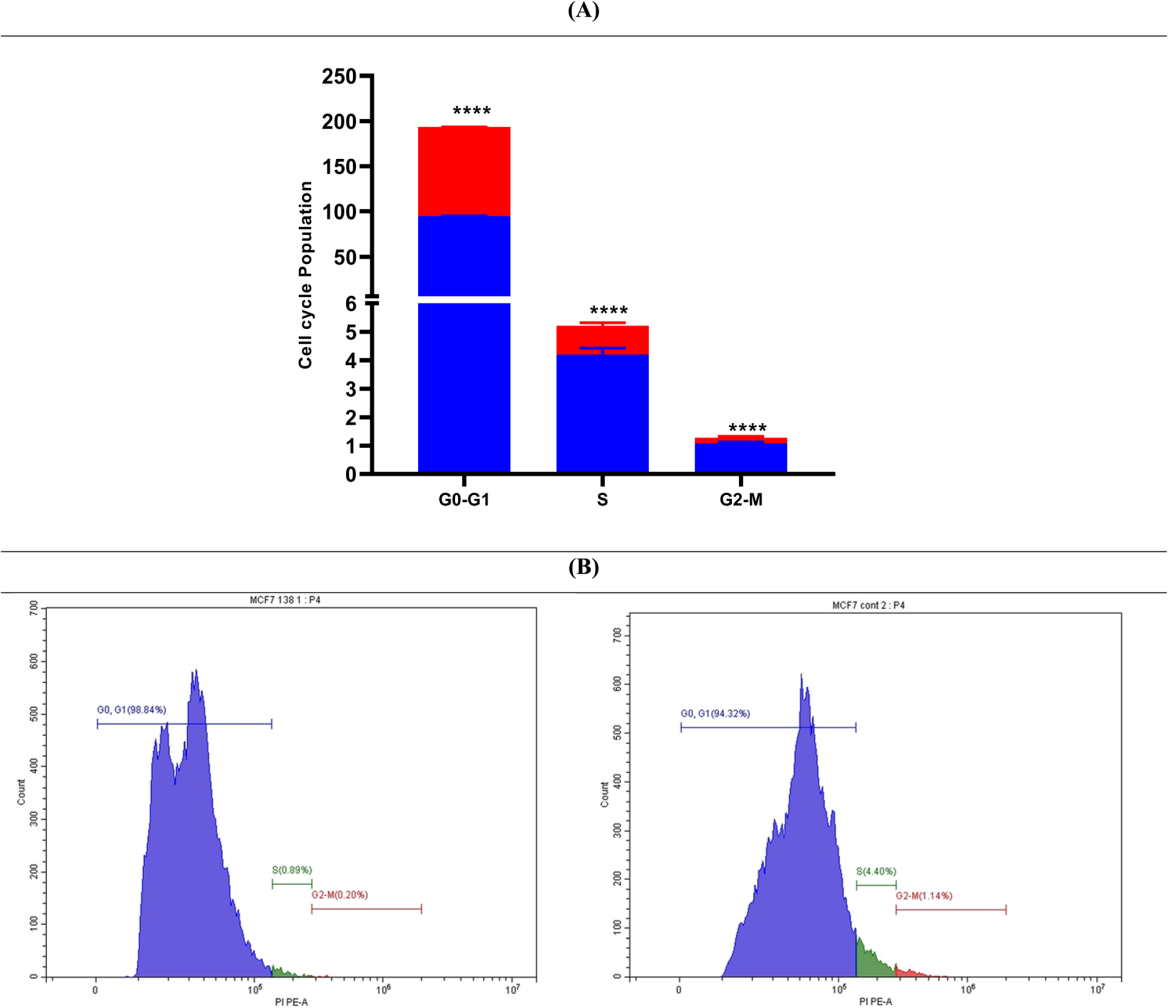
(A) Cell cycle analysis of treated & untreated MCF7 cells with compound HB183. (B) Histograms of MCF7 treated with compound HB183 (left side) *vs.* control (right side). The experiments were performed in three independent replicates as mean ± SD. Statistical significance was evaluated using one-way ANOVA followed by Tukey's multiple comparison post hoc test. *Significance threshold from untreated cells of *p* < 0.05 was applied.

Since HB183 showed strong anticancer potential against the MCF7 with an IC_50_ value of 9.72 µM, it was put through a flow cytometric experiment to identify the cell cycle stage that it arrested in the MCF7 breast cancer cell line.^93,94^ Treatment with HB183 led to a marked increase in the sub-G1 (pre-G1) population from 94.32% to 98.84%, accompanied by a decrease in the S-phase fraction from 4.40% to 0.89% and a reduction in the G2/M fraction from 1.14% to 0.20% (Fig. 7). The increase in the sub-G1 population reflects enhanced DNA fragmentation and apoptotic cell death rather than the accumulation of viable cells in a specific cell-cycle phase. These results indicate that HB183 induces apoptosis in MCF7 cells, which is consistent with its potent cytotoxic activity. Cell-cycle analysis was performed in triplicate, and representative histograms are provided in the SI.

### *2.3*. *In silico* studies

#### 2.3.1. Molecular docking

A molecular docking process for the lead analogue (HB183) towards the BCL-2 target, as a crucial one in the pathway of apoptosis induction, was performed. This was done to propose HB183 as a BCL-2 inhibitor. The target receptor was selected from the Protein Data Bank (ID: 4IEH), and its large *N*-heteroaryl sulfonamide (co-crystal inhibitor) was inserted as a positive reference.

Analogue HB183 recorded a binding score of −6.37 kcal mol^−1^ at a root mean square deviation (RMSD) of 1.70 Å. On the other side, the docked co-crystal ligand of the BCL-2 reached a binding score of −8.63 kcal mol^−1^ at an RMSD of 1.68 Å. The lower score of analogue HB183 may be attributed to its smaller size compared to the large N-heteroaryl sulfonamide (co-crystal inhibitor), which results in a smaller number of hydrogen and hydrophobic interactions.

**Fig. 8:**
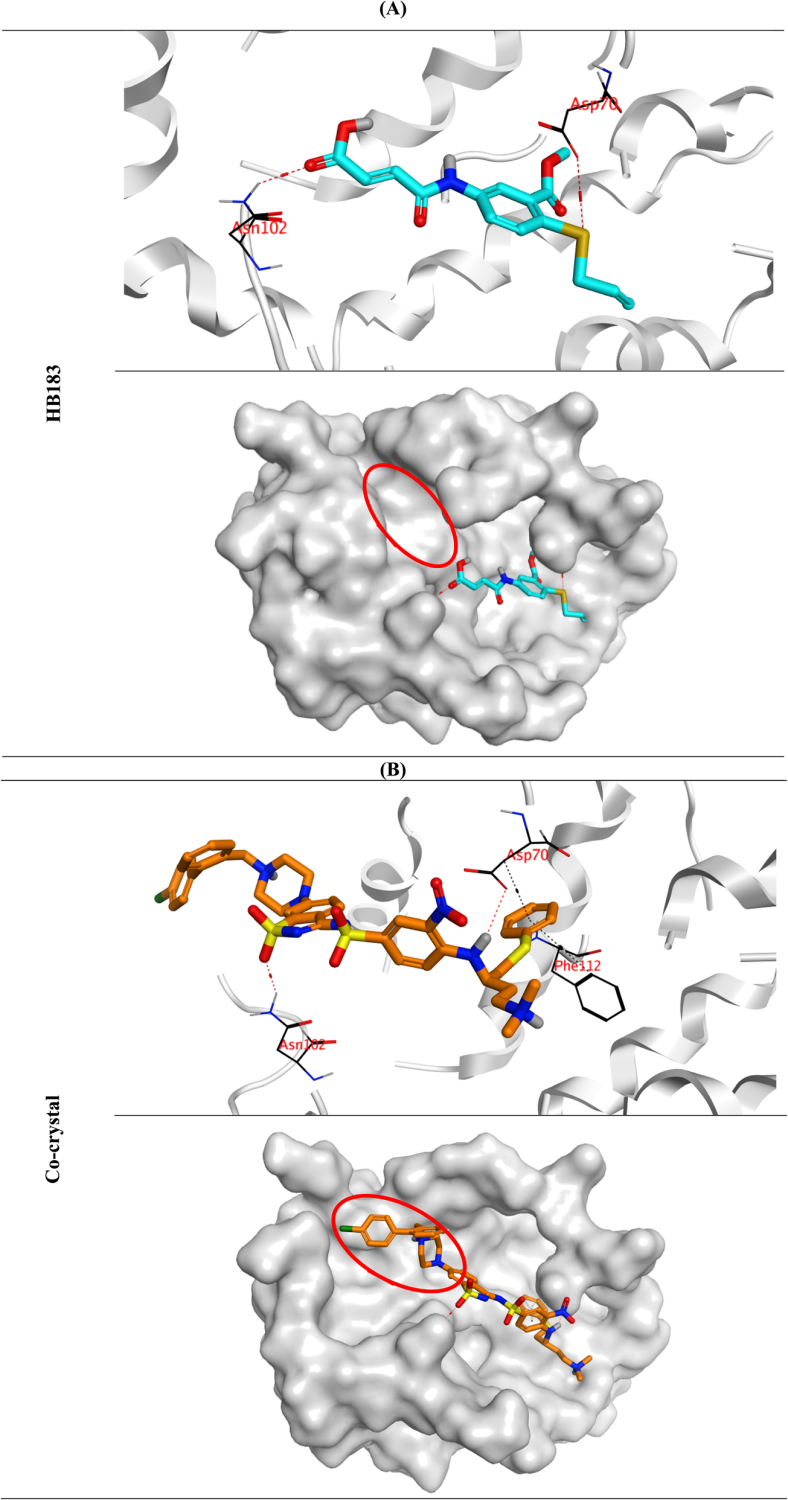
3D binding interactions and positioning for analogue HB183 (A) within the active pocket of BCL-2 receptor (PDB ID: 4IEH), with respect to the co-crystal ligand (B).

The small analogue (HB183) got stabilized inside the active pocket of the BCL-2 by forming two hydrogen bonds with (Asp70 and Asn102), the two crucial amino acids. Moreover, the docked co-crystal inhibitor of the BCL-2 showed three hydrogen bonds with (Asp70, Asn102, and Glu95). Besides, it showed two pi-hydrogen bonds with Asp70 and Phe112 (Fig. 8).

Notably, the role of the acidic nature of the sulfonamide moiety of the co-crystal inhibitor is important for its potency, solubility, and clearance as well. Additionally, the distance between the NH group (attached to Asp70) and the sulfone oxygen (attached to Asn102) was observed to be eight atoms. Surprisingly, the binding mode of the small analogue (HB183) showed the same features as the co-crystal inhibitor of the BCL-2. Where the distance between the Se atom (attached to Asp70) and the carbonyl oxygen (attached to Asn102) was observed to be eight atoms as well. Moreover, the acidic nature of the OSe analogue (HB183) with a free COOH group simulates the acidic nature of the sulfonamide moiety of the co-crystal inhibitor.

Based on the aforementioned findings, the small analogue (HB183) is greatly recommended to be a strong BCL-2 inhibitor with the potential for further optimization through an extension approach to extend to the extra vacant pocket of the BCL-2 active site.

#### 2.3.2. Molecular dynamics simulation

The stability of the HB183 inside the cavity of the BCL-2 was assessed using molecular dynamics simulation, and the complex was subject to 500 ns of simulation time.

**Fig. 9:**
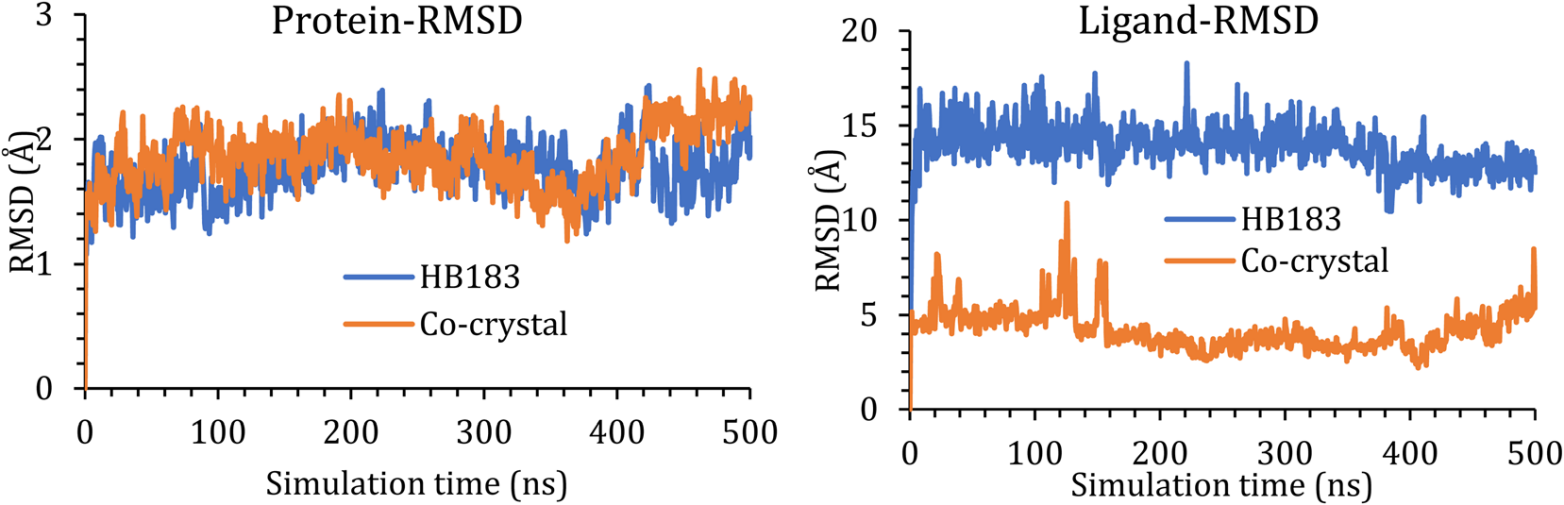
(Left side) RMSD of the ligand–protein complex backbone during the simulation time; (Right side) RMSD of the ligand atoms with respect to their initial position inside the cavity of the BCL-2.

The RMSD of the protein Cα atoms was monitored with respect to their initial position and represented as a function of simulation time (Fig. 9a). HB183 did not affect the conformation of the human BCL-2, and its backbone described an RMSD of less than 2.50 Å. The co-crystal ligand, Fig. 9a, showed a similar RMSD.

**Fig. 10:**
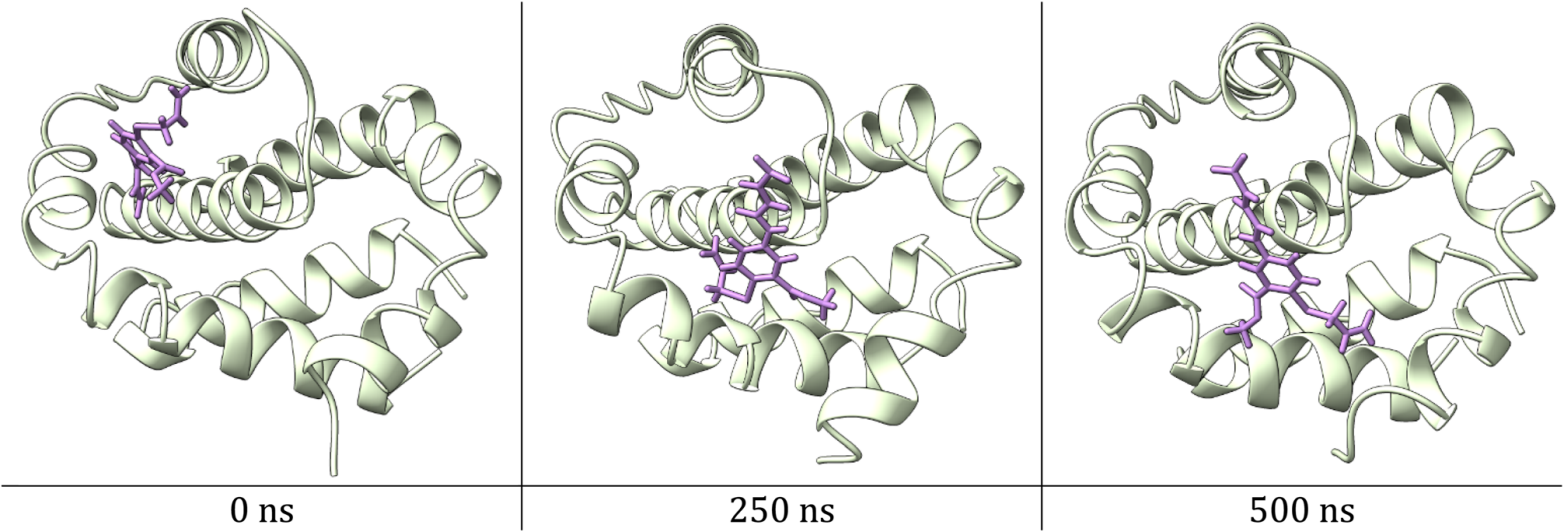
A snapshot of ligand movement inside the active site cavity at 0, 250, and 500 ns of simulation time.

In addition, the RMSD for ligands was also monitored with respect to their initial position inside the active pocket of the protein and plotted as a function of simulation time, Fig. 9b. HB183 shows an RMSD of around 15.00 Å, which is quite large for ligand movement. However, the movement is entirely justified, as the BCL-2 active site is a quite large cavity. Hence, the docking pose moved as the simulation started and settled down on the other side of the cavity, as shown in Fig. 10. The movement of the ligand will be discussed in detail in the next section. Besides, the co-crystal structure recorded an RMSD of around 5.00 Å throughout the simulation time.

**Fig. 11:**
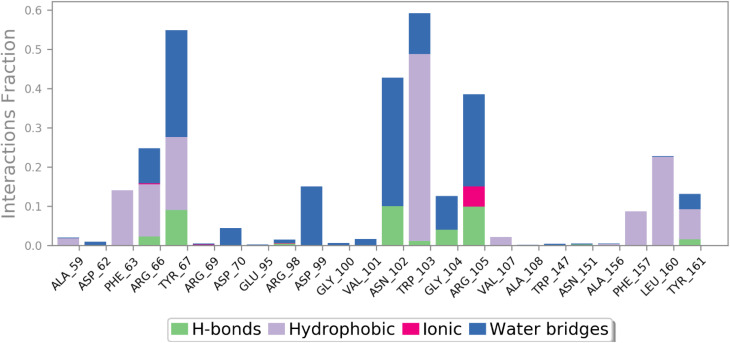
The interaction between HB183 and the active site residue during the simulation time is presented as a percentage.

The interaction of HB183 with the residue of the active site cavity was analyzed using the simulation interactions diagram panel of Maestro software. First, the interaction was reported as precent, presenting the interaction of the compound with the amino acid within the active pocket (Fig. 11). As can be seen, HB183 was able to form multiple interactions toward Trp103 (60%), Tyr76 (55%), Asn102, and Arg105.

**Fig. 12:**
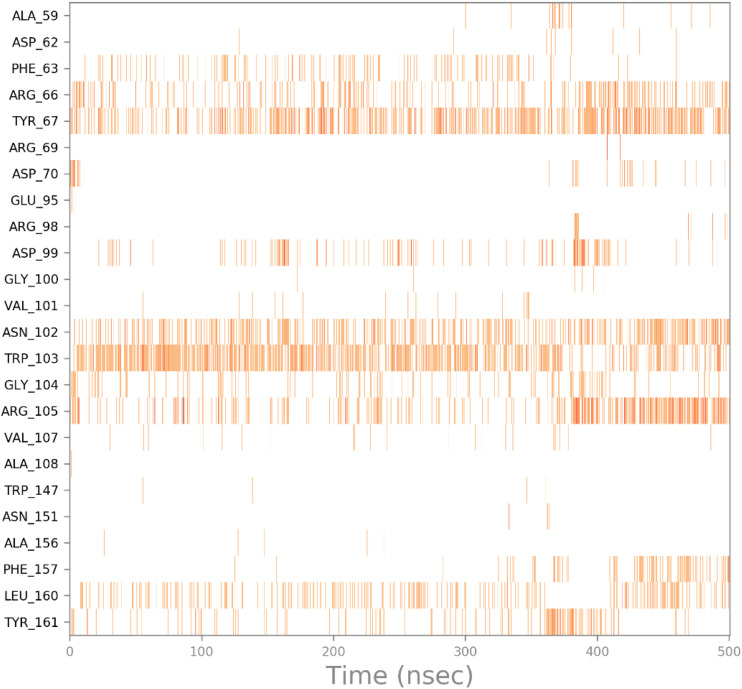
Heatmap of the interaction between HB183 and the active site residues as a function of time.

Next, the interactions timeline was plotted as a function of simulation time, where the intensity of the color indicates the number of interactions formed, as shown in Fig. 12.

**Fig. 13:**
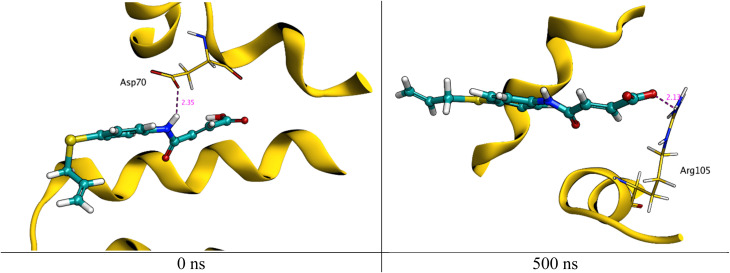
Interaction of HB183 with active site residue at 0 and 500 ns of simulation time.

As can be seen in Fig. 12, HB183 loses its interaction with Asp70 at around 5.00 ns of simulation time, which leads to the compound moving within the cavity of the active site to the other side to connect with Arg105, as shown in Fig. 13.

#### 2.3.3. MM-GBSA calculations

The MM-GBSA calculations were carried out for HB183 using the thermal_mmgbsa.py Python script of Schrödinger, along with the co-crystal ligand, and are reported in Table 4.

**Table 4:** MM-GBSA energies for HB183 and co-crystal complexes, reported in kcal mol^−1^

Complex	Δ*G* binding	Coulomb	Covalent	H-bond	Lipo	Bind packing	Solv_GB	vdW
HB183	−107.02	−8.13	2.67	−3.39	−32.77	−2.32	22.46	−85.60
Co-crystal	−122.36	0.90	3.07	−4.30	−37.49	−2.90	13.52	−95.246

The analysis of the binding components reveals several important insights:

(a) Electrostatic interactions: the strong negative coulombic energy highlights the importance of ionic and polar interactions between HB183 and its target. This implies that charged or polar residues within the binding site are effectively interacting with the ligand, enhancing binding strength.

(b) Hydrogen bonds: the contribution from hydrogen bonds (−39.27 kcal mol^−1^) is notably high, indicating that HB183 forms considerable hydrogen bonds with the protein. This reinforces the ligand's position within the active site and further stabilizes the complex.

(c) van der Waals and packing contributions: The van der Waals interactions (−85.46 kcal mol^−1^) and the overall packing energies (−1.90 kcal mol^−1^) suggest that there is a favorable fit of HB183 within the binding pocket. The geometry and sterics of HB183 align well with the active site, minimizing steric clashes and contributing to a tight binding mode.

(d) Comparison with co-crystal ligand: the co-crystal ligand showed higher binding energy, suggesting that while it may engage similar interaction types, it is less energetically favorable than HB183. The structural differences could explain this disparity, possibly relating to the flexibility or conformational stability of HB183 compared to the co-crystal ligand.

(e) Electrostatic attraction: the strong electrostatic attraction highlighted by the coulombic interactions suggests a potential for fine-tuning HB183's interaction profile through modifications to its charged groups or polar functionalities. This could lead to further enhancement of its binding affinity.

Overall, the MM-GBSA results underscore HB183's potential as a lead compound, demonstrating favorable binding characteristics that could warrant further investigation and optimization.

## 3. Conclusion

This study describes the design, synthesis, and biological evaluation of a series of Schiff base-tethered OSe derivatives. The investigated compounds exhibited variable antiproliferative effects across the tested cancer cell lines. Among them, HB183 showed comparatively consistent activity, particularly in the MCF7 breast cancer cell line, and was therefore selected for further mechanistic and computational investigations. While measurable cytotoxic effects were observed, the activity toward normal cell lines indicates that the selectivity of these compounds remains moderate and requires further optimization. Mechanistic studies suggest that HB183 influences apoptosis-related pathways in MCF7 cells. Treatment with this compound was associated with increased expression of pro-apoptotic markers, including BAX and caspases, along with reduced levels of anti-apoptotic and invasion-related proteins such as BCL-2, MMP2, and MMP9. In agreement with these findings, flow cytometry analysis revealed an increase in the sub-G1 cell population, indicating apoptosis-associated DNA fragmentation rather than arrest at a specific cell-cycle phase. These observations support the involvement of apoptotic processes; however, additional studies are required to fully clarify the underlying molecular mechanisms. Computational modeling provided supportive but not definitive insights into the interaction between HB183 and the BCL-2 protein. Docking results suggested that HB183 can adopt a favorable orientation within the BCL-2 binding pocket, and molecular dynamics simulations indicated that the ligand–protein complex remains stable over the simulation period. MM-GBSA calculations further supported the presence of energetically favorable interactions. Nevertheless, these *in silico* findings should be interpreted as complementary to the experimental data rather than as conclusive evidence of direct target engagement. In summary, the present work introduces Schiff base-linked OSe compounds as a structurally interesting class with measurable anticancer-related biological activity. HB183 represents a useful starting point for further structural refinement and biological evaluation. Future studies focusing on structure optimization, broader toxicity assessment, and *in vivo* validation will be essential to better define the therapeutic potential and limitations of this compound class.

## 4. Materials and methods

### 4.1. Chemistry

Compounds 2, 3, 6, and 7 were synthesized according to our previously reported protocols (see SI for the experimental and analytical details).^44,79,80^ See also SI for copies of IR, ^1^H-NMR, ^13^C-NMR, and Mass spectra for all the synthesized compounds.

#### 4.1.1. Procedure I: synthesis of the Schiff bases HB208, HB209, HB210

A mixture of dimethyl 4,4′-diselanediyldianiline (3) (2 mmol), allyl bromide (4.4 mmol), and NaOH was dissolved in ethanol (25 mL). Sodium borohydride (6 mmol) was then introduced gradually over the course of one hour, and the reaction was stirred for an additional two hours. The organic phase was subsequently dried and concentrated under reduced pressure, affording the intermediate 4-(allylselanyl)aniline (4) as a brown oil, which was directly used in the following step without purification.

For the next transformation, intermediate (4) was dissolved in 30 mL of ethanol containing 100 µL of acetic acid, followed by the addition of the aromatic aldehyde (4.4 mmol). The reaction mixture was heated under reflux at 85 °C for 4 hours, cooled to room temperature, and the resulting solid was collected by filtration, washed with ethanol, and recrystallized from ethanol.

#### 4.1.2. Procedure II: synthesis of the amide acids HB178 and HB179

Intermediate (4), prepared through the reduction of 4,4′-diselanediyldianiline (3) (2 mmol) followed by reaction with allyl bromide (4.4 mmol), was introduced into a stirred solution of maleic or succinic anhydride (4.4 mmol) in dry toluene (10 mL). The mixture was stirred thoroughly, and the resulting solid was collected by filtration. The solid was then washed with dichloromethane and dried under vacuum.

### 4.2. Procedure III: synthesis of the amide acids HB181 and HB183

A mixture of dimethyl 5,5-diselanediylbis(2-aminobenzoate) (7) (2 mmol), allyl bromide (4.4 mmol), and NaOH was dissolved in ethanol (25 mL). Sodium borohydride (6 mmol) was then introduced gradually over the course of one hour, and the reaction was stirred for an additional two hours. The organic phase was subsequently dried and concentrated under reduced pressure, affording the intermediate 4-(allylselanyl)aniline (8) as a yellow oil, which was directly used in the following step without purification.

For the next transformation, intermediate (8) was introduced into a stirred solution of maleic or succinic anhydride (4.4 mmol) in dry CH_2_Cl_2_ (10 mL). The mixture was stirred thoroughly, and the resulting solid was collected by filtration. The solid was then washed with dichloromethane and dried under vacuum.

#### 4.2.1. Synthesis of *N*-(4-(allylselanyl)phenyl)-1-phenylmethanimine (HB208)

Compound HB208 was prepared according to procedure I. The completion of the reaction was detected using TLC [heptane/EtOAc (4 : 1.5)]; Rf = 0.36; yellow powder; yield = 42%; MP = 43–44 °C. FT-IR (*ν*, cm^−1^): 2976 (C–H, aromatic), 1575 (C=C), 1449, 914, 811; ^1^H NMR (402 MHz, DMSO-*d*_6_) *δ* 8.58 (s, 1H, =CH), 7.87 (t, *J* = 6.5 Hz, 2H, Ar–H), 7.50–7.43 (m, 4H, Ar–H), 7.15 (t, *J* = 9.0 Hz, 2H, Ar–H), 7.10 (d, *J* = 8.2 Hz, 1H, Ar–H), 6.44 (d, *J* = 8.2 Hz, 1H, =CH), 5.96–5.73 (m, 2H, =CH_2_), 3.58 (d, *J* = 7.4 Hz, 2H, CH_2_); ^13^C NMR (101 MHz, DMSO-*d*_6_) *δ* 161.09, 150.54, 135.01, 133.59, 129.57, 129.24, 129.10, 122.25, 117.45, 116.53, 114.97, 30.15; MS (ESI) *m*/*z* (C_16_H_15_NSe): 323.9 (M^+^ + Na).

#### 4.2.2. Synthesis of 2-(((4-(allylselanyl)phenyl)imino)methyl)phenol (HB209)

Compound HB209 was prepared according to procedure I. The completion of the reaction was detected using TLC [heptane/EtOAc (4 : 1.5)]; Rf = 0.30; yellow powder; yield = 85%; MP = 59–60 °C. FT-IR (*ν*, cm^−1^): 2976 (C–H, aromatic), 1575 (C=C), 1449, 914, 811; ^1^H NMR (402 MHz, DMSO-*d*_6_) *δ* 12.95 (s, 1H, OH), 8.92 (s, 1HCH), 7.60 (d, *J* = 7.6 Hz, 1H, Ar–H), 7.50 (d, *J* = 8.2 Hz, 2H, Ar–H), 7.37 (t, *J* = 7.5 Hz, 1H, Ar–H), 7.31 (d, *J* = 8.2 Hz, 1H, Ar–H), 6.97–6.88 (m, 2H, Ar–H), 5.88 (dq, *J* = 9.6, 7.5 Hz, 1H, =CH), 4.97 (dd, *J* = 46.9, 13.4 Hz, 2H, =CH_2_), 3.62 (d, *J* = 7.4 Hz, 2H, CH_2_); ^13^C NMR (101 MHz, DMSO-*d*_6_) *δ* 163.67, 160.67, 147.16, 134.95, 133.73, 133.46, 132.94, 128.71, 122.52, 119.74, 119.58, 117.56, 117.00, 30.01; MS (ESI) *m/z* (C_16_H_15_NOSe): 314.1 (M^+^ − 2), 274.7 (M^+^ − allyl).

#### 4.2.3. Synthesis of *N*-(4-(allylselanyl)phenyl)-1-(4-chlorophenyl)methanimine (HB210)

Compound HB210 was prepared according to procedure I. The completion of the reaction was detected using TLC [heptane/EtOAc (4 : 1.5)]; Rf = 0.39; yellow powder; yield = 89%; MP = 65–66 °C. FT-IR (*ν*, cm^−1^): 2979 (C–H, aromatic), 1618 (C=C), 1487, 1402, 825; ^1^H NMR (402 MHz, DMSO-*d*_6_) *δ* 8.61 (s, 1H, =CH), 7.91 (d, *J* = 8.5 Hz, 2H, Ar–H), 7.55 (d, *J* = 8.4 Hz, 2H, Ar–H), 7.47 (d, *J* = 8.4 Hz, 2H, Ar–H), 7.18 (d, *J* = 8.3 Hz, 2H, Ar–H), 5.87 (ddt, *J* = 17.3, 9.9, 7.5 Hz, 1H, =CH), 4.95 (dd, *J* = 41.7, 13.5 Hz, 2H, =CH_2_), 3.64–3.53 (m, 2H, CH_2_); ^13^C NMR (101 MHz, DMSO-*d*_6_) *δ* 159.84, 150.19, 136.49, 135.26, 135.02, 133.52, 130.70, 129.39, 127.68, 122.31, 117.46, 30.11; MS (ESI) *m/z* (C_16_H_14_ClNSe): 337.6 (M^+^ − H), 292.8 (M^+^ − allyl).

#### 4.2.4. Synthesis of 4-((4-(allylselanyl)phenyl)amino)-4-oxobutanoic acid (HB178)

Compound HB178 was prepared according to procedure II. The completion of the reaction was detected using TLC [MeOH/EtOAc (5 : 95)]; Rf = 0.21; grey powder; yield = 86%; MP = 142–144 °C. FT-IR (*ν*, cm^−1^): 3275 (OH), 3174 (NH), 2932 (C–H, aromatic), 1690 (C=O), 1650 (C=O), 1527 (C=C), 1187, 913, 815; ^1^H NMR (402 MHz, DMSO-*d*_6_) *δ* 12.07 (s, 1H, OH), 9.94 (s, 1H, NH), 7.47 (d, *J* = 8.6 Hz, 2H, Ar–H), 7.35 (d, *J* = 8.6 Hz, 2H, Ar–H), 5.83 (ddt, *J* = 17.3, 9.9, 7.5 Hz, 1H, =CH_allyl_), 4.98–4.80 (m, 2H, =CH_2_), 3.53–3.43 (m, 2H, CH_2_), 2.55–2.43 (d, 4H, 2CH_2_); ^13^C NMR (101 MHz, DMSO-*d*_6_) *δ* 174.18, 170.54, 138.95, 135.10, 134.00, 122.70, 119.93, 117.16, 31.47, 30.52, 29.17; MS (ESI) *m/z* (C_13_H_15_NO_3_Se): 311.7 (M^+^ − H); 270.7 (M^+^ − allyl group).

#### 4.2.5. Synthesis of 4-((4-(allylselanyl)phenyl)amino)-4-oxobut-2-enoic acid (HB179)

Compound HB179 was prepared according to procedure II. The completion of the reaction was detected using TLC [MeOH/EtOAc (5 : 95)]; Rf = 0.23; white powder; yield = 96%; MP = 150–151 °C. FT-IR (*ν*, cm^−1^): 3275 (OH), 3186 (NH), 3089 (C–H, aromatic), 1696 (C=O), 1626 (C=O), 1526 (C=C), 1391, 1177, 819;^1^H NMR (402 MHz, DMSO-*d*_6_) *δ* 13.00 (s, 1H, OH), 10.36 (s, 1H, NH), 7.52 (d, *J* = 8.6 Hz, 2H, Ar–H), 7.39 (d, *J* = 8.6 Hz, 2H, Ar–H), 6.42 (d, *J* = 12.1 Hz, 1H, CH=), 6.28–6.23 (m, 1H, =CH), 5.84 (ddt, *J* = 17.3, 9.9, 7.5 Hz, 1H, =CH_allyl_), 4.90 (ddd, *J* = 13.4, 11.2, 1.3 Hz, 2H, =CH_2_), 3.61–3.44 (m, 2H, CH_2_); ^13^C NMR (101 MHz, DMSO-*d*_6_) *δ* 167.21, 163.65, 138.11, 135.05, 133.76, 132.12, 130.71, 124.08, 120.51, 117.29, 30.38; MS (ESI) *m/z* (C_13_H_13_NO_3_Se): 309.7 (M^+^ − H); 268.6 (M^+^ − allyl group).

#### 4.2.6. Synthesis of 4-((4-(allylselanyl)-3-(methoxycarbonyl)phenyl)amino)-4-oxobutanoic acid (HB181)

Compound HB181 was prepared according to procedure III. The completion of the reaction was detected using TLC [MeOH/EtOAc (5 : 95)]; Rf = 0.30; grey powder; yield = 93%; MP = 104–105 °C. FT-IR (*ν*, cm^−1^): 3259 (OH), 3156 (NH), 2952 (C–H, aromatic), 1685 (C=O), 1579 (C=O), 1512 (C=C), 1250, 909, 789;^1^H NMR (402 MHz, DMSO-*d*_6_) *δ* 12.13 (s, 1H, OH), 10.51 (d, *J* = 8.8 Hz, 1H, NH), 8.12 (d, *J* = 8.8 Hz, 1H, Ar–H), 7.91 (t, *J* = 2.7 Hz, 1H, Ar–H), 7.65 (dd, *J* = 8.6, 1.6 Hz, 1H, Ar–H), 5.88–5.78 (m, 1H, =CH_allyl_), 4.94–4.84 (ddd, *J* = 13.4, 11.2, 1.3 Hz, 2H, =CH_2_), 3.81 (s, 3H, CH_3_), 3.56 (d, *J* = 7.5 Hz, 2H, CH_2_), 2.57 (d, *J* = 6.5 Hz, 2H, CH_2_), 2.49 (t, *J* = 5.3 Hz, 2H, CH_2_); ^13^C NMR (101 MHz, DMSO-*d*_6_) *δ* 173.95, 170.72, 167.41, 139.03, 138.78, 135.08, 1349.89, 134.82, 123.22, 122.08, 118.73, 117.56, 32.21, 30.61, 29.13, 14.37; MS (ESI) *m/z* (C_15_H_17_NO_5_Se): 393.6 (M^+^ + Na).

#### 4.2.7. Synthesis of 4-((4-(allylselanyl)-3-(methoxycarbonyl)phenyl)amino)-4-oxobut-2-enoic acid (HB183)

Compound HB183 was prepared according to procedure III. The completion of the reaction was detected using TLC [MeOH/EtOAc (5 : 95)]; Rf = 0.28; white powder; yield = 92%; MP = 97–98 °C. FT-IR (*ν*, cm^−1^): 3360 (OH), 3165 (NH), 3006 (C–H, aromatic), 1710 (C=O), 1683 (C=O), 1591 (C=C), 1235, 833, 787; ^1^H NMR (402 MHz, DMSO-*d*_6_) *δ* 12.93 (s, 1H, OH), 10.71 (s, 1H, NH), 8.12 (d, *J* = 8.6 Hz, 1H, Ar–H), 7.92 (d, *J* = 2.1 Hz, 1H, 1H, Ar–H), 7.69 (dd, *J* = 8.6, 2.1 Hz, 1H, 1H, Ar–H), 6.52 (d, *J* = 12.0 Hz, 1H, =CH), 6.32–6.26 (d, *J* = 12.0 Hz, 1H, CH=), 5.84 (ddt, *J* = 17.4, 9.9, 7.5 Hz, 1H, =CH_allyl_), 5.02–4.83 (ddd, *J* = 13.4, 11.2, 1.3 Hz, 2H, CH_2_), 3.80 (s, 3H, CH_3_), 3.63–3.53 (m, 2H, CH_2_); ^13^C NMR (101 MHz, DMSO-*d*_6_) *δ* 167.22, 167.01, 163.89, 138.50, 138.34, 138.17, 134.83, 134.80, 132.73, 130.44, 124.37, 119.42, 117.67, 30.50, 14.33; MS (ESI) *m/z* (C_15_H_15_NO_5_Se): 391.6 (M^+^ + Na).

### 4.3. Biological assays

#### 4.3.1. The percentage of growth inhibition assay

All cancer cell lines used in this study were obtained from Vacsera (Giza, Egypt). The cytotoxic potential of the OSe compounds (HB178, 179, 181, 183, 208, 209, and 210) was evaluated using the sulforhodamine B (SRB) colorimetric assay.^83,95^ The aforementioned analogues were examined against a panel of ten human cancer cell lines, including HN9, HuH7, HEPG2, FaDu, MCF7, A549, HCT_116_, and A375, all sourced from the American Type Culture Collection (ATCC). To assess the safety and selectivity of the synthesized derivatives, cytotoxicity was also examined in normal human cell lines. These included HSF (human skin fibroblasts), which are commonly used in studies ranging from skin aging to wound healing and oncology, and the OEC (oral epithelial cells), known for their regenerative potential and relevance in regenerative medicine and tissue engineering. The normal human cell lines used in this study, HSF and OEC, were obtained from the Pharmacology Unit, Cancer Biology Department, National Cancer Institute (NCI), Cairo University. These cell lines have been available and maintained in the institute for an extended period and were originally purchased by the institute from the American Type Culture Collection (ATCC, USA). All experimental work and biological evaluations reported in the manuscript were carried out at the National Cancer Institute, Cairo University.

All experiments were performed in triplicate, and results are expressed as mean ± SD. For comparisons between two groups (*e.g.*, a single compound in cancer cells *vs.* normal cells), an unpaired Student's *t*-test was used. For comparisons involving three or more groups (*e.g.*, multiple compounds or multiple cell lines), one-way ANOVA followed by Tukey's post-hoc test was applied. Differences were considered statistically significant at *p* < 0.05.^96–98^

#### 4.3.2. Cytotoxic inhibitory concentration 50 (IC_50_) assay

To gain deeper insight into the cytotoxic potential of the most potent synthesized Schiff base-tethered OSe (HB178, HB179, HB183, HB209, and HB210), their half-maximal inhibitory concentrations (IC_50_) were determined. This evaluation was performed on the cancer cell lines that showed the highest growth inhibition rates in the initial screening phase (details available in the Supplementary Information). Accordingly, the IC_50_ values were measured against HEPG2, A549, HCT_116_, HuH7, FaDu, and MCF7 cell lines using the SRB assay, as previously described.^83,99,100^

#### 4.3.3. Apoptosis-related gene protein expression

To examine the apoptotic potential of the most active Schiff base-tethered OSe compounds, HB183 protein expression analysis was performed on the MCF7. This study aimed to evaluate the levels of key apoptosis-related proteins and uncover the underlying molecular mechanisms responsible for the compounds' cytotoxic effects using enzyme-linked immunosorbent assay.^56,101^ The analysis compared treated and untreated cells to identify any significant changes in protein expression, providing insights into the pro-apoptotic pathways triggered by these Schiff base-tethered OSe compounds.

#### 4.3.4. Flow cytometry analysis of DNA content (compound HB183)

To assess the effects of analogue HB183 on cell cycle progression, MCF-7 breast cancer cells^94,102^ (2 × 10^5^ cells per well) were seeded in 12-well plates and treated with varying concentrations of the test compounds over different incubation periods. Following treatment, cells were harvested and fixed overnight in ice-cold 70% ethanol diluted in phosphate-buffered saline (PBS).

After fixation, the cells were washed and resuspended in PBS containing 40 µg mL^−1^ propidium iodide, 0.1 mg mL^−1^ RNase A (Sigma, USA), and 0.1% Triton X-100. The cell suspension was incubated at 37 °C for 30 minutes in the dark to ensure adequate staining and RNA degradation. Subsequently, the samples were analyzed using a flow cytometer (Becton Dickinson, San Jose, CA, USA) equipped with a 488 nm argon laser. The resulting data were used to determine the distribution of cells across different phases of the cell cycle.

### 4.4. *In silico* studies

#### 4.4.1. Molecular docking

The AutoDock Vina^103^ and PyMOL^104^ were used for docking the lead analogue (HB183) towards the BCL-2 target receptor after its preparation by energy minimization and optimization of partial charges.^105,106^ The grid size was set to 30 × 30 × 30 *xyz* points, and the grid center was set to (*x*, *y*, *z*): 9.92, 20.528, and 19.532, with a grid spacing of 1000 Å. The PDB was searched, and the 4IEH ID was selected and prepared using correction, hydrogenation (3D), and energy minimization.^107^ Then, analogue (HB183) was compared to the BCL-2 co-crystal inhibitor. Additionally, a redocking procedure for the BCL-2 co-crystal inhibitor approved the validity of the applied software (RMSD < 2 Å), besides the closely similar binding mode (Supporting Information).^108^

#### 4.4.2. Molecular dynamics simulation

The Desmond package of Schrödinger LLC was applied to carry out the molecular dynamics simulation at 500 ns for analogue HB183, along with the co-crystal ligand (SI).

#### 4.4.3. MM-GBSA calculations

The thermal_mmgbsa.py Python script of Schrödinger LLC was used to calculate the Molecular Mechanics Generalized Born Surface Area (MM-GBSA) energies (SI).

### 4.5. Statistical analysis

Data were represented as mean ± standard deviation (SD). The data were analyzed by using a one-way analysis of variance (ANOVA) test. To assess the significance of differences, the Tukey post-hoc test was used. P-values less than 0.05 were considered to be statistically significant. Graphs were performed using the Prism software program (GraphPad Prism software, version 8.02, CA, USA), and analysis of data was performed using GraphPad InStat, Version 8.02.

## Author contributions

Conceptualization: Saad Shaaban and Ahmed A. Al-Karmalawy; supervision: Saad Shaaban and Ahmed A. Al-Karmalawy; data curation, visualization, methodology, and writing – review & editing: Saad Shaaban, Samia S. Hawas, Asma M. Elsharif, Marwa Sharaky, Hussein Ba-Ghazal, Mohamed Alaasar, Fatema S. Alatawi, Khadra B. Alomari, Mohamed E. Eissa, Arwa Omar Al Khatib, Radwan Alnajjar, Ahmed A. Al-Karmalawy. Finally, all authors revised and approved the final submitted version of the manuscript.

## Conflicts of interest

The authors declare no competing financial interest.

## Funding

This work was supported by the Deanship of Scientific Research, Vice Presidency for Graduate Studies and Scientific Research, King Faisal University, Saudi Arabia [Grant No. KFU260105].

## Supplementary Material

RA-016-D5RA09238H-s001

## Data Availability

The data supporting this article have been included in the manuscript and as part of the supplementary Information (SI). Supplementary information: materials and methods; copies of the ^1^H-NMR & ^13^C-NMR, IR, and MS spectra of the synthesized compounds; biological evaluation: IC_50_ calculation curves of all synthesized compounds; cell cycle analysis histograms; *in silico* figures; methodologies for molecular dynamics simulation and MM-GBSA calculations. See DOI: https://doi.org/10.1039/d5ra09238h.
