# Supplementary material for: Organoselenium-based Schiff bases and amidic acid derivatives as promising anticancer agents targeting breast cancer by downregulating BCL-2: design, synthesis, and biological evaluation
Source: RSC Adv. 2026 Feb 2;16(7):6539–61. doi: 10.1039/d5ra09238h (PMC12862577; doi:10.1039/d5ra09238h)
Supplement: RA-016-D5RA09238H-s001 [file RA-016-D5RA09238H-s001.pdf]

Supplementary Information (SI) for RSC Advances.
This journal is © The Royal Society of Chemistry 2026

Supporting Information

Organoselenium-Based Schiff Bases and Amidic Acid Derivatives as Promising Anticancer Agents Targeting Breast Cancer by Downregulating BCL-2: Design, Synthesis, and Biological Evaluation

Saad Shaaban[1,*], Samia S. Hawas[2], Asma M. Elsharif[3], Marwa Sharaky[4], Hussein Ba-Ghazal[1], Mohamed Alaasar[5], Fatema S. Alatawi[6], Khadra B. Alomari[7], Mohamed E. Eissa[8], Arwa Omar Al Khatib[9], Radwan Alnajjar[10,11], Ahmed A. Al-Karmalawy[12,2,*]

[1] Department of Chemistry, College of Science, King Faisal University, Al-Ahsa 31982, Saudi Arabia.
[2] Department of Pharmaceutical Chemistry, Faculty of Pharmacy, Horus University-Egypt, New Damietta 34518, Egypt.
[3] Department of Chemistry, College of Science, Imam Abdulrahman Bin Faisal University, Dammam 31441, Saudi Arabia.
[4] Cancer Biology Department, Pharmacology Unit, National Cancer Institute (NCI), Cairo University, Cairo, Egypt.
[5] Department of Chemistry, Faculty of Science, Cairo University, Giza, Egypt.
[6] Department of Biochemistry, Faculty of Science, University of Tabuk, Tabuk, Saudi Arabia.
[7] Jazan University, Department of Physical Sciences, Chemistry Division, P.O. Box 114, 45142, Jazan, Kingdom of Saudi Arabia.
[8] Department of Chemistry, College of Science, Imam Mohammad Ibn Saud Islamic University (IMSIU), Riyadh 11623, Saudi Arabia.
[9] Faculty of Pharmacy, Al-Ahliyya Amman University, Amman, Jordan.
[10] Department of Chemistry, Faculty of Science, University of Benghazi, Benghazi, Libya.
[11] CADD Unit, PharmD, Faculty of Pharmacy, Libyan International University, Benghazi, Libya.
[12] Department of Pharmaceutical Chemistry, College of Pharmacy, The University of Mashreq, Baghdad 10023, Iraq.

*Corresponding authors:

Ahmed A. Al-Karmalawy; Email: akarmalawy@horus.edu.eg

Saad Shaaban; Email: sibrahim@kfu.edu.sa

Methods and Materials

Melting points (MP) in °C were recorded on the Gallenkamp instrument. The IR spectra (KBr, ύ cm^{-1}) were recorded at King Faisal University on a Mattson 5000 FTIR Spectrophotometer. The ^{1}H NMR (400 MHz) and the ^{13}C NMR (100.6 MHz) spectra were recorded at Martin-Luther-University, Halle, Germany, using a Varian VXR spectrometer employing the TMS internal reference and DMSO-d$_6$ and pyridine-d$_6$ solvents. The Mass spectra were measured at Martin Luther University, Halle, Germany, on an Agilent 6530 LC Q-TOF instrument.

Chemistry

4-Aminophenylselenocyanate (**2**) [1], bis(4-aminophenyl)diselenide (**3**) [2], methyl 2-amino-5-selenocyanatobenzoate (**6**) [3, 4], and dimethyl 5,5-diselanediylbis(2-aminobenzoate) (**7**) [3, 4] were synthesized as per the reported protocols.

4-Selenocyanatoaniline (2) [1]

Selenium dioxide (6 mmol) was added under stirring to a solution of malononitrile (3 mmol) in DMSO (15 mL). The mixture was stirred at room temperature for 15 min in order to obtain triselenium dicyanide. When the exothermic reaction had finished, aniline (5 mmol) was added. The mixture was stirred for 20 min. Water (150 mL) was added to the reaction mixture, and the resulting precipitate (4-selenocyanatobenzenamine) was filtered off, dried, and used without further purifications.

4-Aminophenylselenocyanate (**2**) is a yellow solid, melting point = 73–74 °C. ^{1}H NMR (400 MHz, $CDCl_3$) δ 7.44 (d, J = 8.4 Hz, 2H, Ar-H), 6.64 (d, J = 8.4 Hz, 2H, Ar-H), 3.95 (s, 2H, NH_2).

Synthesis of 4,4'-diselanediyldianiline (3) [1]

Under argon, $NaBH_4$ (3 mmol) was added in small portions with caution to a solution of 4-selenocyanatobenzenamine (1 mmol) in absolute ethanol (40 mL). The mixture was stirred at room temperature for 2 h. The solvent was removed under reduced pressure, and the remaining residue was dissolved in dichloromethane, washed with water (350 mL). The organic layer was separated, dried with anhydrous Na_2SO_4, and removed under vacuum. The residue was purified by chromatography on silica gel (petroleum ether: ethyl acetate 4:2).

4,4′-Diselanediyldianiline (**2**) was obtained as pale-yellow crystals (82 % yield. Mp: 78–80 °C. ^{1}H NMR (400 MHz, $CDCl_3$) δ 7.3 (m, 4H, Ar-H), 6.5 (m, 4H, Ar-H), 3.7 ppm (s, 4H, NH_2).

Synthesis of methyl 2-amino-5-selenocyanatobenzoate (6) [3, 4]

Methyl 2-amino-5-selenocyanatobenzoate (**6**) was synthesized from the reaction of methyl 2-aminobenzoate with TSD prepared *in situ* from $CH_2(CN)_2$ and SeO_2 in 96% yield. Briefly, SeO_2 (30 mmol,

3300 mg) was added to $CH_2(CN)_2$ (15 mmol, 1000 mg) in 10 mL DMSO-D6, and the mixture was stirred for 20 min at RT. Next, the mixture was filtered off to get rid of any formed black selenium, and methyl 2-aminobenzoate (12.5 mmol, 1800 mg) was then added, and the reaction mixture was stirred for a further 2 hrs. Finally, adding 10 gm of ice terminated the reaction, and the formed precipitate was filtered, washed several times with H_2O and Na_2CO_3 solution, dried, and recrystallized from petroleum ether.

2-Amino-5-selenocyanatobnzoate was isolated as reddish solid; yield:3072 mg (96%); MP=118-119 ºC; Rf: 0.4 (petroleum ether/ ethyl acetate 4:2). IR(KBr): λ_{max}.cm^{-1}:3475, 3366, 2946, 2147, 1691. ^{1}H NMR (400 MHz, DMSO-D6-d_6) δ 8.02 (s, 1H, Ar-H), 7.57 (d, *J* = 8.8 Hz, 1H, Ar-H), 7.08 (d, 1H, Ar-H), 6.84 (s, 2H, NH_2), 3.82 (s, 3H, OCH_3).^{13}C NMR (101 MHz, DMSO-D6-d_6) δ 166, 152, 140, 137, 118, 109, 105, 105, 51. MS (EI, 70 ev) *m/z* (%) = 259.35 (M+3H, 2.39),117 (29.02), 87 (26.6), 75 (2.70), 59 (100.0, base peak).

Synthesis of dimethyl 5,5-diselanediylbis(2-aminobenzoate) (7) [3, 4]

Dimethyl 5,5-diselanediylbis(2-aminobenzoate) (**7**) was synthesized from the reaction of **6** and sodium hydroxide in 92% yield. Briefly, compound **6** (4 mmol, 1000 mg) was dissolved in EtOH (20 mL), and then sodium hydroxide (4 mmol,160 mg) was added. The reaction mixture was stirred for 2 hours at room temperature, and the resulting precipitate was filtered, washed several times with H_2O, and recrystallized from chloroform.

Dimethyl 5,5-diselanediylbis(2-aminobenzoate) was isolated as a yellow solid; yield: 1692.43 mg (92%); MP=138- 139 °C; Rf =0.5(petroleum ether/ ethyl acetate 4:3). IR(KBr): λ_{max}.cm^{-1}: 3455, 3344, 2931, 1684. ^{1}HNMR (400 MHz, DMSO-D6) δ7.70 (s,2H, Ar-H), 7.44(d, *J* = 8.6 Hz, 2H, Ar-H), 7.00(s, 4H,$2NH_2$), 6.77(d, *J* = 8.7 Hz, 2H, Ar-H), 3.74(s, 6H,$2OCH_3$). ^{13}CNMR (101 MHz, DMSO-D6-d_6) δ167, 151, 140, 138, 117, 113, 108, 51. MS (EI, 70 ev) *m/z* (%) = 460.15(M+H, 20.76), 459.15(M, 5.20) or 230(24.42), 119(9.45), 91(100.0, base peak),65(8.88).

N-(4-(Allylselanyl)phenyl)-1-phenylmethanimine (HB208)

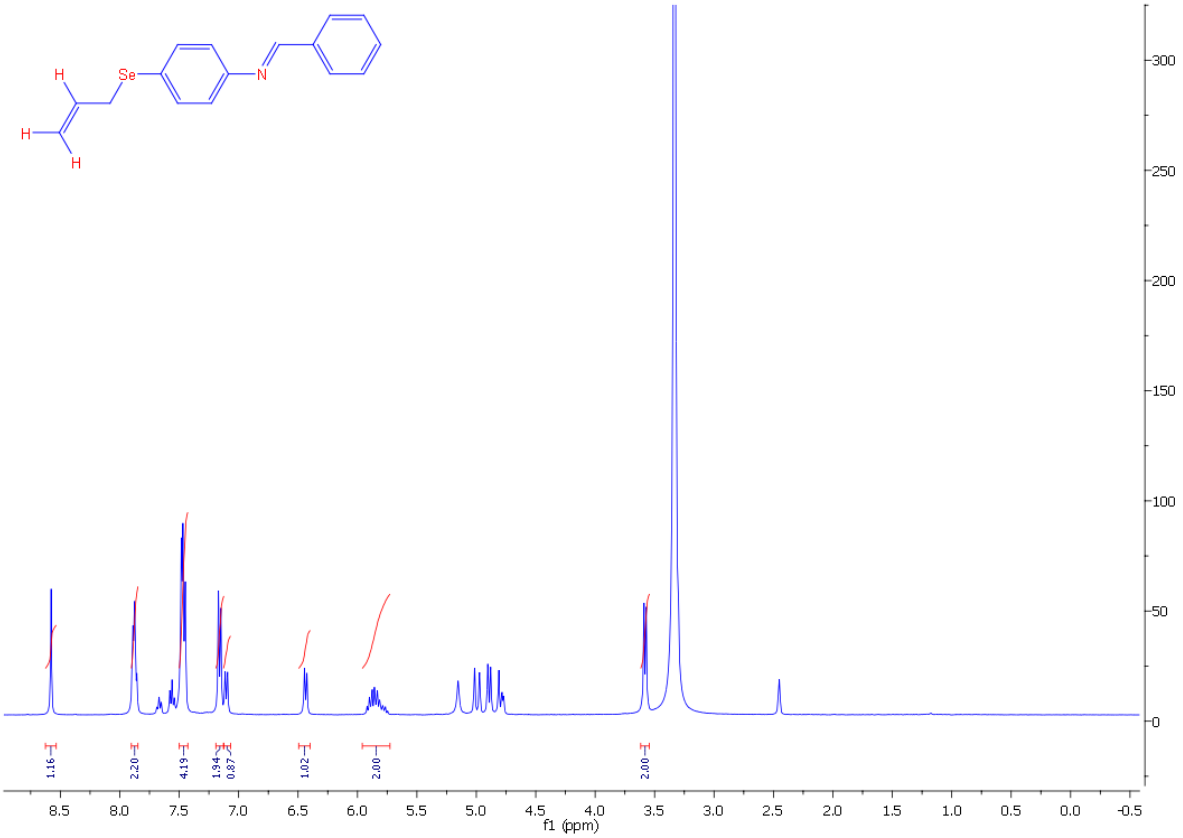

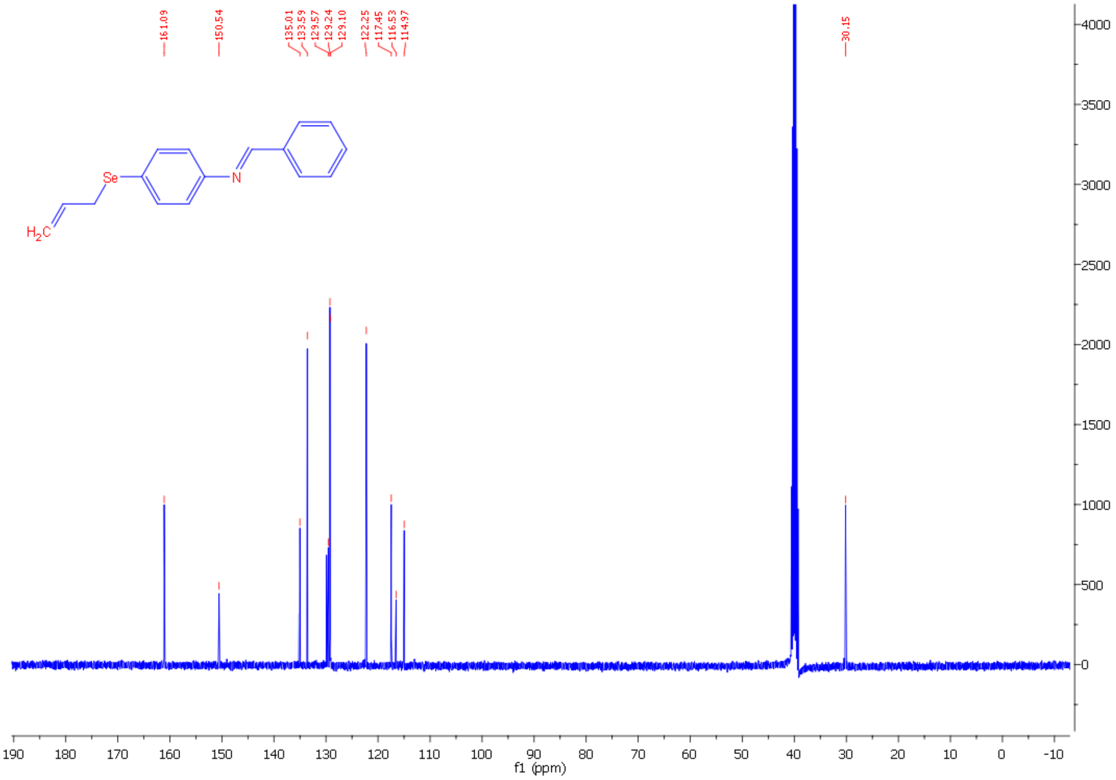

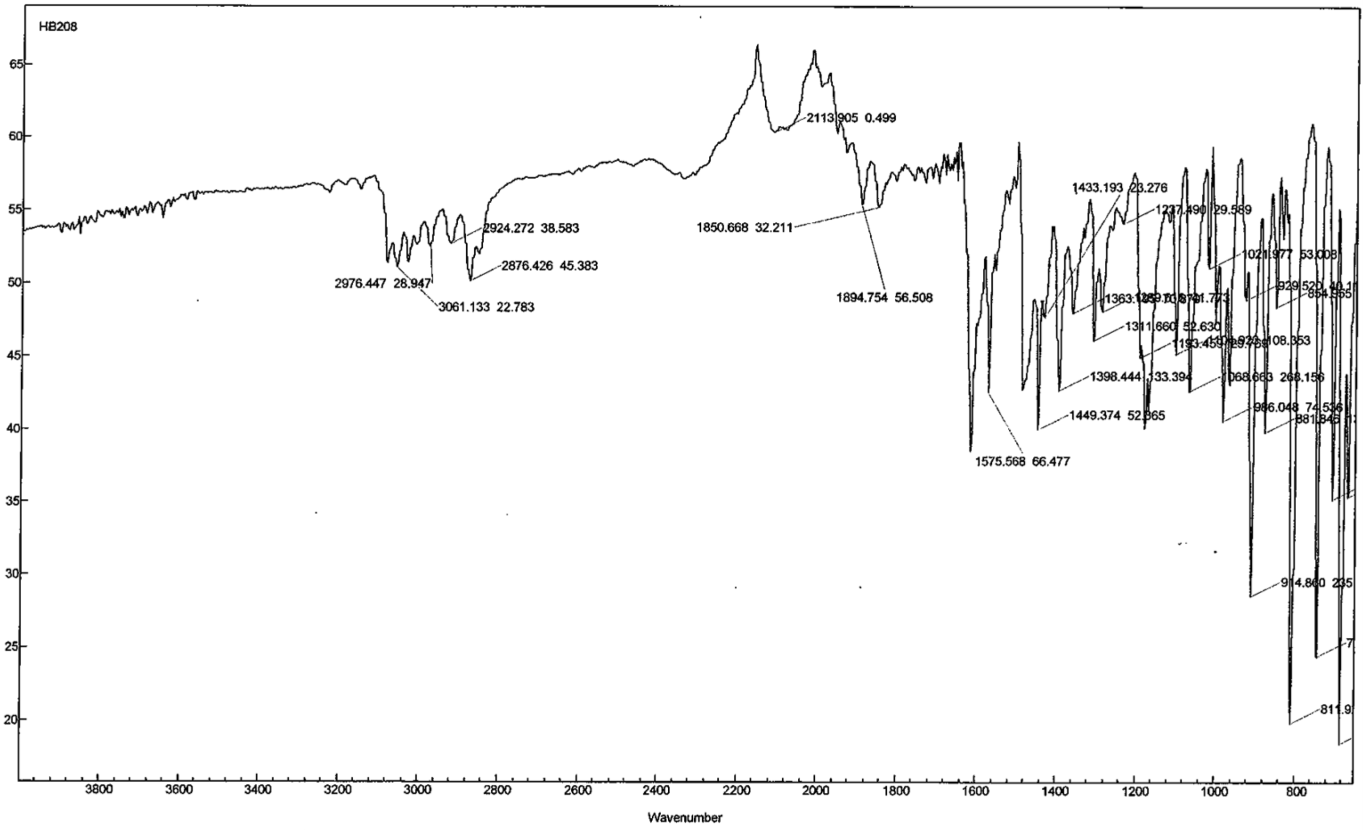
HB208
2113.905 0.499
2924.272 38.583
2876.426 45.383
2976.447 28.947
3061.133 22.783
1850.668 32.211
1894.754 56.508
1433.193 23.276
1237.490 29.589
1021.977 53.008
1311.660 52.630
1398.444 133.394
1068.663 268.156
1449.374 52.365
986.048 74.536
1575.568 66.477
914.860 235
811.9
Wavenumber

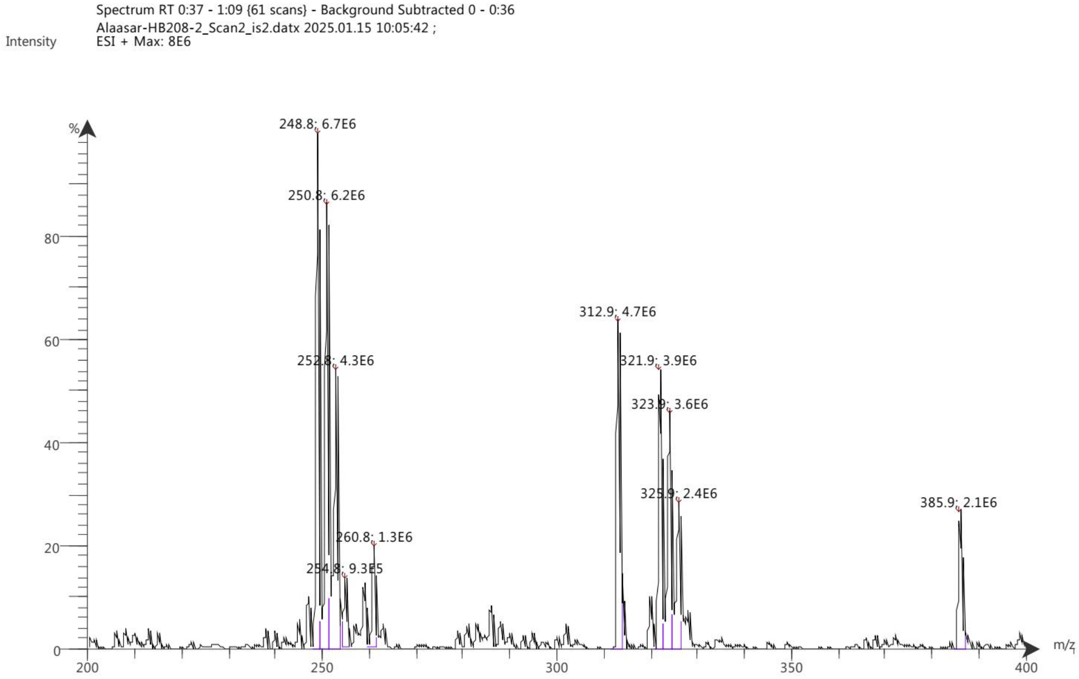
Spectrum RT 0:37 - 1:09 (61 scans) - Background Subtracted 0 - 0:36
Alaasar-HB208-2_Scan2_is2.datx 2025.01.15 10:05:42 ;
Intensity
ESI + Max: 8E6
248.8; 6.7E6
250.8; 6.2E6
252.8; 4.3E6
260.8; 1.3E6
254.8; 9.3E5
312.9; 4.7E6
321.9; 3.9E6
323.9; 3.6E6
325.9; 2.4E6
385.9; 2.1E6
m/z

2-(((4-(Allylselanyl)phenyl)imino)methyl)phenol (HB209)

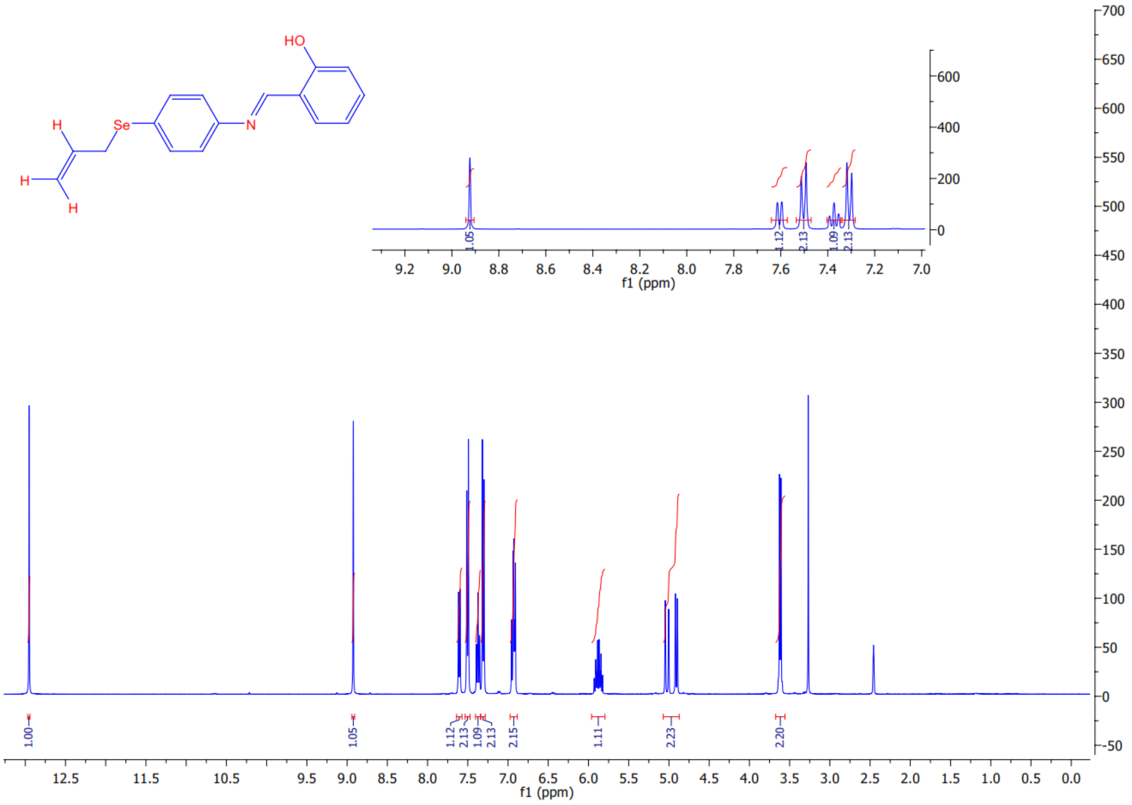

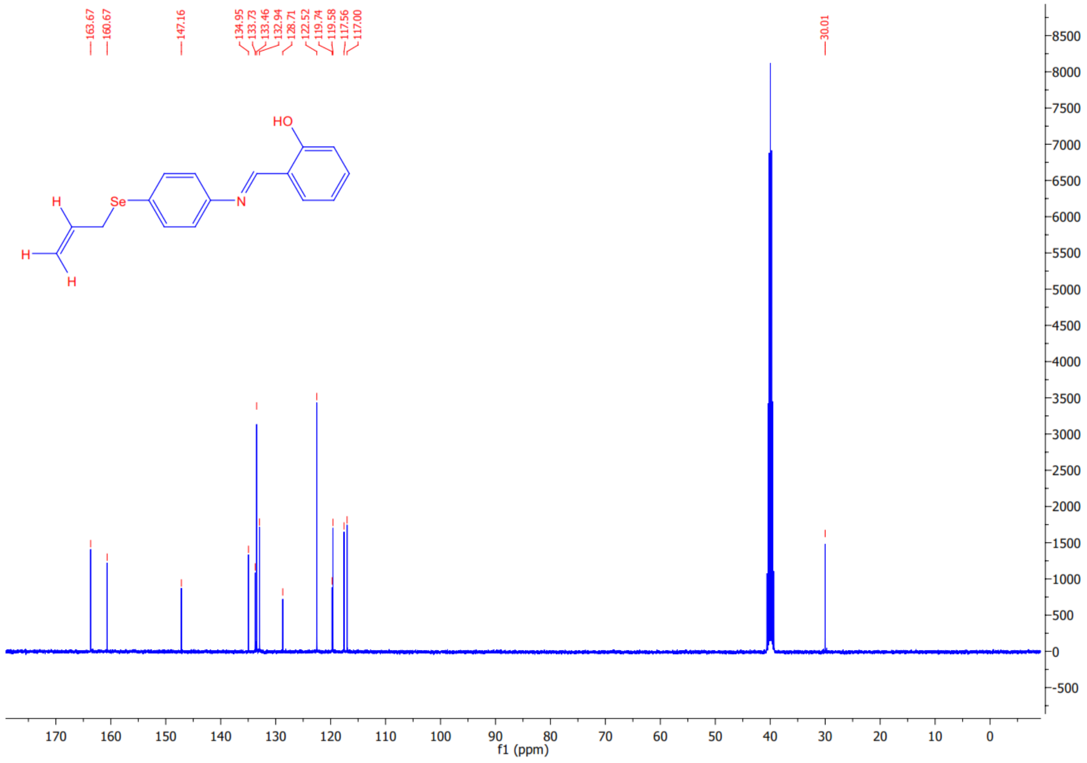

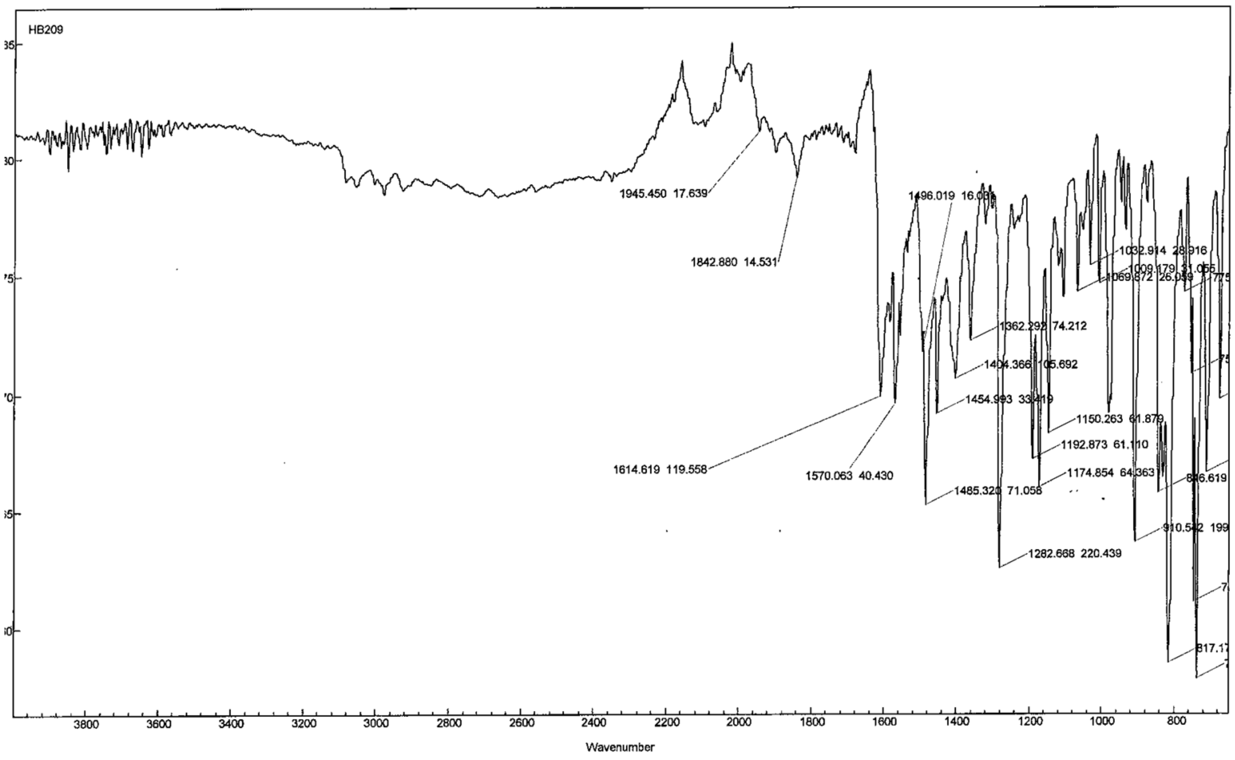
HB209
1945.450 17.639
1842.880 14.531
1496.019 16.086
1032.914 28.916
1009.179 31.055
1069.872 26.059
1362.292 74.212
1404.366 105.692
1454.993 33.419
1150.263 61.879
1192.873 61.110
1614.619 119.558
1570.063 40.430
1174.854 64.363
1485.320 71.058
1282.668 220.439
3800
3600
3400
3200
3000
2800
2600
2400
2200
2000
1800
1600
1400
1200
1000
800
Wavenumber

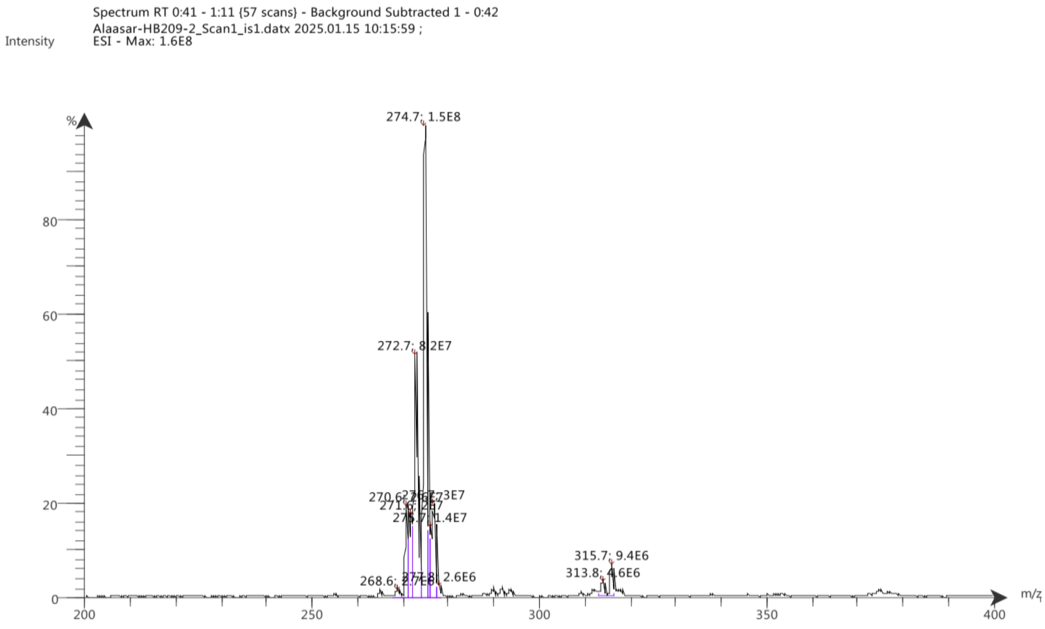
Spectrum RT 0:41 - 1:11 (57 scans) - Background Subtracted 1 - 0:42
Alaasar-HB209-2_Scan1_is1.datx 2025.01.15 10:15:59 ;
ESI - Max: 1.6E8
Intensity
%
274.7; 1.5E8
272.7; 8.2E7
275.7; 1.4E7
315.7; 9.4E6
313.8; 4.6E6
268.6; 2.7E6
277.8; 2.6E6
80
60
40
20
0
200
250
300
350
400
m/z

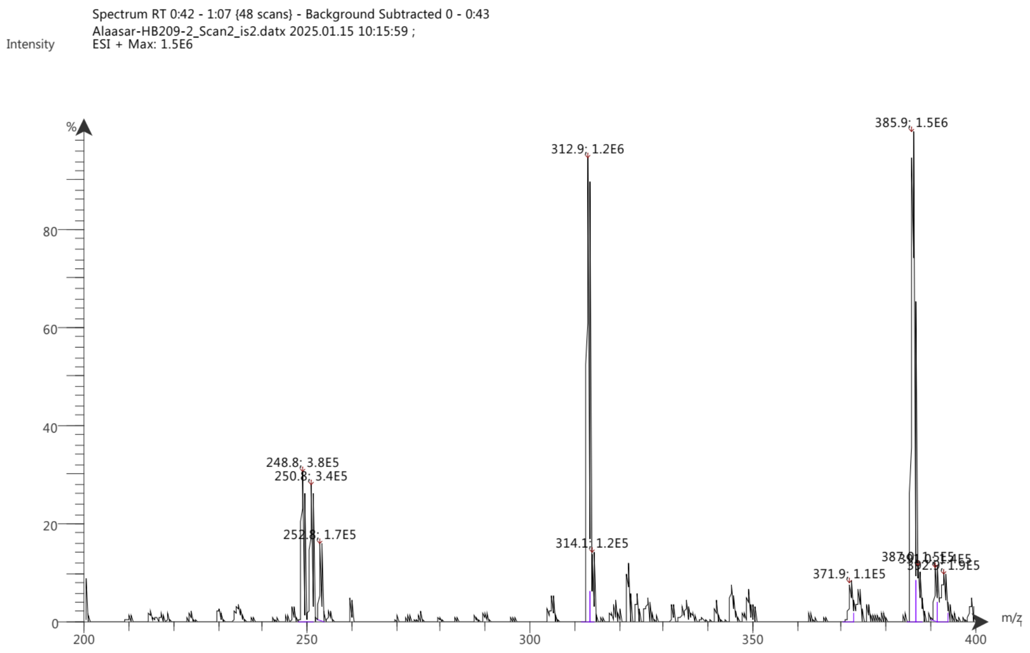
Spectrum RT 0:42 - 1:07 (48 scans) - Background Subtracted 0 - 0:43
Alaasar-HB209-2_Scan2_is2.datx 2025.01.15 10:15:59 ;
Intensity
ESI + Max: 1.5E6
%
385.9; 1.5E6
312.9; 1.2E6
248.8; 3.8E5
250.8; 3.4E5
252.8; 1.7E5
314.1; 1.2E5
371.9; 1.1E5
m/z
0
20
40
60
80
200
250
300
350
400

N-(4-(Allylselanyl)phenyl)-1-(4-chlorophenyl)methanimine (HB210)

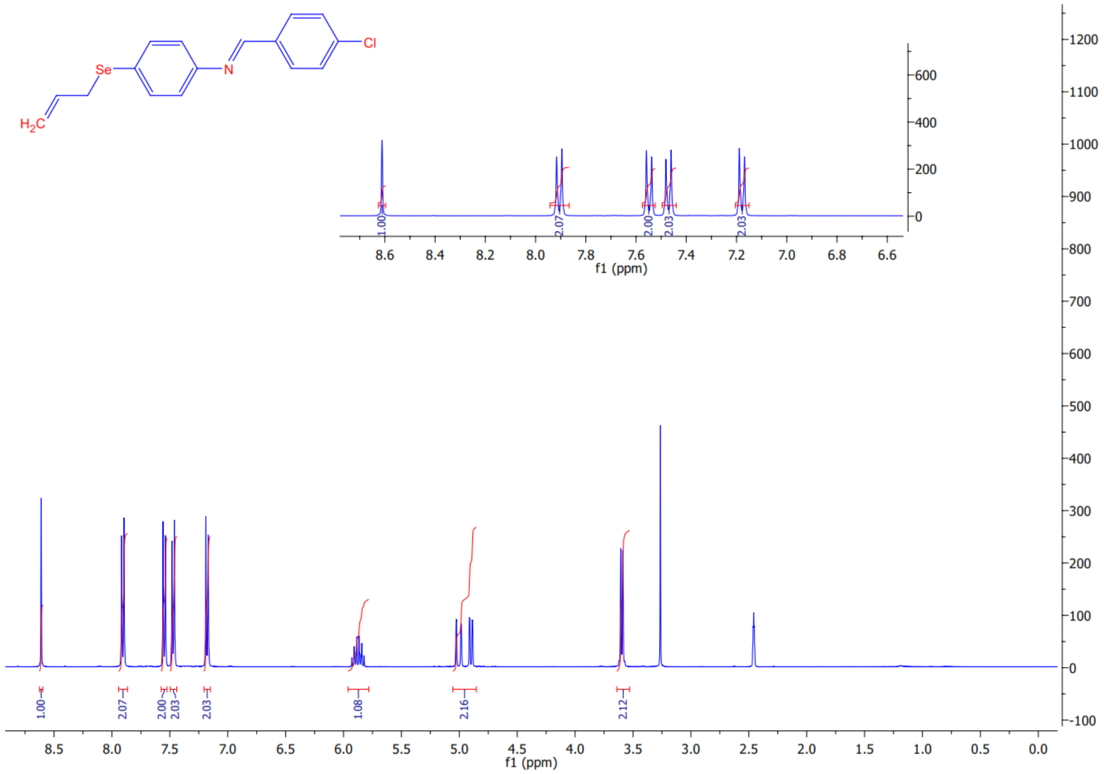

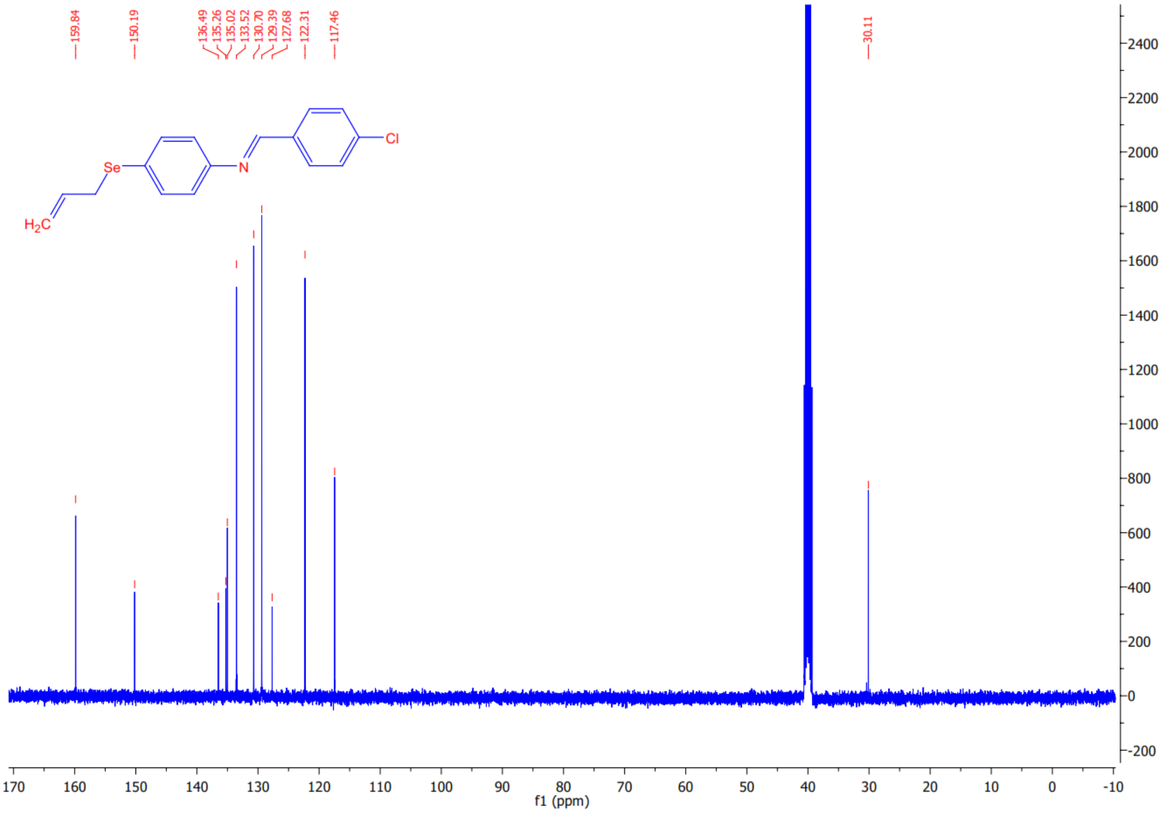

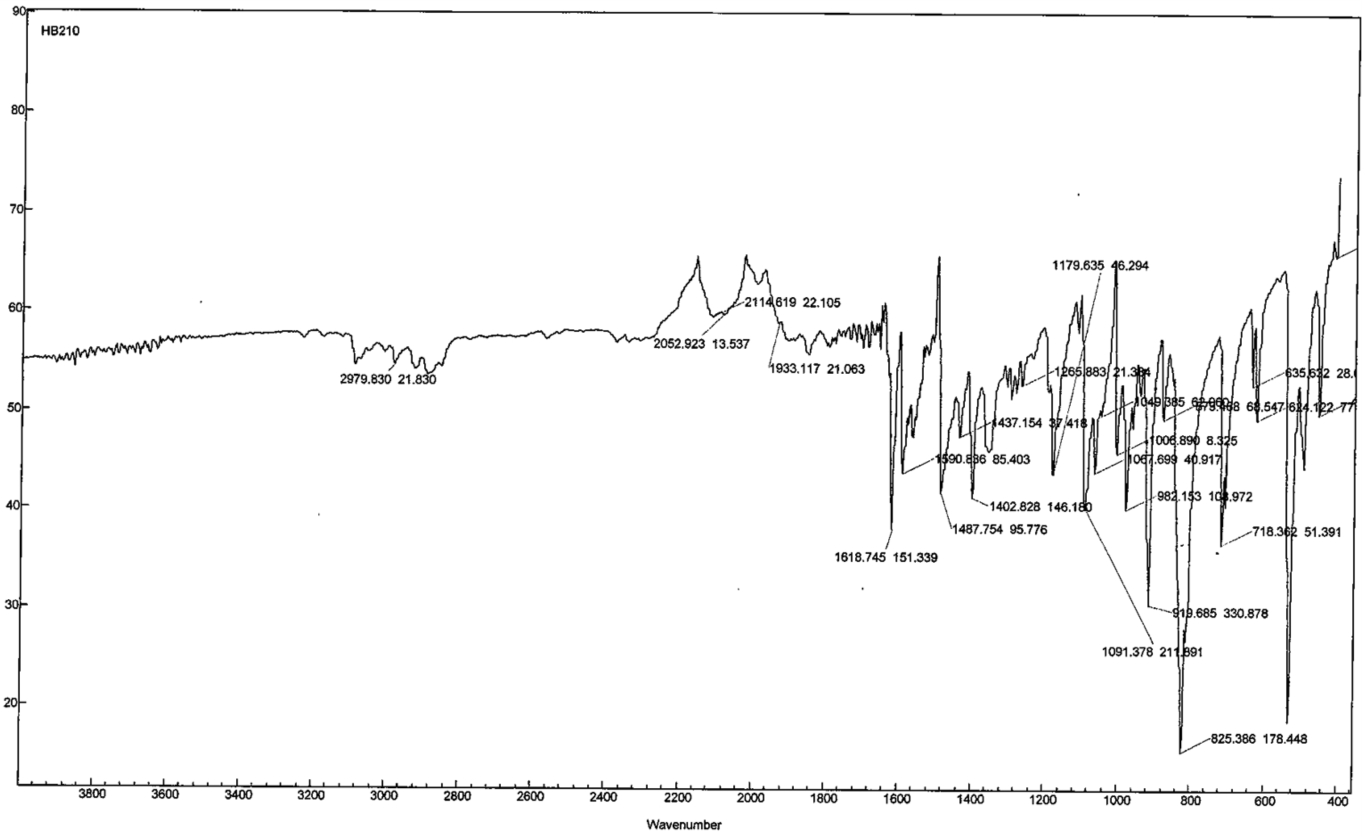
HB210
2979.830 21.830
2052.923 13.537
2114.619 22.105
1933.117 21.063
1618.745 151.339
1590.836 85.403
1487.754 95.776
1437.154 37.418
1402.828 146.180
1179.635 46.294
1265.883 21.384
1091.378 211.891
1067.699 40.917
1006.890 8.325
982.153 103.972
919.685 330.878
825.386 178.448
718.362 51.391
635.632
Wavenumber
3800
3600
3400
3200
3000
2800
2600
2400
2200
2000
1800
1600
1400
1200
1000
800
600
400
90
80
70
60
50
40
30
20

4-((4-(Allylselanyl)phenyl)amino)-4-oxobutanoic acid (HB178)

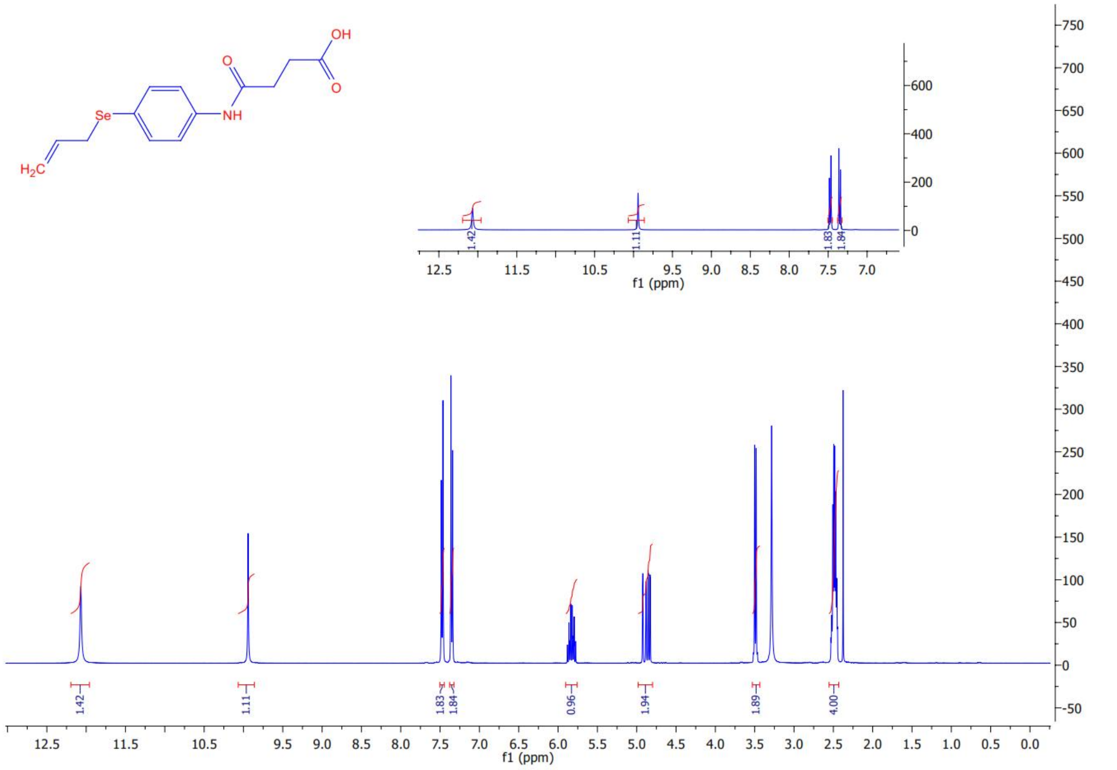

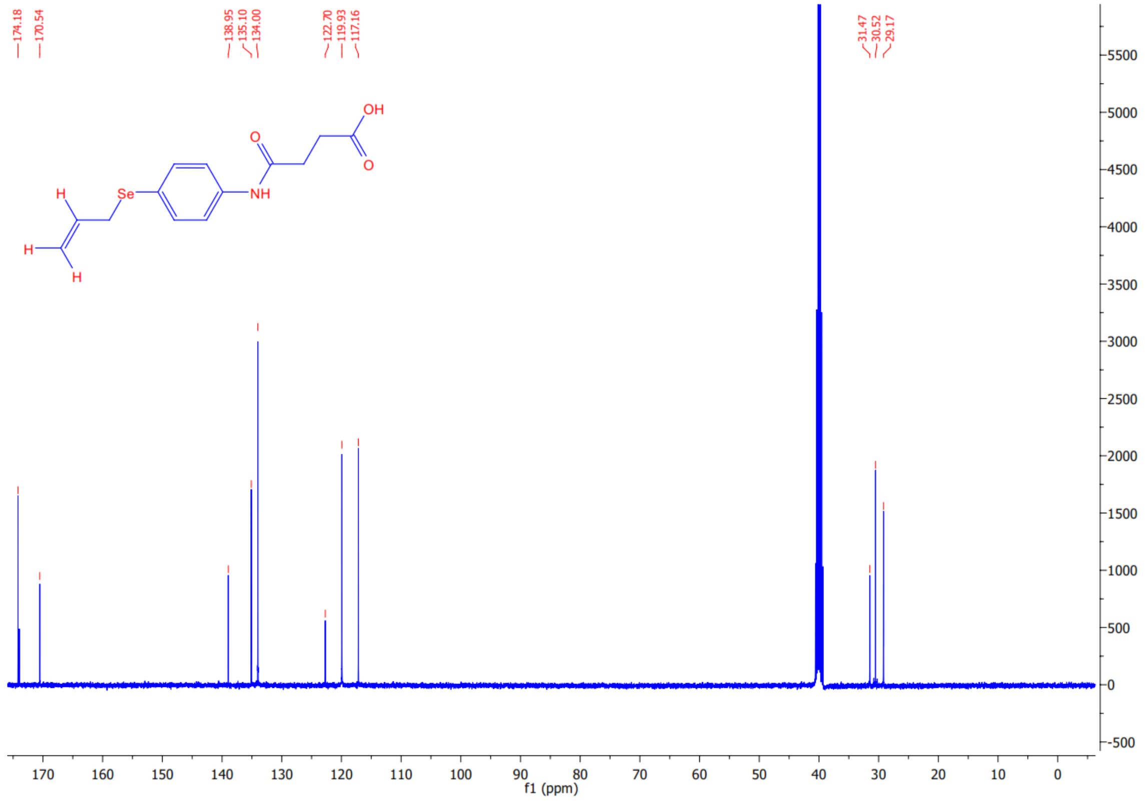

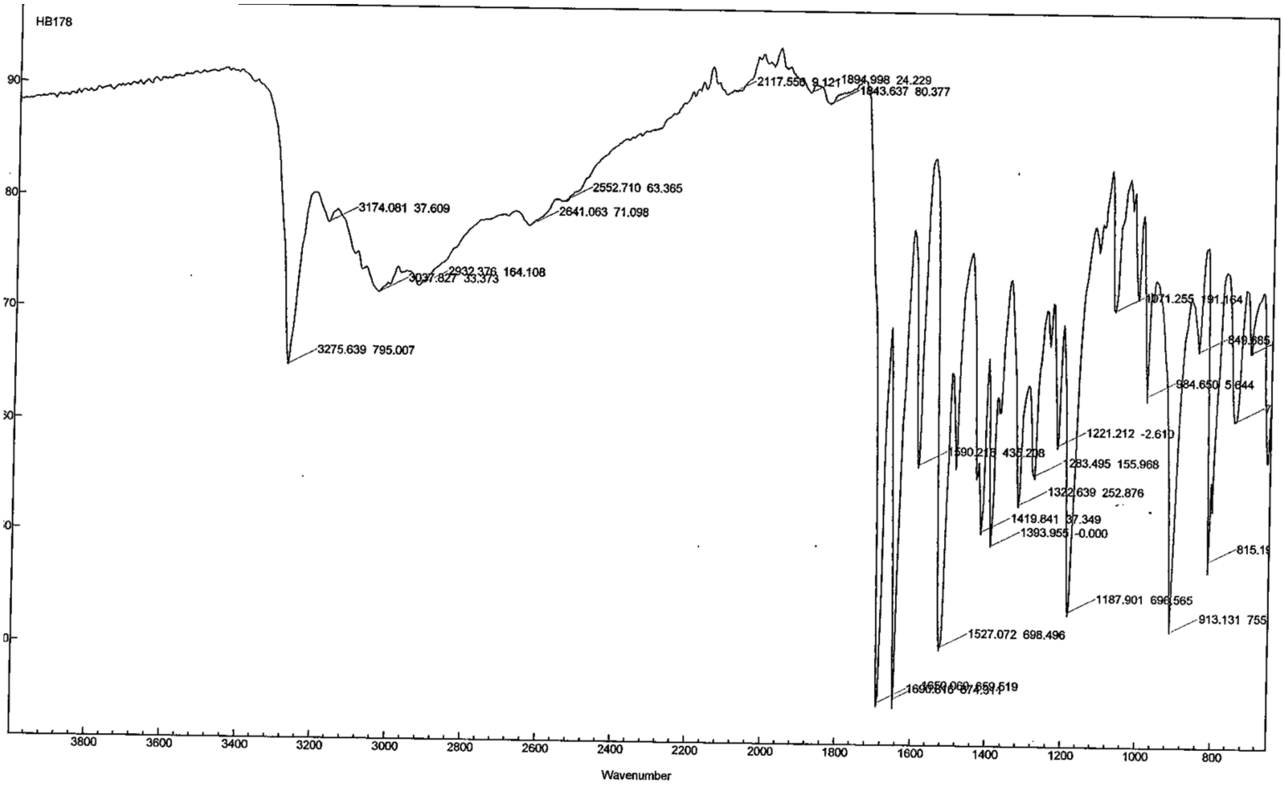
HB178
3275.639 795.007
3174.081 37.609
3037.827 33.373
2932.376 164.108
2641.063 71.098
2552.710 63.365
2117.559 9.121
1894.998 24.229
1843.637 80.377
1527.072 698.496
1419.841 37.349
1393.955 -0.000
1322.639 252.876
1283.495 155.968
1221.212 -2.610
1187.901 696.565
1071.255 191.164
984.650 5.644
913.131
Wavenumber

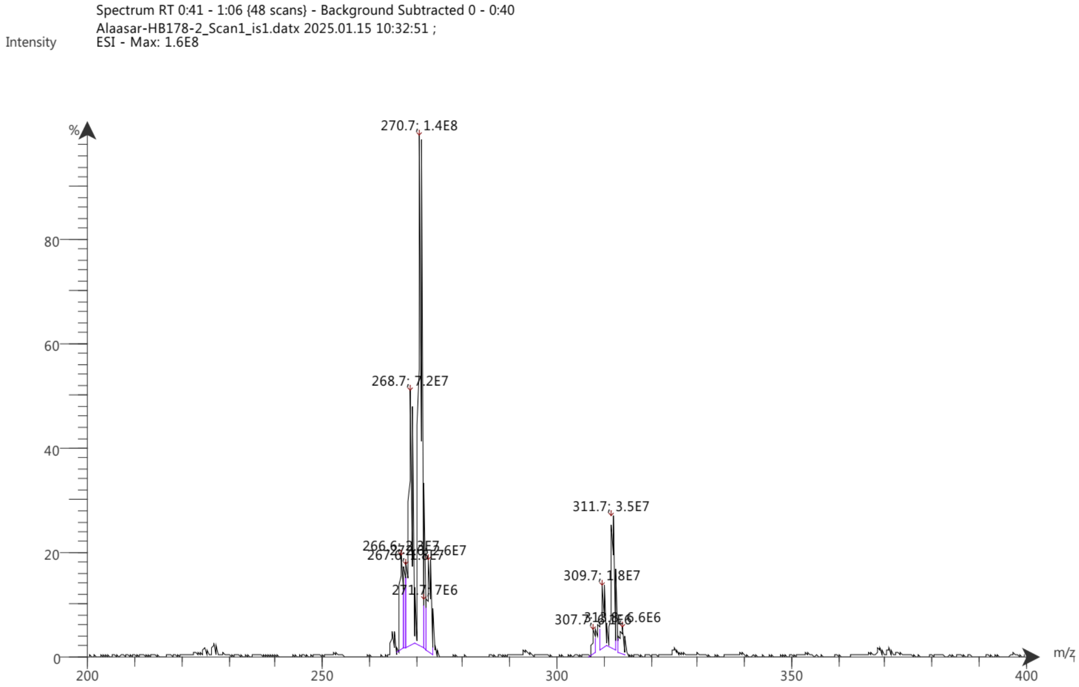
Spectrum RT 0:41 - 1:06 (48 scans) - Background Subtracted 0 - 0:40
Alaasar-HB178-2_Scan1_is1.datx 2025.01.15 10:32:51 ;
ESI - Max: 1.6E8
Intensity
%
270.7: 1.4E8
268.7: 7.2E7
311.7: 3.5E7
309.7: 1.8E7
m/z

4-((4-(Allylselanyl)phenyl)amino)-4-oxobut-2-enoic acid (HB179)

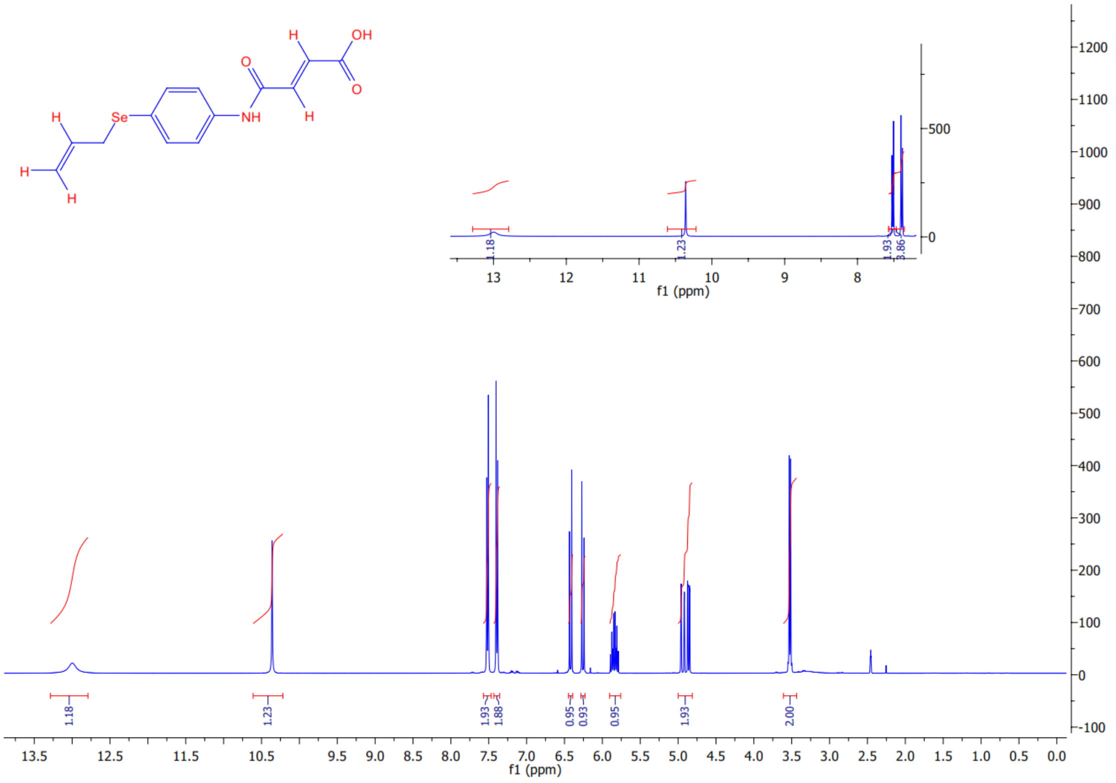

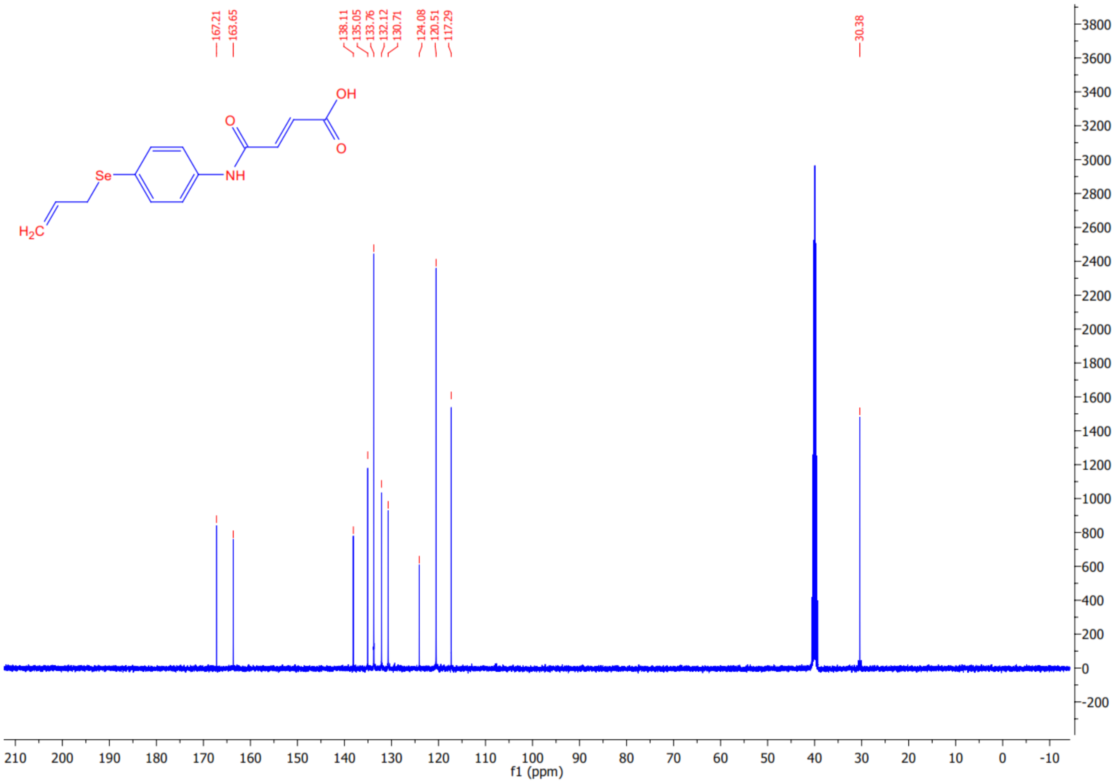

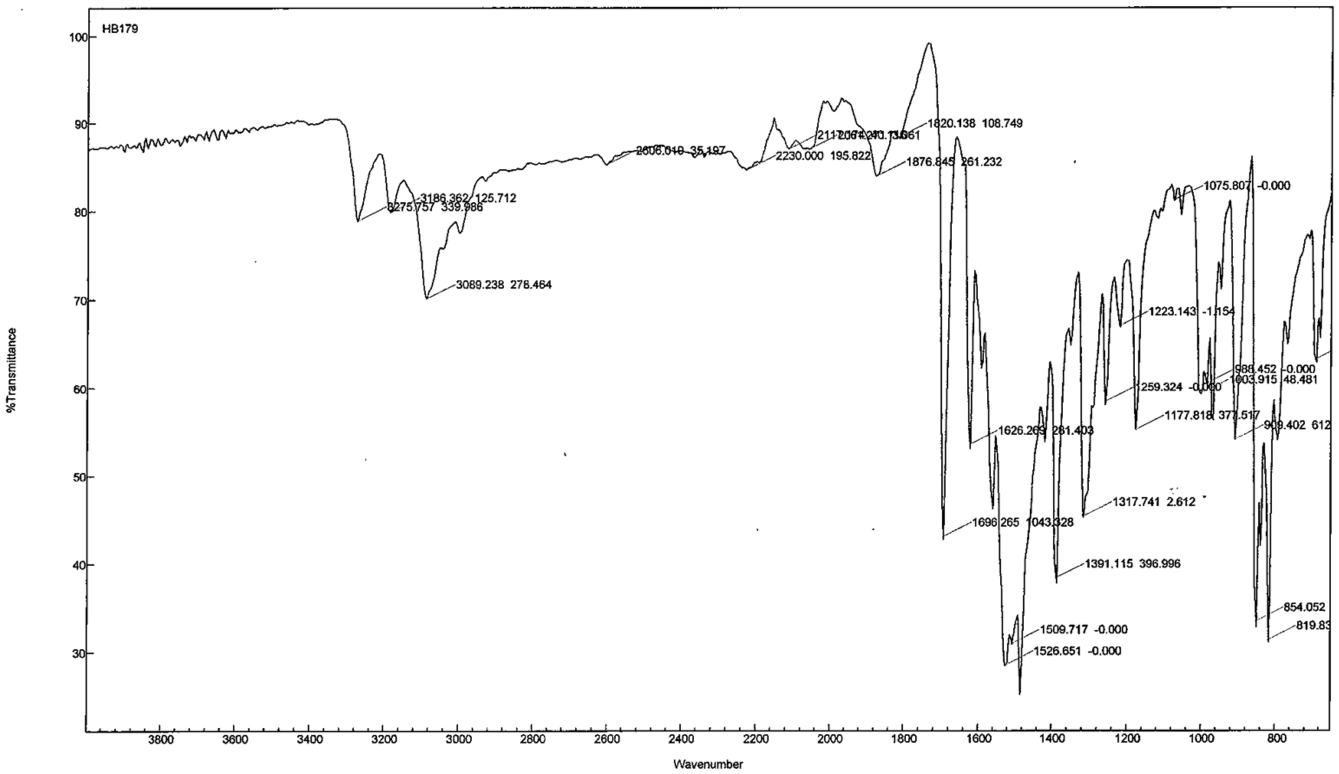
HB179
%Transmittance
Wavenumber
3275.757 339.986
3186.362 125.712
3089.238 276.464
2606.019 35.197
2230.000 195.822
1876.845 261.232
1820.138 108.749
1696.265 1043.328
1626.269 261.403
1509.717 -0.000
1526.651 -0.000
1391.115 396.996
1317.741 2.612
1259.324
1223.143 -1.154
1177.818 377.517
1075.807 -0.000
986.452 -0.000
1003.915 48.481
854.052

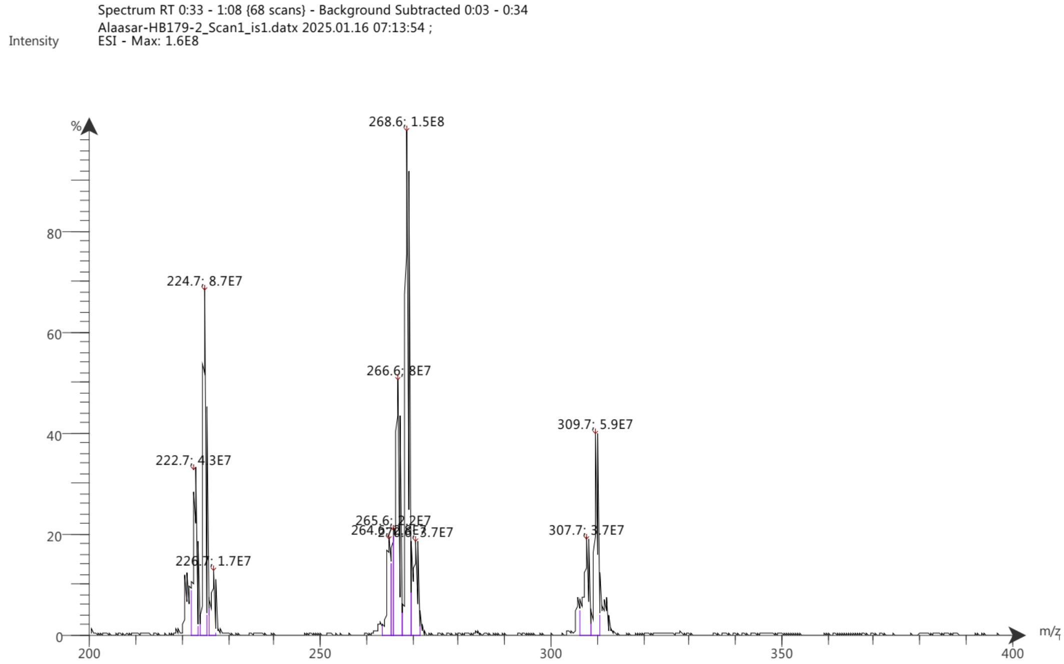
Spectrum RT 0:33 - 1:08 (68 scans) - Background Subtracted 0:03 - 0:34
Alaasar-HB179-2_Scan1_is1.datx 2025.01.16 07:13:54 ;
ESI - Max: 1.6E8
Intensity
268.6; 1.5E8
224.7; 8.7E7
266.6; 8E7
309.7; 5.9E7
222.7; 4.3E7
307.7; 3.7E7
226.7; 1.7E7
m/z

4-((4-(Allylselanyl)-3-(methoxycarbonyl)phenyl)amino)-4-oxobutanoic acid (HB181)

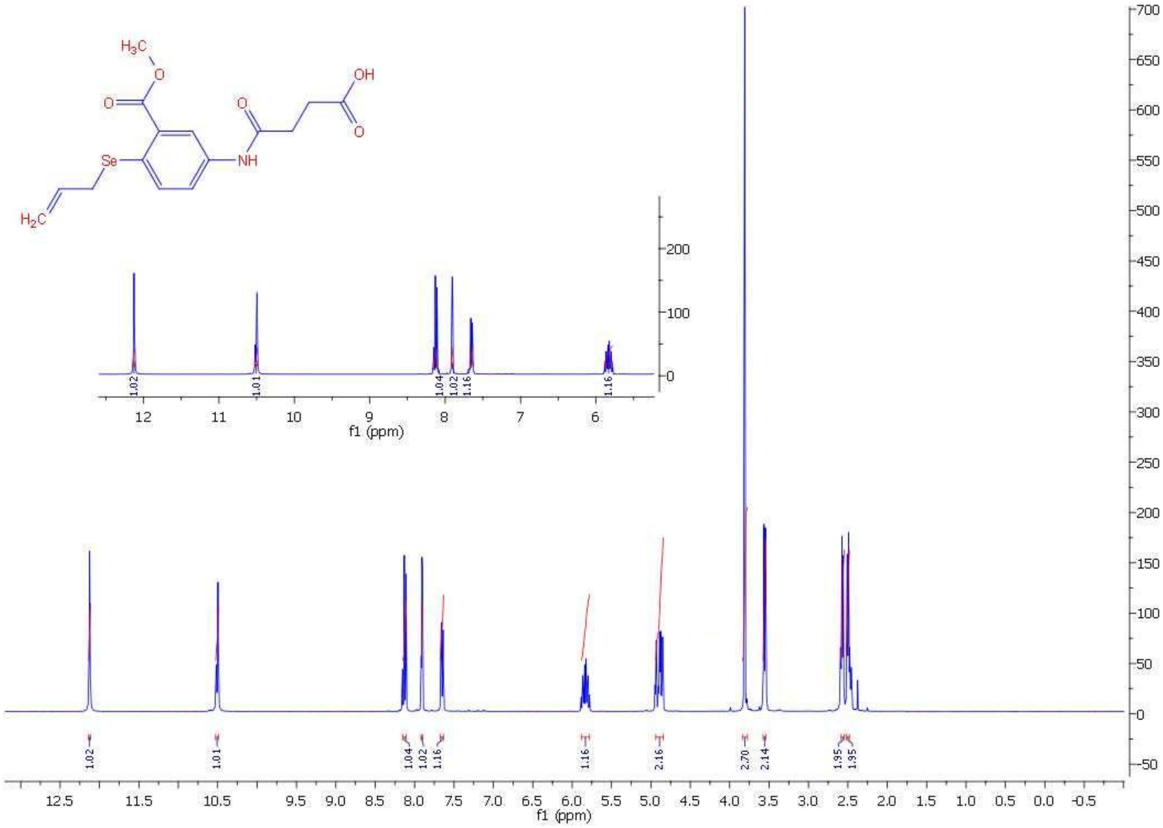

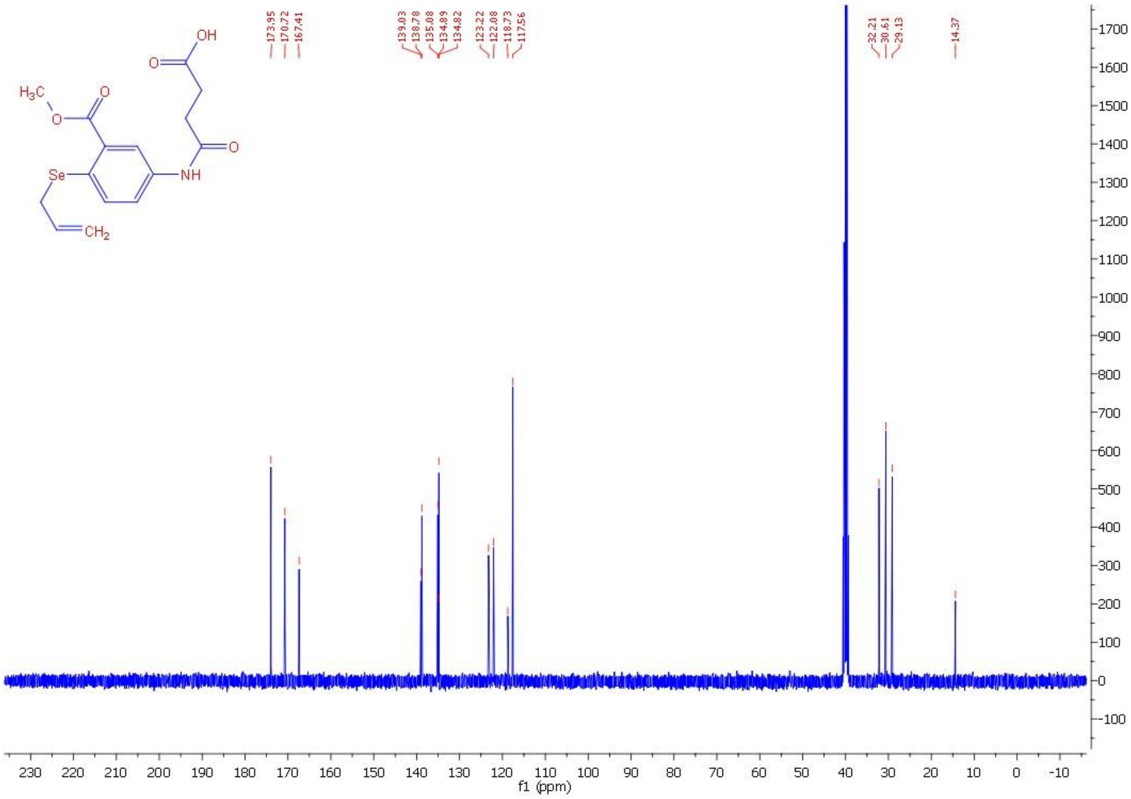

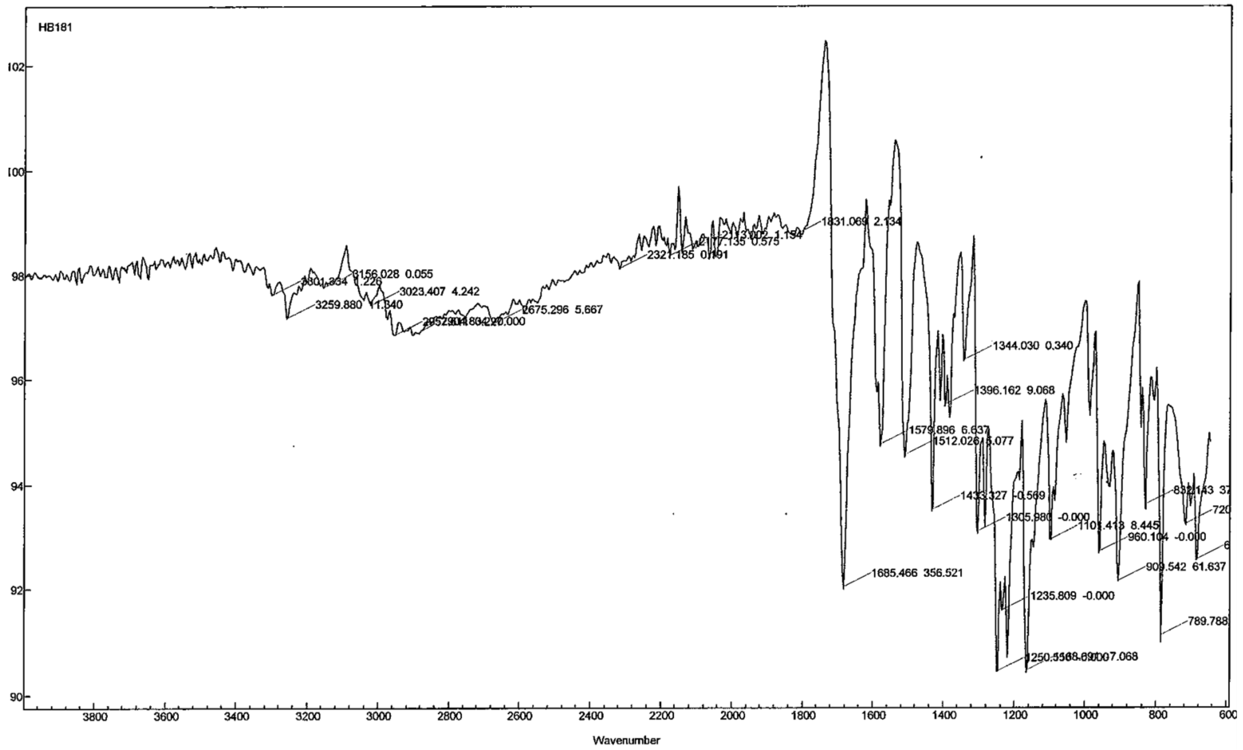
HB181
Wavenumber

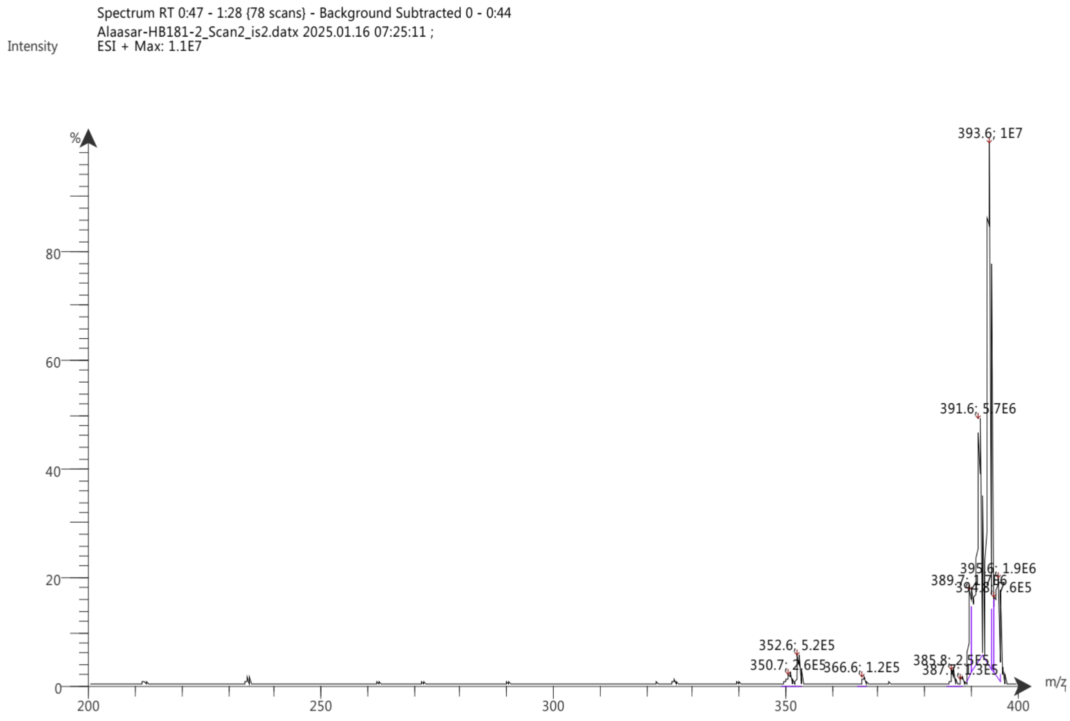
Spectrum RT 0:47 - 1:28 (78 scans) - Background Subtracted 0 - 0:44
Alaasar-HB181-2_Scan2_is2.datx 2025.01.16 07:25:11 ;
ESI + Max: 1.1E7
Intensity
393.6; 1E7
391.6; 5.7E6
352.6; 5.2E5
366.6; 1.2E5
m/z

4-((4-(Allylselanyl)-3-(methoxycarbonyl)phenyl)amino)-4-oxobut-2-enoic acid (HB183)

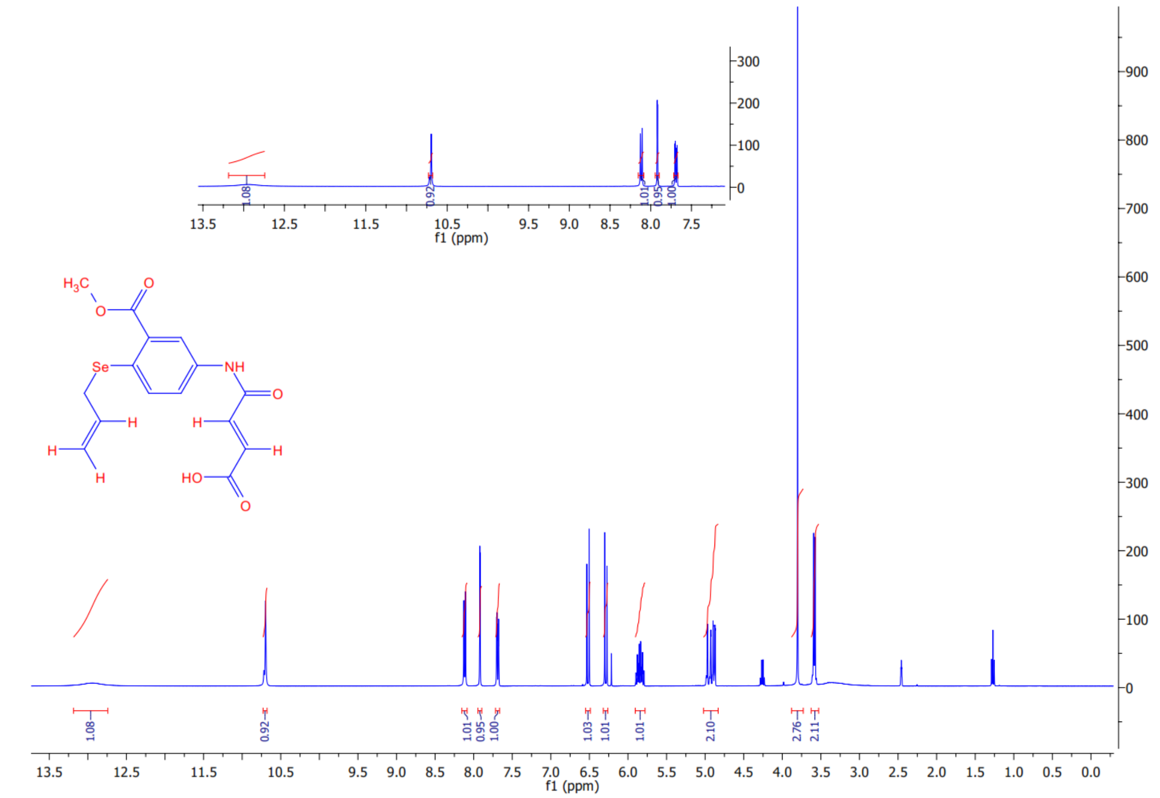

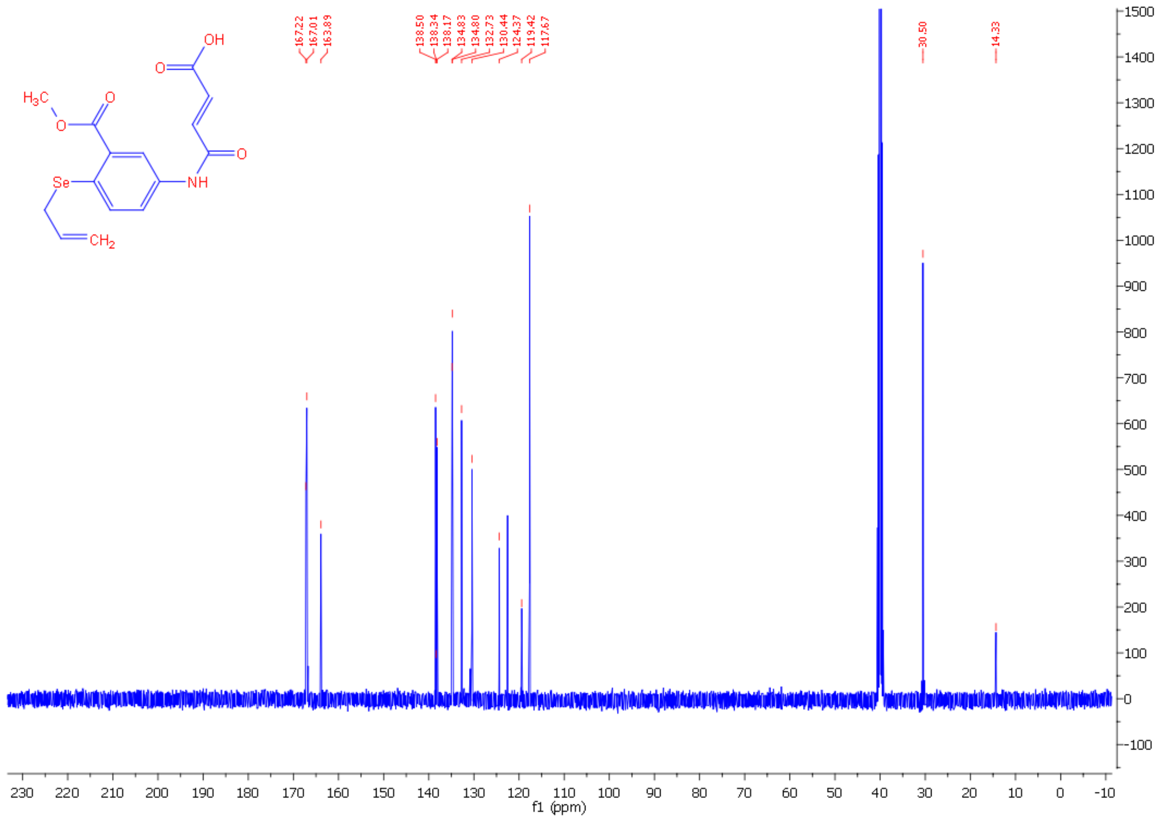

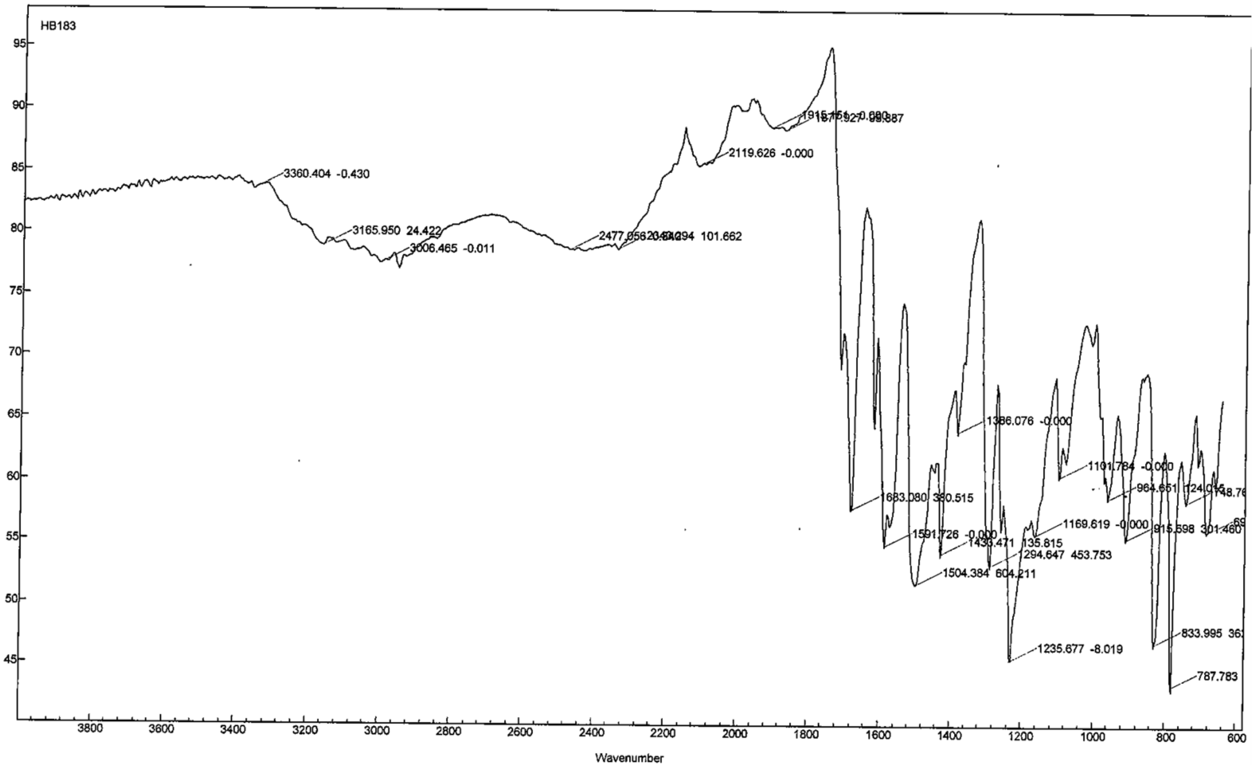
HB183
Wavenumber
3360.404 -0.430
3165.950 24.422
3006.465 -0.011
2119.626 -0.000
1235.677 -8.019
1504.384 604.211
1294.647 453.753
1169.619 -0.000
833.995
787.783

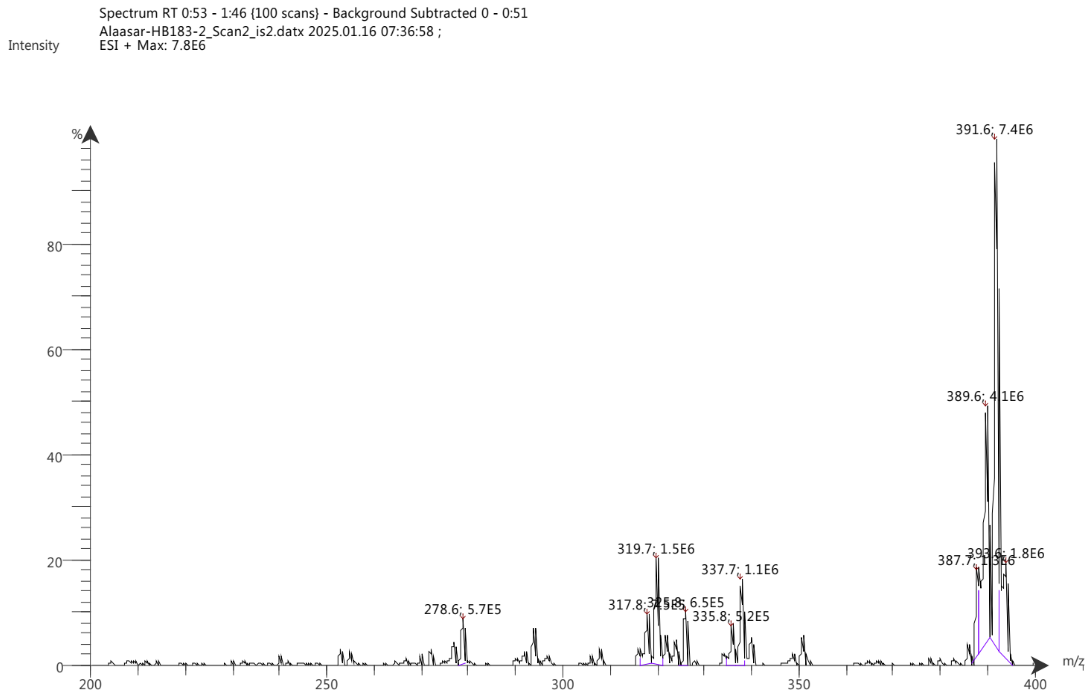
Spectrum RT 0:53 - 1:46 (100 scans) - Background Subtracted 0 - 0:51
Alaasar-HB183-2_Scan2_is2.datx 2025.01.16 07:36:58 ;
ESI + Max: 7.8E6
Intensity
391.6; 7.4E6
389.6; 4.1E6
319.7; 1.5E6
337.7; 1.1E6
278.6; 5.7E5
m/z

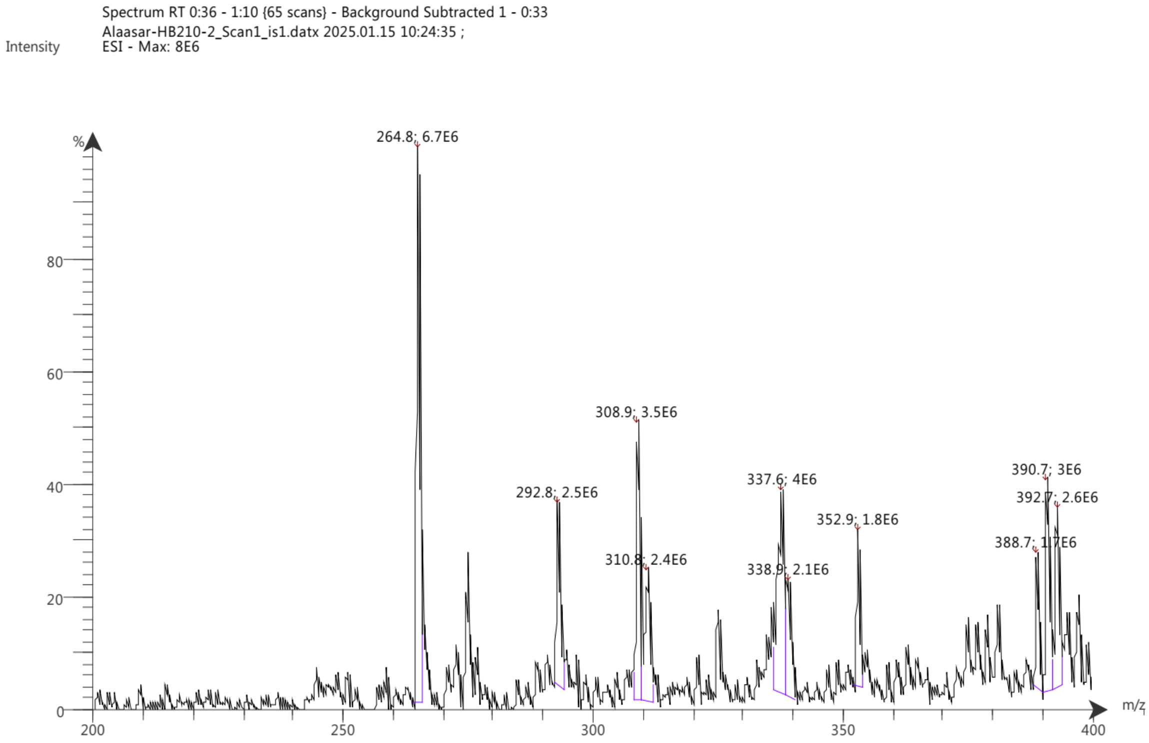
Spectrum RT 0:36 - 1:10 (65 scans) - Background Subtracted 1 - 0:33
Alaasar-HB210-2_Scan1_is1.datx 2025.01.15 10:24:35 ;
ESI - Max: 8E6
Intensity
%
264.8; 6.7E6
308.9; 3.5E6
292.8; 2.5E6
310.8; 2.4E6
337.6; 4E6
338.9; 2.1E6
352.9; 1.8E6
390.7; 3E6
392.7; 2.6E6
388.7; 1.7E6
m/z
0
20
40
60
80
200
250
300
350
400

Biological Evaluation

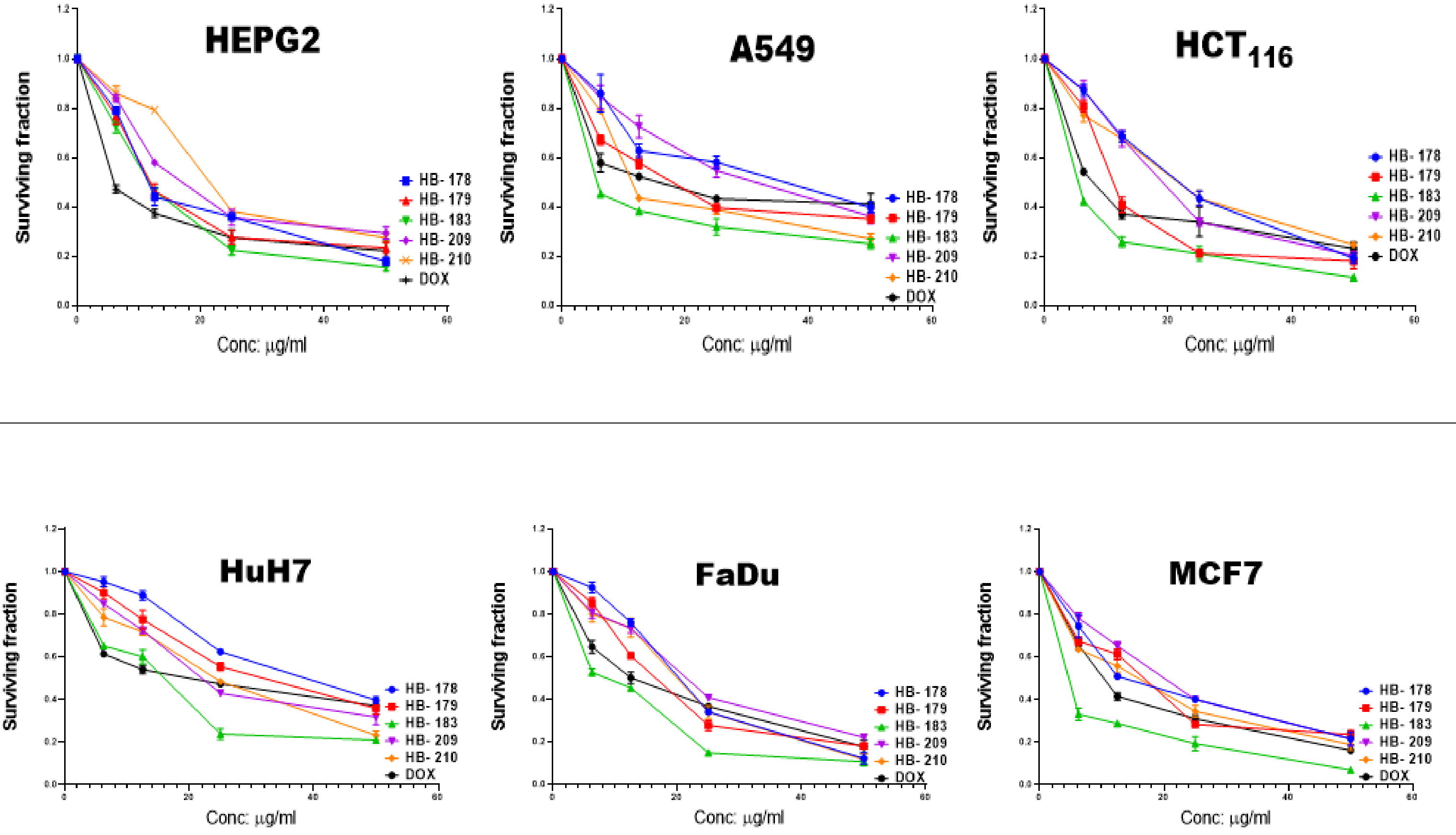

Detailed representation of the IC_{50} of each tested cell line with the evaluated compound.

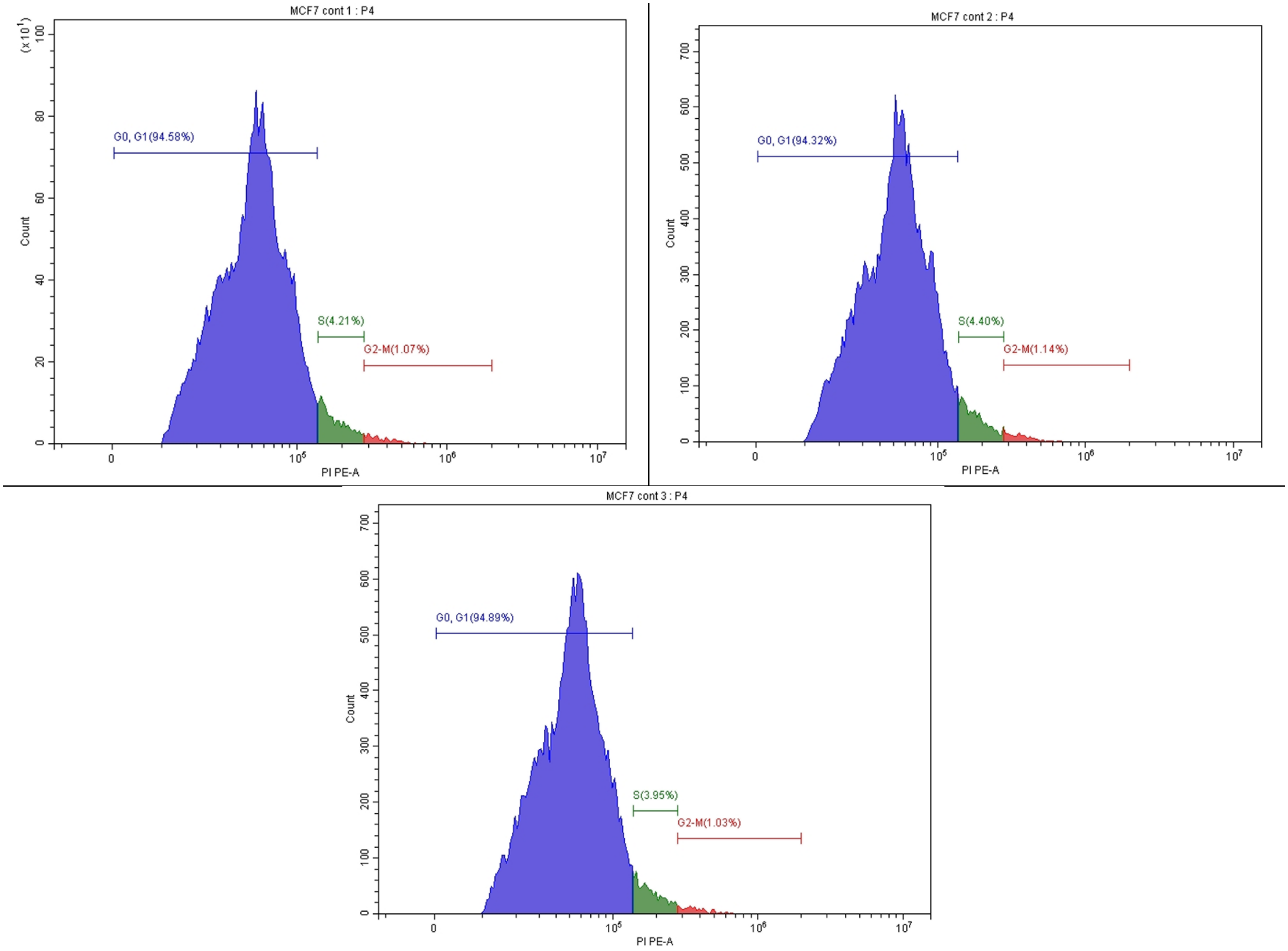

The triplicate experiments of the cell cycle analysis histogram of the untreated control MCF7 cancer cell line.

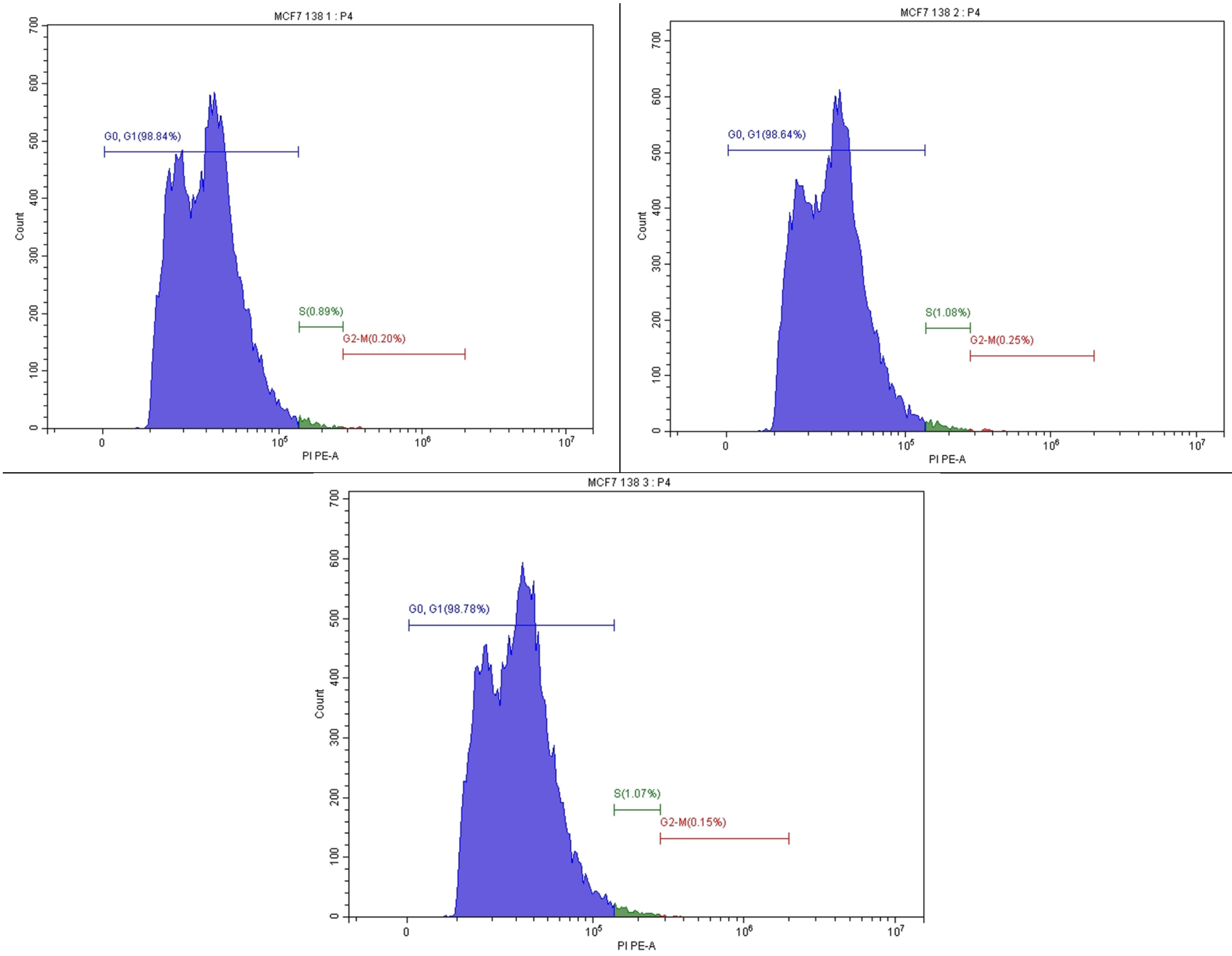

The triplicate experiments of the cell cycle analysis histogram of the HB183-treated MCF7 cancer cell line.

Docking Validation

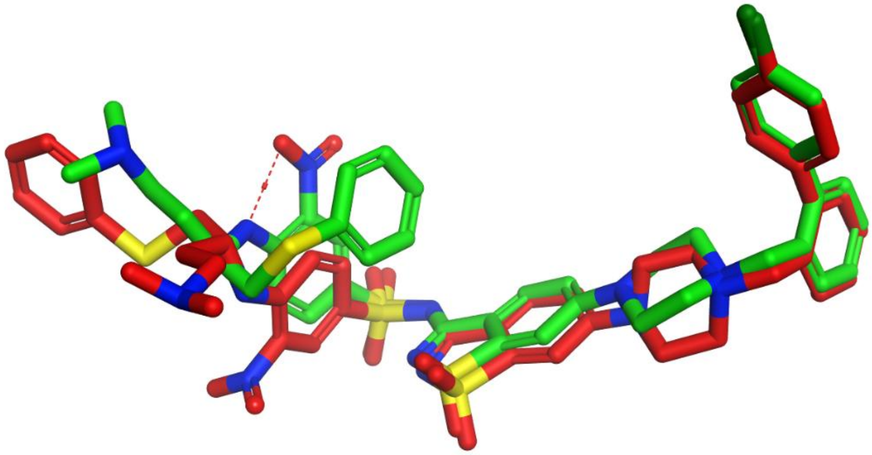

3D Superimposition of the redocked co-crystal (green) of the BCL-2 receptor (PDB ID: 4IEH) over the native one (red) with an RMSD value of 1.68 Å.

Molecular Dynamics Simulation

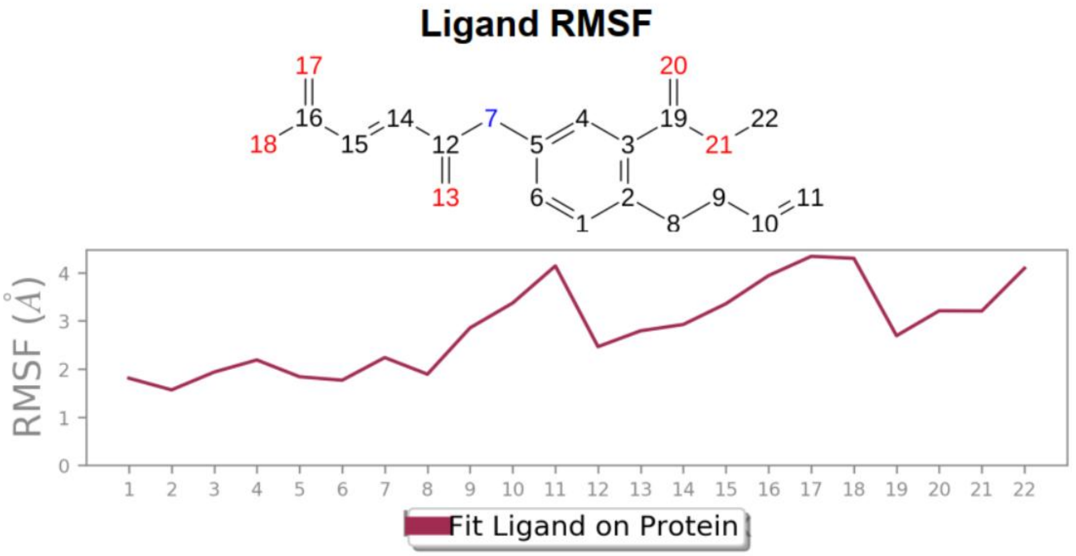

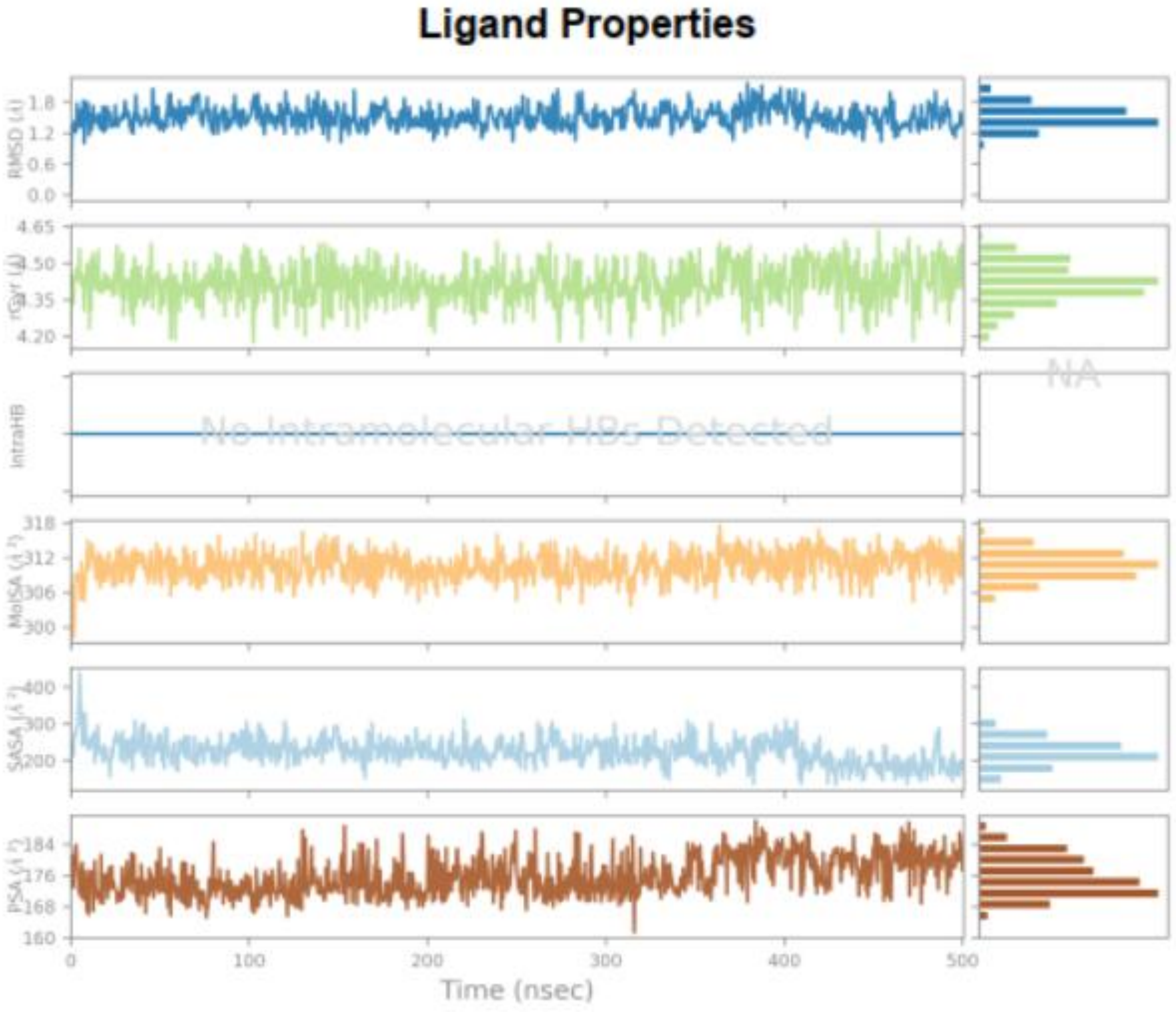

Molecular dynamics (MD) simulation

The molecular dynamics simulation was carried out using the Desmond simulation package of Schrödinger LLC.[5-7] The NPγT ensemble with the temperature 300 K and a pressure of 1.01 bar was applied in all runs. The simulation length was 500 ns with a relaxation time of 1 ps. The OPLS4 force field parameters were used in all simulations.[8] The cutoff radius in Coulomb interactions was 9.0 Å. The orthorhombic periodic box boundaries were set 10 Å away from the protein atoms. The water molecules were explicitly described using the transferable intermolecular potential with the three-point (TIP3P) model.[9] Salt concentration was set to 0.15 M NaCl and was built using the System Builder utility of Desmond. The Martyna−Tuckerman−Klein chain coupling scheme with a coupling constant of 2.0 ps was used for the pressure control, and the Nosé−Hoover chain coupling scheme for the temperature control.[10, 11] Nonbonded forces were calculated using a RESPA integrator where the short-range forces were updated every step, and the long-range forces were updated every three steps. The trajectories were saved at 300 ps intervals for analysis. The behavior and interactions between the ligands and protein were analyzed using the Simulation Interaction Diagram tool implemented in the Desmond MD package. The stability of MD simulations was monitored by looking at the RMSD of the ligand and protein atom positions as a function of simulation time.

MD trajectory analysis and prime MM-GBSA calculations

The simulation interactions diagram panel of the Maestro software was used to monitor interactions contribution in the ligand-protein stability. The molecular mechanics generalized born/solvent accessibility (MM – GBSA) was performed to calculate the ligand binding free energies and ligand strain energies for docked compounds over the last 50 ns with thermal_mmgbsa.py python script provided by Schrodinger which takes a Desmond trajectory file, splits it into individual snapshots, runs the MM-GBSA calculations on each frame, and outputs the average computed binding energy.

References

1. S. Shaaban, A. Negm, M. A. Sobh and L. A. Wessjohann, *European journal of medicinal chemistry*, 2015, **97**, 190-201.
2. C. Nitsche, V. N. Schreier, M. A. Behnam, A. Kumar, R. Bartenschlager and C. D. Klein, *Journal of Medicinal Chemistry*, 2013, **56**, 8389-8403.
3. B. Al-Abdallah, Y. S. Al-Faiyz and S. Shaaban, *Biomolecules*, 2022, **12**, 1765.
4. B. Al-Abdallah, Y. S. Al-Faiyz and S. Shaaban, *Inorganics*, 2022, **10**, 246.
5. K. J. Bowers, D. E. Chow, H. Xu, R. O. Dror, M. P. Eastwood, B. A. Gregersen, J. L. Klepeis, I. Kolossvary, M. A. Moraes, F. D. Sacerdoti, J. K. Salmon, Y. Shan and D. E. Shaw, 2006.
6. M. H. El-Shershaby, A. Ghiaty, A. H. Bayoumi, A. A. Al-Karmalawy, E. M. Husseiny, M. S. El-Zoghbi and H. S. Abulkhair, *Bioorganic & Medicinal Chemistry*, 2021, **42**, 116266.
7. D. E. S. Research, *Journal*, 2021.
8. E. Harder, W. Damm, J. Maple, C. Wu, M. Reboul, J. Y. Xiang, L. Wang, D. Lupyan, M. K. Dahlgren, J. L. Knight, J. W. Kaus, D. S. Cerutti, G. Krilov, W. L. Jorgensen, R. Abel and R. A. Friesner, *Journal of Chemical Theory and Computation*, 2016, **12**, 281-296.
9. W. L. Jorgensen, J. Chandrasekhar, J. D. Madura, R. W. Impey and M. L. Klein, *Journal of Chemical Physics*, 1983, **79**, 926-935.
10. G. J. Martyna, M. L. Klein and M. Tuckerman, *Journal of Chemical Physics*, 1992, **97**, 2635-2643.
11. G. J. Martyna, D. J. Tobias and M. L. Klein, *Journal of Chemical Physics*, 1994, **101**, 4177-4189.
